# Chemical and Antiplasmodial Investigations on *Carapa*-Derived Gedunin Derivatives and Semisynthetic 6- and 7‑Substituted Gedunin Analogues from the Brazilian Amazon

**DOI:** 10.1021/acsomega.5c04736

**Published:** 2025-09-29

**Authors:** Tiago Barbosa Pereira, Laís Garcia Jordão, Djalma da Silva Pereira, Gustavo Souza dos Santos, Daniel Soares dos Santos, Roberto Figliuolo, Jaqueline Siqueira da Costa, Leilane de Sousa Mendonça, Emersom Silva Lima, Marne Carvalho de Vasconcellos, Adrian Martin Pohlit

**Affiliations:** † Instituto de Ciências Exatas e da Terra, Programa de Pós-graduação em Química, Universidade Federal do Amazonas, Avenida General Rodrigo Otávio, 6200, Campus Universitário Senador Arthur Virgílio Filho, Setor Norte, Bloco 3 (ICE), Coroado I, CEP 69077-000 Manaus, Amazonas, Brazil; ‡ Instituto de Ciências Biológicas, Programa de Pós-graduação em Biotecnologia, Universidade Federal do Amazonas, Avenida General Rodrigo Octávio Jordão Ramos, 6200, Campus Universitário Senador Arthur Virgílio Filho, Setor Sul, Bloco M, Coroado I, CEP 69077-000 Manaus, Amazonas, Brazil; § Instituto Nacional de Pesquisas da Amazônia, Coordenação de Sociedade, Ambiente e Saúde, Avenida André Araújo, 2936, Petrópolis, CEP 69067-375 Manaus, Amazonas Brazil; ∥ Faculdade de Ciências Farmacêuticas, Programa de Pós-graduação em Inovação Farmacêutica, Universidade Federal do Amazonas, Avenida General Rodrigo Octávio, 6200, Campus Universitário Senador Arthur Virgílio Filho, Mini-campus, Coroado I, CEP 69080-900 Manaus, Amazonas , Brazil; ⊥ Instituto Nacional de Pesquisas da Amazônia, Coordenação de Tecnologia e Inovação, Avenida André Araújo, 2936, Petrópolis,CEP 69067-375 Manaus, Amazonas , Brazil

## Abstract

The aim of this work was to explore structure-antiplasmodial activity relationships among 6- and 7-acyloxy and hydroxy substituted gedunin derivatives. 7-Deacetyl-7-oxogedunin (cedrolide, **1**) and 6α-acetoxygedunin (**12**)—known antiplasmodial limonoids from the seeds of *Carapa guianensis* Aublet (Meliaceae)—were targeted for isolation at scale and used as starting materials for the preparation of a small, semisynthetic compound library that included gedunin (**4**), 7α- or 7β-acyloxy substituted 7-deacetylgedunins (**5**–**9**), and 6α-acyloxy substituted 7-deacetylgedunin derivatives (**13**–**15**). The concentrations of these compounds that inhibit 50% of the in vitro growth (IC_50_) of the multidrug-resistant K1 strain of the human malaria parasite, *Plasmodium falciparum*, were determined. Also, these compounds were screened for toxicity to MRC-5 human fibroblasts. The most antiplasmodial compounds featured a 7α-acetoxy or a 7β-acetoxy moiety (IC_50_ = 2.3–4.4 μM). Lower antiplasmodial activity was observed for gedunin derivatives exhibiting a 7α- or 7β-hydroxy, *O*-butanoyl, or *O*-pentanoyl moiety (and a C6 substituent). This study highlights the antiplasmodial effects, low toxicity to fibroblasts, and good selectivity (SI > 37) of 7-*epi*-gedunin (**5**), a compound that is available only through semisynthesis and whose antiplasmodial activity is reported for the first time.

## Graphical abstract

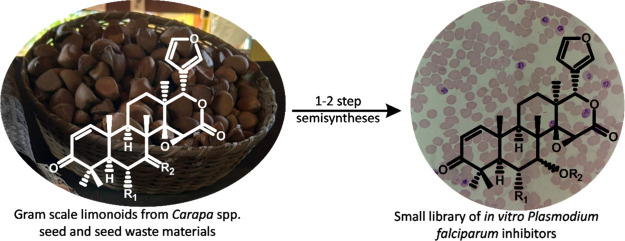

## Introduction

Gedunin (**4**) and its derivatives, such as 6α-acetoxygedunin (**12**) (Figure 1), are limonoids from the mahogany family (Meliaceae). These compounds are recognized for their significant antimalarial and other biological activities.^1–3^ Gedunin was first isolated from the heartwood of the tropical tree, locally known as “gedunohor” (*Entandrophragma angolense* C. DC.), in Nigeria.^4,5^ Gedunin has since been isolated from traditionally used antimalarial plants such as neem (*Azadirachta indica* A. Juss.),^6,7^ chinaberry (*Melia azedarach* L.),^6,8^ *Khaya grandifoliola* C. DC.^9^ and andiroba (*Carapa guianensis* Aublet).^10^ The in vitro antimalarial activity of gedunin was first described by Khalid and collaborators.^6,11^ Today, gedunin is recognized as an inhibitor of the in vitro growth (IC_50_ = 0.02–3.1 μM) of a variety of chloroquine-resistant (Dd2, K1, and W2) and chloroquine-sensitive (3D7, D6, D10, FCR3, and FCR3_TC_) *P. falciparum* strains.^9,12–19^ In early structure–activity studies, gedunin's 1,2-double bond, 3-keto group, and 7-acetoxy moieties were shown to be important structural elements linked to the high in vitro antiplasmodial activity observed for this compound.^12,14,20^

**Figure 1:**
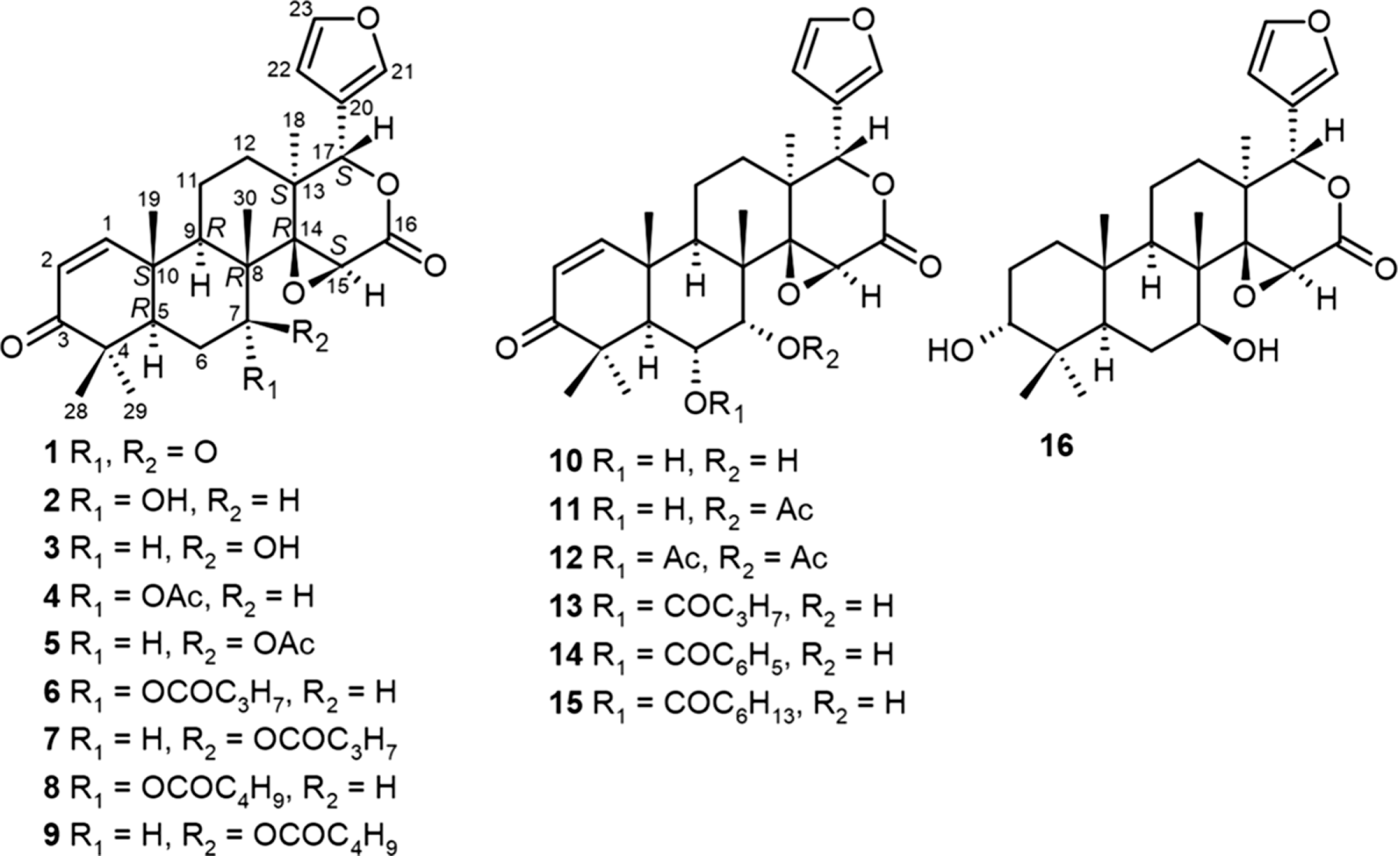
Structures of gedunin derivatives isolated from *Carapa* spp. (**1**, **11**, **12**) and semisynthetic gedunin (**4**) and gedunin derivatives (**2**, **3**, **5**–**10**, **13**–**16**).

In contrast to its in vitro antimalarial potential, gedunin (**4**) exhibited little to no in vivo activity in an established rodent malaria model. Thus, at oral or subcutaneous doses of 50–90 mg/kg/day in *Plasmodium berghei*-infected mice, gedunin failed to significantly suppress parasite growth compared to untreated controls in the Peters 4-day suppression test.^14,16^ The rationale for the lack of in vivo activity is that, under physiological conditions, gedunin (**4**) is metabolized by esterases to the generally less active 7-deacetylgedunin (**2**, IC_50_ = 1.3–5.9 μM in a variety of *P. falciparum* strains).^1,12,14,19,21^ In the laboratory, compound **2** was shown to react at low pH (similar to that found in mouse and human stomachs) to form an inactive, structurally defined (C7–C8 *seco*, epoxide ring opened) product.^20^ Given gedunin′s in vivo lability, a semisynthetic, esterase resistant derivative, 7-deacetyl-7α-methoxygedunin, was developed. This gedunin derivative inhibited *P. falciparum* in vitro (IC_50_ = 1.6–1.7 μM) and exhibited good stability at low pH. Furthermore, oral administration of a binary treatment comprised of 7-deacetyl-7α-methoxygedunin (50 mg/kg/day) and the cytochrome P450 inhibitor, dillapiole (25 mg/kg/day), resulted in significant suppression (68–81%) of *P. berghei* in mice compared to untreated controls.^14,20^

Over the past decade and a half, the promising antimalarial potential of *C. guianensis* seed and flower oils and 6-substituted gedunin derivatives, such as 6α-acetoxygedunin (**12**), has been revealed. Andiroba seed oil (ASO)―used as an antimalarial by indigenous and traditional communities in the Brazilian^22^ and Peruvian^23^ Amazon―inhibits the chloroquine-resistant *P. falciparum* W2 and Dd2 strains in vitro and is comprised of 6α-acetoxygedunin (**12**) and other antiplasmodial compounds related to gedunin.^10,24^ In the first report on its antiplasmodial activity, 6α-acetoxygedunin—isolated from *C. guianensis* flower oil—was shown to inhibit the *P. falciparum* FCR3 strain (IC_50_ = 2.8 μM).^13^ Shortly after, we showed that 6α-acetoxygedunin (**12**)—isolated from *C. guianensis* ASO—inhibited the *P. falciparum* K1 strain (IC_50_ = 7.0 μM).^24^ Importantly, a more recent study on *C. guianensis* found 6α-acetoxygedunin (**12**, IC_50_ = 2.1 ± 0.2 μM) to be more active than gedunin (**4**, IC_50_ = 2.8 ± 0.2 μM) against the *P. falciparum* Dd2 strain.^17^ Finally, we found that 6α-acetoxygedunin (**12**) suppressed parasitemia (40–66%) in *P. berghei*-infected mice at oral doses of 50–100 mg/kg/day and also exhibited a clear dose–response.^24^ Taken together, these studies provide evidence for the in vitro and in vivo antimalarial potential of 6-substituted gedunin derivative **12** and related compounds for further antimalarial development.

A couple of 6-substituted gedunin derivatives, structurally analogous to 6α-acetoxygedunin (**12**), have been evaluated for antiplasmodial activity in previous reports. Thus, 6α-acetoxy-7-deacetylgedunin was isolated from *C. guianensis* flower oil and shown to inhibit the *P. falciparum* FCR3 strain (IC_50_ = 4.0 μM).^13^ Also, we found that semisynthetic 6α-hydroxy-7-deacetylgedunin (**10**)―prepared from 6α-acetoxygedunin isolated from *C. guianensis*―inhibited the *P. falciparum* K1 strain (IC_50_ = 5.0 μM).^24^ It is worth noting that gedunin derivative **10** (7-*O*-acetyl absent) exhibited slightly greater antiplasmodial activity than compound **12** (7-*O*-acetyl present)^24^ and 6α-acetoxy-7-deacetylgedunin (no 7-*O*-acetyl) was found to be slightly less active than **12**.^13^ Thus, based on these findings, the relevance to antiplasmodial activity of a 7-*O*-acetyl moiety among 6-substituted gedunin derivatives was not demonstrated as it had been in the original work on the structure-antiplasmodial relationships among gedunin (**4**), 7-deacetylgedunin (**2**) and related compounds already described.^12,14,20^ In this context, given the in vitro and in vivo activity of 6α-acetoxygedunin (**12**) described above, we became interested in evaluating the effects of the size and chemical nature of 6-substituents on antiplasmodial activity. It is also noteworthy that previous work on the antiplasmodial activity of 7-substituted gedunin derivatives did not include 7-*epi*-gedunin (**5**) or 7-*O*-substituted 7-*epi*-gedunin derivatives (e.g., **7** and **9**). These observations point to the need for a better understanding of structure-antiplasmodial activity relationships in 6-substituted and 7-substituted gedunin derivatives which is the aim of the present work.

Herein, 7-deacetyl-7-oxogedunin (**1**) and 6α-acetoxygedunin (**12**) were isolated at scale from *C. guianensis* seeds, ASO and ASO-depleted seed residues by several methods. Gedunin derivatives **1** and **12** were the starting materials for the synthesis of a small compound library comprised of C6 and C7 hydroxy and acyloxy substituted gedunin derivatives **2**, **3**, **5**–**10**, **13**–**16** and gedunin (**4**). Gedunin and gedunin derivatives were then evaluated for their ability to inhibit the in vitro growth of the *P. falciparum* K1 strain to investigate the relationships between antiplasmodial activity and structural elements present in gedunin and derivatives, such as 6- and 7-substituent size and nature and C7 relative orientation.

## Results and Discussion

Extraction of *Carapa guianensis* seeds from Amazonas State, Brazil using different procedures provided a small quantity of 6α-hydroxygedunin (**11**) and multigram quantities of 7-deacetyl-7-oxogedunin (**1**) and 6α-acetoxygedunin (**12**) after preparative HPLC separation. Our results confirm those of Zelnick and collaborators who described gedunin derivatives **11** and **12** for the first time,^25^ along with the already known 7-deacetyl-7-oxogedunin (**1**),^25,26^ from the defatted seeds of *C. guianensis* from Amazonas State.

In general, the ^1^H and ^13^C NMR spectra of isolated gedunin derivatives **1**, **11**, and **12**—and all synthetic compounds related to gedunin except for compound **16**—shared several features related to the common structural elements found in these compounds. Thus, the ^1^H NMR spectra of **1**, **11**, and **12** exhibited characteristic signals of the furan ring at δ 7.40–7.50 (H21 and H23) and δ 6.30–6.40 (H22), a furfuryl singlet at δ 5.50–5.70 (H17), vinylic doublets (*J* = 10.1–10.2 Hz) at δ 7.05–7.20 (H1) and δ 5.90–6.00 (H2) corresponding to the α,β-unsaturated ketone moiety, a singlet corresponding to the oxirane α-hydrogen (H15) at δ 3.60–3.90 and five singlets corresponding to CH_3_ groups at δ 1.10–1.45 (H18, H19, H28–H30).^24^ The ^13^C NMR/DEPT spectra of these compounds featured signals corresponding to the conjugated ketone at δ 203.3–205.5 (s, C3), 156.0–156.3 (d, C1) and 126.0–126.7 (d, C2), the lactone C=O at δ 167.1–167.3 (s, C16) and >CHO– at δ 78.0–78.3 (d, C17), the furan ring at δ 143.1–143.2 (d, C23), 141.0–141.2 (d, C21), 120.2–120.3 (s, C20), 109.8–109.9 (d, C22) and the oxirane moiety at δ 65.2–69.8 (s, C14) and δ 53.6–56.4 (d, C15).^24^

Together with the common chemical shift and coupling data discussed above, analysis of chemical shift, multiplicity and other features of H5, H6, and H7 signals allowed for structural identification of isolated compounds **1**, **11**, and **12**. In this way, 7-deacetyl-7-oxogedunin (**1**) was identified by the characteristic chemical shifts and coupling of the H5 (δ_H_ 2.21, dd, *J* = 14.3, 3.0 Hz), H6α (δ_H_ 2.43, dd, *J* = 14.3, 3.0 Hz) and H6β (δ_H_ 2.94, *t*, *J* = 14.3 Hz) signals and the presence of a signal at δ_C_ 208.2 consistent with a C7 keto group and comparison to literature.^24^ The ^1^H spectrum of 6α-acetoxygedunin (**12**) featured singlets at δ 2.07 and 2.18 corresponding to two CH_3_CO groups. Compound **12** was structurally identified based on the *trans*-diaxial coupling of H5 (δ 2.56, d, *J* = 12.5 Hz) and H6 (δ 5.31, dd, *J* = 12.5, 2.4 Hz) and axial–equatorial coupling of H6 and H7 (δ 4.93, d, *J* = 2.4 Hz) as previously described.^24^ No NMR data are available for 6α-hydroxygedunin (**11**) in the literature.^25^ Structure identification of compound **11** was straightforward herein based on HRMS, 1D and 2D NMR spectrometric analysis. In the ^1^H spectrum, the relative orientation of H5α and carbinolic nature of H6β and H7β was evident from chemical shifts and *trans*-diaxial coupling (*J* = 11.9 Hz) of H5 (δ 2.25, d) and H6 (δ 4.29, dd) and axial–equatorial coupling (*J* = 2.3 Hz) of H6 and H7 (δ 4.78, d). The 7-OAc group was evidenced by signals at δ_C_ 172.5 and 21.2 and δ_H_ 2.22 (3H).

The synthetic methodology utilized is summarized in Figure 2. The main strategy involved the use of selective reagents and conditions that would lead initially to the modification of the functional groups at C7 in 7-deacetyl-7-oxogedunin (**1**) and C6 and C7 in 6α-acetoxygedunin (**12**). Sodium borohydride (NaBH_4_) is known to selectively reduce the C7 keto function in compound **1** at low temperatures while at higher temperatures, selectivity decreases and the C1–C2 olefin, C3 carbonyl and C16 lactone are also reduced. The reduction of compound **1** gives a mixture of epimeric 7-deacetylgedunin (**2**) and 7-deacetyl-7-*epi*-gedunin (**3**) that strategically underwent acylation reactions to provide series of 7α-acyloxygedunin (**4**, **6**, and **8**) and 7β-acyloxygedunin (**5**, **7**, and **9**) derivatives of increasing size at C7. Similarly, we and others have previously shown that the acetyl functions in compound **12** can be selectively cleaved under mild hydrolysis conditions that provide intermediate 6α-hydroxy-7-deacetylgedunin (**10**). The latter dihydroxy compound gave rise to a series of 6a-acyloxy-7-deacetylgedunin derivatives **13**–**15** of increasing size of the acyloxy group. This methodology produced a small compound library with sufficient quantities of gedunin and gedunin derivatives exhibiting structural variation at C7 and C6 for the study of the qualitative structure-antiplasmodial activity relationships among these compounds.

**Figure 2:**
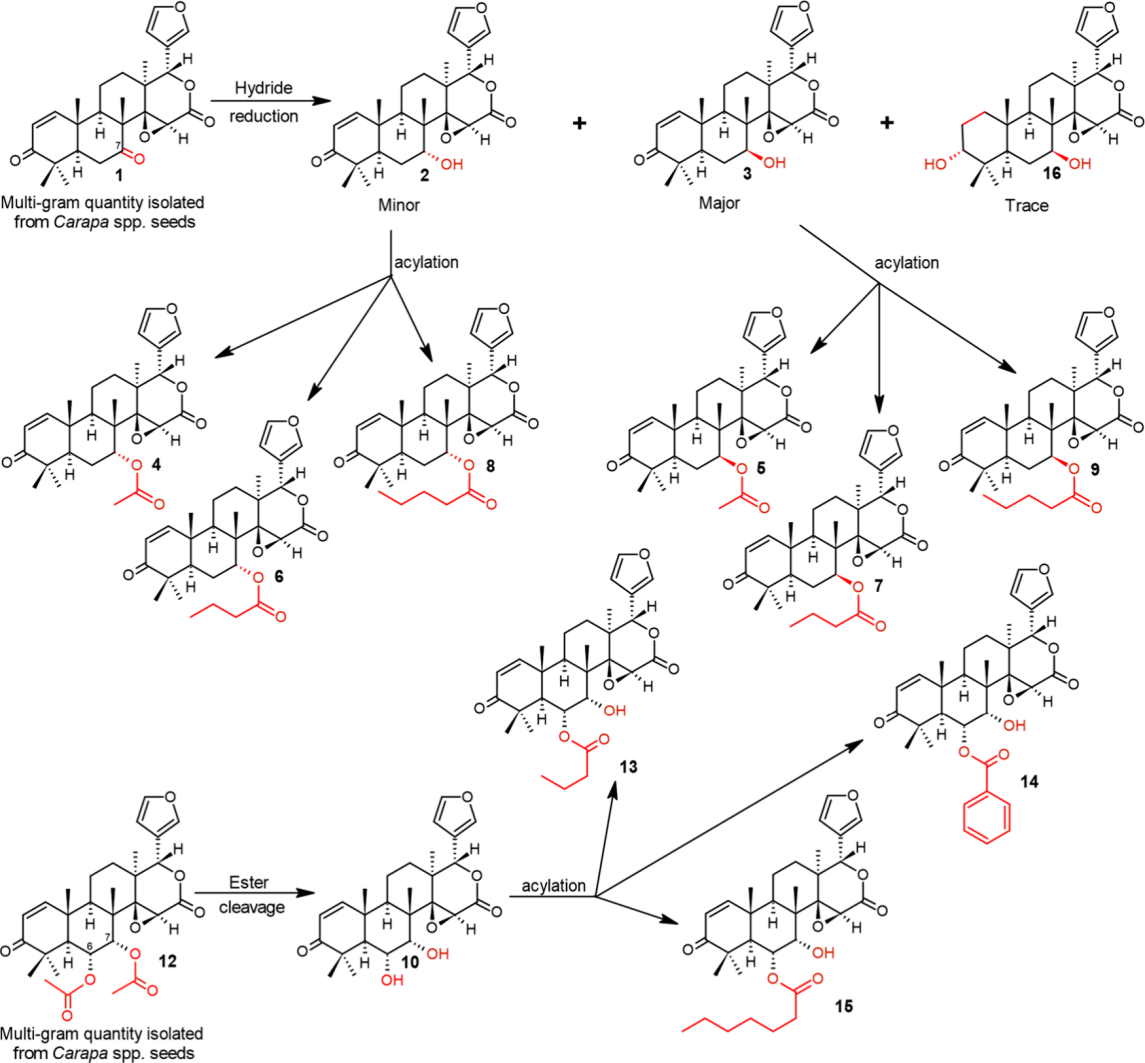
Methodology for the semisynthesis of gedunin (**4**) and 7-substituted gedunin derivatives **2**, **3**, **5**–**9**, **16** and 6-substituted gedunin derivatives **10**, **13**–**15** starting from the isolated limonoids **1** and **12**, respectively.

Dideacetylation of 6α-acetoxygedunin (**12**) resulted in the formation of 6α,7α-dihydroxygedunin derivative **10**.^24,25^ Monobutanoylation, monobenzoylation and monoheptanoylation of **10** occurred regioselectively at the 6α-hydroxyl group to provide low to fair yields (13.0–57.3%) of new monoesters **13**–**15** as detailed in the analysis of the spectrometric data below.

Products **13**–**15** exhibited HRMS, 1D and 2D NMR spectrometric data consistent with monoacylation products. ^1^H NMR chemical shift and coupling data for H6 (δ 5.32–5.60, dd, *J* = 12.3–12.4, 1.8–2.1 Hz) are consistent with *trans*-diaxial coupling with H5 (δ 2.74–2.90, *J* = 12.3–12.4 Hz) in these compounds. Also, axial–equatorial coupling (*J* = 0–2.0 Hz) is observed for H6 and H7 (δ 3.46–3.55). H5 and H6 (^3^ *J*) signals are also correlated in COSY spectra of these compounds, and correlations of side-chain carbonyl ^13^C signals (δ 168.1–172.6) with H6 (^3^ *J*) in HMBC spectra were all consistent with acylation of the 6α-hydroxy moiety in **10** (Figure 3A). The regioselective outcome presumably derives from the lower relative stereoelectronic effects/greater reaction kinetics and/or greater product stability associated with acylation of the equatorial 6α-OH. Presumably, once formed, the 6α-acyloxy moiety should also help impede acylation of the 7α-OH function. 7α-OH acylated products were not observed. A similar result was observed for the acetylation of 6α,7α,11β-trihydroxy-7-deacetylgedunin in acetic anhydride/pyridine that resulted in only the 6α,11β-diacetoxy,7α-hydroxy-7-deacetylgedunin product being observed.^27^

**Figure 3:**
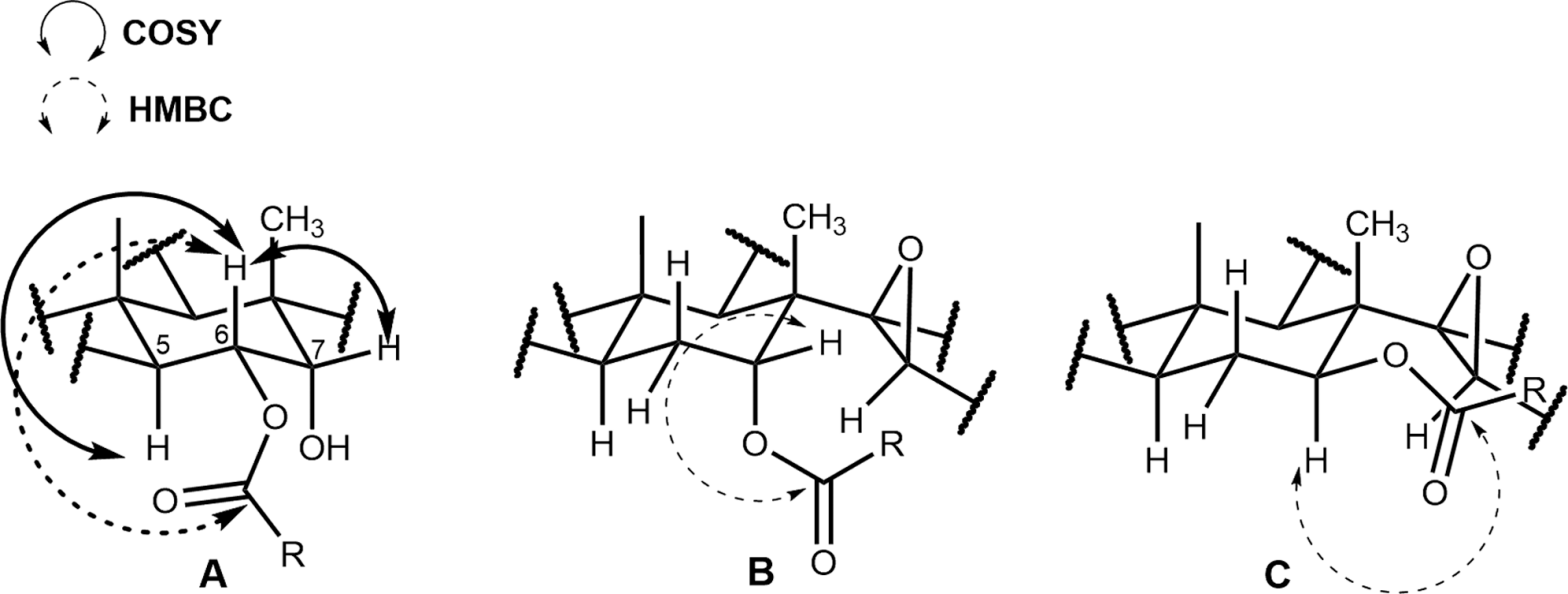
Three-bond (^3^ *J*) correlations observed in the 2D NMR spectra of semisynthetic products: (A) **13**–**15**, (B) **4**, **6**, and **8**, and (C) **5**, **7**, and **9**. Structure **B** illustrates the spatial proximity of H7 and the face of the epoxide moiety in 7α-acyloxy compounds.

Reduction of cedrolide (**1**) with NaBH_4_ yielded a separable mixture (40:7.5:1) of 7-deacetyl-7-*epi*-gedunin (**3**), 7-deacetylgedunin (**2**) and 1,2-dihydro-3α-hydroxy-3-deoxo-7-deacetyl-7-*epi*-gedunin (**16**, Figure 2). In work by others, NaBH_4_ reduction of **1** at lower temperature (−20 °C) provided a 9:1 mixture of **3** and **2**.^29^ This carbonyl reduction occurs via favored axial attack on the *Re* face of the C7 carbonyl of **1** providing 7β alcohol **3** as the major product.

7-deacetylgedunin (**2**) has been isolated previously from *C. guianensis* ASO from Pará^10^ and Amazonas^28^ States in Brazil. However, compound **2** was not detected in the *Carapa* spp. seeds or ASO studied herein. The ^1^H NMR spectrum of compound **2** prepared herein exhibited an H7 signal at δ 3.58 and other ^1^H and ^13^C NMR data comparable to the literature.^28,37,42^ The ^1^H NMR spectrum of compound **3** exhibited a doublet of doublets at δ 3.80 (H7) with a characteristic large coupling constant (*J* = 10.5 Hz) due to *trans*-diaxial coupling with H6β. ^1^H NMR for this compound were comparable to the literature.^29^

We found no previous report describing gedunin derivative **16**. An early report presenting no accompanying NMR data described the preparation of the 3β-epimer of **16**.^30^ In another report, NaBH_4_ reduction of 7-deacetyl-7-oxogedunin (**1**) under reflux resulted in a 2,3-dihydro-3β,7β-dihydroxy (lactone ring reduced) product. The ^1^H NMR spectrum of this product exhibited a doublet of doublets (*J* = 11.4, 4.3 Hz) at δ 3.20 that was assigned to H3 and was presumably due to *trans*-diaxial and axial–equatorial coupling to (H2β and H2α, respectively).^31^ In contrast, we assigned the 3α–OH orientation in reduction product **16** based on the H3 chemical shift (δ_H_ 3.50) and multiplicity (apparent triplet, *J* = 8.1 Hz) due to equatorial-axial (H2β) and equatorial-equatorial (H2α) coupling. In compound **16**, the ^1^H NMR chemical shift of H7 and *trans*-diaxial coupling of H7 and H6β were like those observed for 7-deacetyl-7-*epi*-gedunin (**3**)^29^ and a related 7-deacetyl-7-*epi*-gedunin reduction product.^31^ Based on these spectrometric similarities, we assigned the β-orientation to the 7-OH moiety in reduction product **16**.

Acetylation, butyrylation and pentanoylation of 7-deacetylgedunin (**2**) and 7-deacetyl-7-*epi*-gedunin (**3**) provided 26.9–63.0% yields of esterification products **4**–**9** (Figure 2). 7-*epi*-gedunin (**5**)^29^ and 7-*epi*-gedunin derivatives, such as **3**,^29^ **7** and **9**, have not been isolated from natural sources to date. The synthetic methodology adopted proved effective for producing enough 7-deacetyl gedunin and 7-*epi*-gedunin derivatives for evaluation of the antiplasmodial activity of these compounds.

Acetylation of pure 7-hydroxy compounds **3** and **2** provided 7-*epi-*gedunin (**5**) and gedunin (**4**), respectively, in 30 and 4.6% overall yields from **1**. Besides ^1^H and ^13^C signals related to the acetyl group, 7-*epi*-gedunin exhibited a characteristic H7 signal at δ 5.01 (dd) due to *trans*-diaxial coupling with H6β (*J* = 10.8 Hz) and axial–equatorial coupling (*J* = 4.5 Hz) with H6α.^29^ The H7 signal in gedunin (**4**) was a doublet of doublets (*J* = 3.3 and 2.1 Hz) at δ 4.56 due to equatorial-axial and equatorial-equatorial coupling with H6.^6^

Structural elucidation of esters **6**–**9** was straightforward based on chemical, ^1^H and ^13^C NMR, DEPT, HRMS and HMBC and HRMS data and comparison to NMR spectrometric data for **6** ^32^ in the literature. In the ^1^H and ^13^C NMR, DEPT and HSQC spectra of compounds **6** and **7** three sets of ^1^H and four ^13^C signals of the butanoyl moiety are observed as are similar signals for the pentanoyl function in the spectra of compounds **8** and **9**. In the ^1^H NMR spectra of compounds **6** and **8** the H-7 signal exhibits small coupling constants (*J* ≤ 3.3 Hz) expected for the α-relative orientation of the acyloxy moiety and in the ^1^H NMR spectra of compounds **7** and **9** the H-7 signals (dd) exhibit one large and one small coupling constant (*J* = 10.8, 4.4–4.5 Hz) consistent with *trans*-diaxial coupling with H6β and axial–equatorial coupling with H6α, respectively, and the β-relative orientation of the acyloxy moiety.

Multiplicities of H7 signals are consistent with a chair conformation in ring B of compounds **2**–**9**. Interestingly, the equatorial H7β in compounds **2** (δ 3.58; m) and **4**, **6** and **8** (δ 4.56–4.58, m, dd, or d*, J* ≤ 3.3 Hz, Figure 3B) are shielded compared to axial H7α in the corresponding epimers **3** (δ 3.81; dd, *J* = 10.5, 4.8 Hz), **5**, **7**, and **9** (δ 4.96–5.01, dd, *J* = 10.8, 4.4–4.5 Hz, Figure 3C). The shielding of the equatorial H7β in 7α-hydroxy and 7α-acyloxy compounds **2**, **4**, **6**, and **8** can be attributed to anisotropic effects associated with the proximity (ca. 2.5 Å, by HGS molecular structure modeling) of this atom to the face of the epoxide ring (Figure 3B).

Table 1 presents the results from the evaluation of isolated natural product **11**, semisynthetic gedunin (**4**), and 12 semisynthetic gedunin derivatives for in vitro inhibition of the growth of the K1 strain of *P. falciparum* and toxicity to fibroblasts. The results include the first known antiplasmodial activity data for known 7-deacetyl-7-*epi*-, 7-*epi*-, and 7-deacetyl-7-butanoylgedunins **3**, **5**, and **6**, respectively, and new 7-deacetyl-7-acyloxygedunins **7**–**9**, 7-deacetyl-6α-acyloxygedunins **13**–**15** and reduction product **16**.

**Table 1:** In Vitro 50% Inhibitory Concentrations (IC_50_) and 95% Confidence Intervals (CI_95_) for Isolated Limonoid **11** and Semisynthetic Derivatives against the K1 Strain of *Plasmodium falciparum* and MRC-5 Human Fibroblasts and Selectivity Indices (SI)

	**compound**	*P. falciparum* **IC**_**50**_ (**CI**_**95**_ **)**		**fibroblasts IC**_**50**_	**selectivity index**
**no.**	**name**	**μg/mL**	**μM**	×10^2^ **μM**	**(SI)**
**2**	7-deacetylgedunin	5.9 (4.4–7.9)	13.0 (9.9–18.0)	>1.1	>8.5
**3**	7-deacetyl-7-*epi*-gedunin	3.4 (2.6–4.5)	7.8 (6.0–10.0)	>1.1	>15
**4**	gedunin	1.4 (1.0–1.9)	2.8 (2.0–3.9)	>1.0	>37
**5**	7-*epi*-gedunin	1.1 (0.72–1.7)	2.3 (1.5–3.5)	>1.0	>45
**6**	7-deacetyl-7-butanoylgedunin	2.4 (2.1–2.7)	4.7 (4.1–5.3)	>0.98	>21
**7**	7-deacetyl-7-butanoyl-7-*epi*-gedunin	2.4 (1.9–3.1)	4.7 (3.7–6.1)	>0.98	>21
**8**	7-deacetyl-7-pentanoylgedunin	4.2 (3.3–5.4)	8.0 (6.3–10.0)	>0.95	>12
**9**	7-deacetyl-7-pentanoyl-7-*epi*-gedunin	3.7 (3.0–4.6)	7.0 (5.8–8.5)	>0.95	>14
**10**	7-deacetyl-6α-hydroxygedunin	2.8 (2.3–3.4)	6.1 (5.1–7.4)	>1.0	>16
**11**	6α-hydroxygedunin	2.2 (1.8–2.6)	4.4 (3.7–5.2)	>1.1	>26
**13**	7-deacetyl-6α-butanoyloxygedunin	3.7 (2.8–4.9)	7.1 (5.3–9.4)	>1.0	>15
**14**	7-deacetyl-6α-benzoxygedunin	5.1 (3.1–8.5)	10.0 (6.2–17.0)	>1.1	>11
**15**	7-deacetyl-6α-heptanoyloxygedunin	5.4 (4.4–6.6)	9.4 (7.7–12.0)	>0.88	>9.4
**16**	7-deacetyl-3-deoxo-1,2-dihydro-3α-hydroxy-7-*epi*-gedunin	16.0 (12.0–21.0)	35.0 (26.0–47.0)	>1.1	>3.2
	chloroquine diphosphate	0.18 (0.10–0.33)	0.35 (0.19–0.36)		
	quinine hydrochloride	0.14 (0.070–0.26)	0.35 (0.18–0.64)		
	doxorubicin			4.3 (3.7–5.0) nM	

In general, active substances inhibit the in vitro growth of *P. falciparum* in the nanomolar to low micromolar range (e.g., IC_50_ ≤ 10 μM)^33^ whereas inactive compounds present higher median inhibition concentrations (e.g., IC_50_ > 20 μM).^34^ IC_50_ values for isolated gedunin derivative **11**, semisynthetic gedunin (**4**) and 12 gedunin derivatives **2**, **3**, **5**–**10**, **13**–**15**, and **16** ranged from 2.3 to 35 μM. Twelve compounds were considered to be active and two were found to be inactive (compounds **2** and **16**, IC_50_ > 10 μM, Table 1).

Among the 6-substituted gedunin derivatives investigated, 6α-hydroxygedunin (**11**, IC_50_ = 4.4 μM) exhibited the greatest inhibition of the in vitro growth of against the K1 strain of *P. falciparum*. This contrasts with another report that found compound **11** from *C. guianensis* to be inactive (IC_50_ = 90 μM) against the FCR3 strain of *P. falciparum*.^13^ Compound **11** was the only 6-substituted derivative exhibiting a 7-acetoxy moiety evaluated in the present study. Other 6-substituted derivatives (**10**, **13**–**15**) featured a 7α-OH group and lower antiplasmodial activity (IC_50_ = 6.1–10 μM).

The relative orientation of the C7 acyloxy groups in the gedunin derivatives studied had no influence on the in vitro inhibition of *P. falciparum* K1 strain. However, among compounds **4**–**9**, antiplasmodial activity was inversely proportional to the size of the C7 acyloxy groups: CH_3_CO_2_– (IC_50_ = 2.3 and 2.8 μM, **5** and **4**, respectively) > CH_3_CH_2_CH_2_CO_2_– (IC_50_ = 4.7 μM, **6** and **7**) > CH_3_CH_2_CH_2_CH_2_CO_2_– (IC_50_ = 7.0 and 8.0 μM, **9** and **8**, respectively). In contrast, the presence of a C7α or C7β OH group was associated with low inhibitory activity (IC_50_ = 7.8 μM, **3**) compared to that observed for 7-acyloxy derivatives **4**–**9** or the absence of inhibitory activity (IC_50_ = 13 μM, **2**). Overall, the highest antiplasmodial activity was observed for derivatives exhibiting a C7 acetoxy group and no C6 substituent (gedunin (**4**, IC_50_ = 2.8 μM) and 7-*epi*-gedunin (**5**, IC_50_ = 2.3 μM)).

The lack of antiplasmodial activity observed for reduction product **16** (IC_50_ = 35 μM) is attributable to the absence of α,β-unsaturated 3-keto and 7-acetoxy moieties in the molecular structure of this compound.^12,14,20^ Similarly, compound **2** also lacks a 7-acetoxy group and was inactive against the K1 strain of *P. falciparum* (Table 1). Surprisingly, according to several previous reports, compound **2** demonstrated significant inhibition of the in vitro growth of the 3D7, D6, D10, W2, INDO (IC_50_ = 1.3–5.9 μM)^1,12,14,19^ and K1 (IC_50_ = 3.1 μM)^21^ strains of *P. falciparum*. Cedrolide (**1**) also lacks a C7 acetoxy function and in previous reports, this compound was observed to inhibit the in vitro growth of the D6 and W2 (IC_50_ > 20.7 μM),^12^ K1 (IC_50_ = 4.1 ^21^ and 20.7 ^24^ μM), FCR3 and INDO (IC_50_ = 2.5–7.5 μM)^1,13,35^ strains of *P. falciparum* to a lesser degree than gedunin.

Pure enantiomeric forms of gedunin have been isolated from different plant species, as evidenced by specific rotation ([α]_D_ was registered in CHCl_3_ in all the references discussed below) and X-ray crystallography data. Both gedunin enantiomers (and derivatives) exhibit antiplasmodial activity. Thus, (−)-gedunin from *Melia* azedarach^6,8^ and *Azadirachta indica* ^6^ barks has been found to inhibit the growth of *P. falciparum* (IC_50_ = 1 μM after 48 h; 0.3 μM after 96 h)^6^ and gedunin from *A. indica* fruit inhibited the D10 and W2 strains of *P. falciparum* (IC_50_ = 1.66 ± 0.37 and 1.31 ± 0.42 μM, respectively).^19^ (−)-gedunin isolated from *Cedrela odorata* was identified by comparison to literature data^4^ and exhibited activity (IC_50_ = 0.041–0.65 and 0.081–0.63 μM, respectively) against *P. falciparum* W2 and D6 strains as did semisynthetic derivatives of this compound.^12,14^ The 5*S*,7*S*,9*S*,8*R*,10*S,*13*R,*14*S,*15*R,*17*R* absolute configuration for this gedunin enantiomer from *Trichilia pallida* Sw. was also confirmed by X-ray crystallographic analysis.^36^ (+)-Gedunin and C11 hydroxy gedunin epimers were isolated from *Toona sinensis* (A. Juss.) M. Roem (as the synonym *Cedrela sinensis* Juss.) and X-ray crystallography established the absolute configuration.^37^ Herein, (+)-gedunin was semisynthesized from (−)-cedrolide (**1**) whose absolute configuration (see Figure 1) was determined previously by derivatization followed by X-ray crystallographic analysis.^31^ (+)-gedunin inhibited the *P. falciparum* K1 strain (Table 1) as did gedunin of undefined origin or specific rotation (IC_50_ = 1.5 μM, CI_95_ = 0.52–2.7 μM)^16^ and gedunin isolated from *C. guianensis* inhibited the FCR-3 (IC_50_ = 2.5 μM)^13^ and Dd2 (IC_50_ = 2.8 ± 0.2 μM)^17^ strains of *P. falciparum*. Specific rotation data are not available in several reports on the antiplasmodial activity of gedunin and gedunin derivatives.^15,16,18^ The existence of pure enantiomeric forms of gedunin and gedunin derivatives in nature exhibiting antiplasmodial activity has implications for future work toward the development of the antimalarial potential of this class of compounds.

Gedunin and gedunin derivatives exhibit selective toxicity to human and murine tumor cell lines according to literature reports. Cedrolide, 6α-acetoxygedunin, and gedunin are toxic to mouse mammary carcinoma (FM3A) cell cultures^13^ and the latter compound exhibits toxicity to human cervical cancer (KB) cell cultures.^12^ Gedunin induces apoptosis in cancer cells selectively and exhibits no appreciable toxicity to immortalized, adult-derived normal Hs578Bst cells or human mammary epithelial cells (HME).^38^ Similarly, our previous evaluation of compounds **1** and **12**,^24^ and herein of gedunin (**4**, Table 1), found no significant toxicity to the MRC-5 line of normal human fibroblasts (IC_50_ > 0.19, 0.11, and >0.10 mM, respectively). Furthermore, the gedunin derivatives assayed herein exhibited low cytotoxicity (IC_50_ > 88 μM). Active antiplasmodial compounds (those exhibiting IC_50_ ≤ 10 μM against *P. falciparum*) exhibited good selectivity (>9.4, Table 1). In general, selectivity >10 is considered satisfactory^39,40^ and greater selectivity can be useful for the identification of potential antimalarial compounds.^41^ Herein, gedunin (**4**) and 7-*epi*-gedunin (**5**) exhibited the greatest inhibition of the in vitro growth of *P. falciparum* and selectivity (SI > 37).

Future work should include comparative study of the antiplasmodial activity of the pure enantiomeric forms of gedunin and gedunin derivatives available from *Carapa* and plants belonging to other genera of the Meliaceae. Also, gedunin and 7-*epi*-gedunin derivatives exhibiting other functional groups, e.g., 7-*O*-ethers and 6-*O*-ethers, should also be targeted for synthesis and evaluation of the in vitro antiplasmodial activity of these compounds.

## Conclusions

Important in vitro antimalarial activity and good selectivity were found for semisynthetic 7-*epi*-gedunin (**5**) that is comparable to that of gedunin (**4**). Future work should evaluate 7-*epi*-gedunin (**5**) and 7-*epi*-gedunin derivatives in *P. berghei*-infected mice as isolated molecules and formulations.

## Experimental Section

### General Experimental Procedures

Pretreatment and purification of technical and analytical grade solvents used for extraction and chromatographic separation followed established procedures. Tedia Brasil (Rio de Janeiro) supplied HPLC-grade solvents. Merck supplied silica gel 60 (0.040–0.063 or 0.063–0.20 mm mesh) for column chromatography (CC). Analytical and preparative HPLC analyses were carried out at the National Institute for Amazon Research's (INPA) Amazon Active Principles Laboratory (LAPAAM). Pure gedunin derivatives were obtained from semipurified fractions by preparative HPLC-PDA-RID (high performance liquid chromatography-photodiode array detection-refractive index detection). Similarly, analytical samples of semisynthetic compounds were obtained from crude product mixtures by preparative HPLC-PDA-RID. Samples were filtered prior to analysis to remove particulate matter (Millipore Millex-HV 0.45 μm). Introduction of sample into the injection port was performed using a syringe (Hamilton, no. 3, blunt). For preparative applications, the Shimadzu HPLC system consisted of a 1 mL injection loop, twin LC-6AD pumps, flow splitter, SPD-M20A photodiode array (PDA) and RID-20A refractive index (RID) detectors with deuterium and tungsten lamps, CBM-20A system controller and a shim-pack semipreparative reverse-phase C-18 column (length × diameter: 250 × 20 mm, particle size: 5 μm).

Spectrometric characterization of isolated and semisynthetic compounds was performed at the Natural Products Analysis Laboratory (CA-LTQPN) at INPA. 1D and 2D NMR spectra were recorded on a Bruker Biospin Fourier (7.0 T) spectrometer operating at 300 (^1^H) and 75 (^13^C) MHz. Deuterated solvents were purchased from Sigma-Aldrich. Liquid chromatography-high resolution time-of-flight mass spectrometric analyses (LC-ESI-HRMS) were performed on a Shimadzu ultrafast liquid chromatograph (UFLC-PDA) coupled to a Bruker-Daltronics MicrOTOF-QII mass spectrometer featuring an electrospray ionization (ESI) source. Chromatographic purity analysis and spectrometric characterization of isolated and semisynthetic compounds was performed using UFLC-PDA (190–400 nm)-ESI-(+)-HRMS and 1D and 2D NMR techniques.

Specific rotation ([α]_D_^26^) analyses were performed at the Ribeirão Preto Faculty of Pharmaceutical Sciences (FCFRP) of the University of São Paulo (USP), Ribeiro Preto, São Paulo State, Brazil. Optical rotations of isolated and synthetic compounds were measured on a Jasco model P-2000 polarimeter (Japan) equipped with a 3.5 × 50 mm cylindrical glass cell (Jasco Parts Center, USA). The polarimeter was operated in manual mode at 26 °C using the D lines of a Na lamp (λ = 589 nm). Pure samples were analyzed as solutions in HPLC grade CHCl_3_ (Honeywell–Riedel-de Haen 99.99%). [α]_D_^26^ values reported herein are averages of 3 readings ± standard deviation. Literature (lit.) [α]_D_ values for compounds analyzed in CHCl_3_ were used for comparisons and in the discussion.

### Research Registry with Brazilian Regulatory Authority

This study is documented at the Brazilian Ministry of Environment′s platform Sisgen–Sistema Nacional de Gestão do Patrimônio Genético e do Conhecimento Tradicional Associado (http://www.sisgen.gov.br) under the registry numbers AAA58D3 and AA2D091.

### Plant Materials, Extraction and Purification Procedures

Isolation of gedunin derivatives **1**, **11**, and **12**. Seed material collection for this work occurred in three locations in Amazonas State, Brazil from February to May, 2014 in Carauari Municipality (S 4° 54′ W 66° 55′), on March 12, 2015 in Parintins Municipality (S 2° 38′ 15″ W 56° 43′ 44”) and on April 29, 2015 in Manaus Municipality (S 2° 52′ 59.988″ W 59° 58′ 0.012″). Three distinct methods were applied to these seed materials (see Supporting Information) and provided limonoid rich fractions (LRFs) as intermediates. Further purification steps applied to the LRFs resulted in the isolation of multigram quantities of cedrolide (**1**) and 6α-acetoxygedunin (**12**) and a small quantity of 6α-hydroxygedunin (**11**). Isolated compounds **1** and **12** exhibited physical and NMR and HRMS spectrometric data as described previously^24^ and [α]_D_^26^ −54.3 ± 0.9° (lit.^13^ [α]_D_^25^ −38.8° (CHCl_3_)) and +139.6 ± 2.7° (lit.^25^ [α]_D_^25^ + 141° (CHCl_3_)), respectively.

#### 6α-Hydroxygedunin (**11**)

White amorphous powder.^25^ ^1^H NMR (CDCl_3_, 300 MHz): δ 7.44 (2H, m, H23 and H21), 7.06 (1H, d, *J* = 10.1 Hz, H1), 6.36 (1H, m, H22), 5.94 (1H, d, *J* = 10.1 Hz, H2), 5.64 (1H, s, H17), 4.78 (1H, d, *J* = 2.3 Hz, H7), 4.29 (1H, dd, *J* = 11.9, 2.3 Hz, H6), 3.65 (1H, s, H15), 2.52 (1H, dd, *J* = 12.5, 5.9 Hz, H9), 2.25 (1H, d, *J* = 11.9 Hz, H5), 2.22 (3H, s, CH_3_CO), 2.00* (m, H11α), 1.85* (m, H11β), 1.74* (m, H12β), 1.62* (m, H12α), 1.41 (3H, s, H30), 1.32 (3H, s, H28), 1.27 (3H, s, H18), 1.22 (3H, s, H29), 1.18 (3H, s, H19). ^13^C NMR (CDCl_3_, 75 MHz): δ 205.5 (s, C3), 172.5 (s, CH_3_ CO), 167.3 (s, C16), 156.3 (d, C1), 143.2 (d, C23), 141.2 (d, C21), 126.7 (d, C2), 120.3 (s, C20), 109.8 (d, C22), 78.3 (d, C17), 77.2 (d, C7), 69.8 (s, C14), 68.5 (d, C6), 56.4 (d, C15), 49.6 (d, C5), 45.5 (s, C4), 43.1 (s, C8), 40.4 (s, C10), 38.8 (s, C13), 38.3 (d, C9), 31.9 (q, C28), 25.9 (t, C12), 21.6 (q, C29), 21.2 (q, CH_3_CO), 20.1 (q, C19), 18.6 (q, C30), 18.0 (q, C18), 15.0 (t, C11). Note: *chemical shift ascertained from the HSQC spectrum. HRMS (ESI): *m*/*z* 499.2333 [M + H]^+^, calculated for C_28_H_35_O_8_^+^ *m*/*z* 499.2326, Δ = 1.4 ppm.

### Hydride Reduction of Cedrolide (**1**)

#### Semisynthesis of Gedunin Derivatives **2**, **3**, and **16**

A stirred solution of cedrolide (1.0 g, 2.3 mmol) in MeOH (7 mL) was treated with NaBH_4_ (29 mg, 0.76 mmol) at 0 °C. After 30 min, the reaction mixture was allowed to warm to room temperature (r.t.) over 1 h. Analytical TLC provided evidence for conversion to reduction products. Next, addition of H_2_O (13 mL) and extraction with DCM (14 mL), drying of the organic phase with anhydrous Na_2_SO_4_ and evaporation, yielded a separable mixture of reduction products **2** (90 mg, 9.0%), **3** (483 mg, 48%) and **16** (12 mg, 1.2%) by preparative HPLC-PDA using as binary eluent system 9:1 ACN/H_2_O, a flow rate of 16 mL/min, and monitoring at a wavelength of 225 nm.

#### 7-Deacetylgedunin (**2**)

White amorphous powder. The 1D and 2D NMR data for the reduction product **2** are comparable to literature data.^28,37,42^ HRMS (ESI): *m*/*z* 441.2275 [M + H]^+^, calculated for C_26_H_33_O_6_^+^ *m*/*z* 441.2272, Δ = 0.7 ppm, [α]_D_^26^ + 55.7 ± 2.5° (lit. [α]_D_^28^ + 60.7°,^37^ [α]_D_^20^ +75° ^5,8^ ).

#### 7-Deacetyl-7-*epi*-gedunin (**3**)

White amorphous powder. ^1^H NMR (CDCl_3_, 300 MHz): δ 7.43 (2H, m, H21 and H23), 7.04 (1H, d, *J* = 10.2 Hz, H1), 6.34 (1H, m, H22), 5.87 (1H, d, *J* = 10.2 Hz, H2), 5.65 (1H, s, H17), 4.55 (1H, s, H15), 3.80 (1H, dd, *J* = 10.5, 4.8 Hz, H7), 1.99* (m, H9), 1.94* (m, H11α), 1.78* (m, H5), 1.76* (m, H6α), 1.76* (m, H11β), 1.75* (m, H12α), 1.52* (m, H6β), 1.52* (m, H12β), 1.21 (3H, s, H19), 1.20 (3H, s, H18), 1.18 (3H, s, H30), 1.17 (3H, s, H28), 1.10 (3H, s, H29). Note: *chemical shift ascertained from the HSQC spectrum. These data are comparable to partial ^1^H NMR data in the literature.^29^ ^13^C NMR (CDCl_3_, 75 MHz): δ 204.0 (s, C3), 168.2 (s, C16), 156.5 (d, C1), 143.1 (d, C21), 141.2 (d, C23), 126.2 (d, C2), 120.4 (s, C20), 109.9 (d, C22), 78.0 (d, C17), 77.2 (d, C7), 73.7 (s, C14), 56.9 (d, C15), 50.9 (d, C5), 44.4 (s, C4), 44.1 (s, C8), 43.5 (d, C9), 40.0 (s, C10), 39.3 (s, C13), 29.2 (t, C12), 27.5 (q, C28), 26.2 (t, C6), 21.4 (q, C29), 19.6 (q, C19), 19.0 (q, C18), 16.0 (t, C11), 13.6 (q, C30). HRMS (ESI): *m*/*z* 441.2287 [M + H]^+^, calculated for C_26_H_33_O_6_^+^ *m*/*z* 441.2272, Δ = 3.4 ppm, [α]_D_^26^ +105.9 ± 2.1° (lit.^29^ [α]_D_^20^ +48°).

#### 1,2-Dihydro-3α-hydroxy-3-deoxo-7-deacetyl-7-*epi*-gedunin **16**

White amorphous powder. ^1^H NMR (C_5_D_5_N, 300 MHz): δ 7.77 (1H, m, H23), 7.70 (1H, m, H21), 6.58 (1H, m, H22), 5.92 (1H, s, H17), 3.94 (1H, dd, *J* = 10.8, 4.5 Hz, H7), 3.50 (1H, t, *J* = 8.1 Hz, H3), 2.00–2.12 (1H, m, H6α), 1.90* (m, H2α and H2β), 1.78* (m, H6β), 1.77* (m, H5), 1.70* (m, H12β), 1.60* (m, H1α, H1β, and H11α), 1.37 (3H, s, H30), 1.36* (m, H12α), 1.32 (3H, s, H18), 1.27 (3H, s, H28), 1.11* (m, H11β), 1.10 (1H, m, H9), 1.05 (3H, s, H29), 0.97 (3H, s, H19). Note: *chemical shift ascertained from the HSQC spectrum. ^13^C NMR (C_5_D_5_N, 75 MHz): δ 169.4 (s, C16), 143.6 (d, C21), 142.0 (d, C23), 122.8 (s, C20), 110.9 (d, C22), 78.6 (d, C17), 77.9 (d, C7), 77.8 (d, C3), 74.3 (s, C14), 56.8 (d, C15), 53.6 (d, C9), 49.2 (d, C5), 44.4 (s, C8), 39.6 (s, C4), 39.3 (s, C13), 38.6 (t, C1), 37.9 (s, C10), 29.6 (t, C6), 28.5 (q, C28), 28.1 (t, C2), 26.7 (t, C12), 18.9 (q, C18), 16.5 (q, C19), 16.4 (q, C29), 16.2 (t, C11), 14.1 (q, C30). HRMS (ESI): *m*/*z* 445.2613 [M + H]^+^, calculated for C_26_H_37_O_6_^+^ *m*/*z* 445.2585, Δ = 6.5 ppm, [α]_D_^26^ + 75.4 ± 2.3°.

### General Procedures for Acylation of 7-Deacetylgedunin (**2**) and 7-Deacetyl-7-*epi*-gedunin (**3**)

#### Semisynthesis of 7-Acyl Gedunin Derivatives **4**–**9**

Acetic anhydride (10 μL, 110 μmol, 244 M%), butyric anhydride (15 μL, 90 μmol, 200 M%) or pentanoyl chloride (10.6 μL, 90 μmol, 200 M%) followed by DMAP (catalytic amount), were added to a stirred 0 °C suspension of **2** (20 mg, 45 μmol) in Et_3_N (5 mL) under an atmosphere of N_2_. The stirred mixture underwent reaction for 1 h at 0 °C and then was allowed to warm to r.t. over 23 h. After this time, the reaction was interrupted by the addition of deionized water. The resulting mixture was partitioned with CHCl_3_. The organic layer was separated and dried over Na_2_SO_4_. Evaporation of the dried organic layer yielded the crude product. Initially, purification of the crude product was carried out on a column (Ø × *h* = 1.2 × 10 cm) of flash silica gel 60 (0.040–0.063 mm mesh). The column was eluted with 3:2 EtOAc/hexanes. Pressure was applied to obtain a 5 cm/min flow rate. The rate of descent of the solvent was measured near the column head. Fractions containing product were combined based on TLC analysis. Subsequently, the resulting combined fraction underwent preparative HPLC-PDA using 7:3 ACN/H_2_O as the eluent system, a flow rate of 17 mL/min, and monitoring at a wavelength of 225 nm. This procedure provided pure product **4** (11.3 mg, 51.6%), **6** (7.5 mg, 32.8%), or **8** (6.4 mg, 26.9%), respectively. Similarly, acylation of **3** (50 mg, 110 μmol) with acetic anhydride (21 μL, 220 μmol, 200 M%), butyric anhydride (36 μL, 220 μmol, 200 M%) or pentanoyl chloride (26 μL, 220 μmol, 200 M%) and purification of the products utilizing the two-step procedure described above yielded product **5** (34.5 mg, 63.0%), **7** (23.4 mg, 40.4%), or **9** (17.6 mg, 29.6%), respectively.

#### Gedunin (**4**)

White amorphous powder. Our ^1^H and ^13^C NMR data are identical to those reported in the literature.^6^ HRMS (ESI): *m*/*z* 483.2391 [M + H]^+^, calculated for C_28_H_35_O_7_^+^ *m*/*z* 483.2377, Δ = 2.9 ppm, [α]_D_^26^ +82.6 ± 0.9° (lit. [α]_D_^20^ +44°,^4,5^ [α]_D_^27^ +42.8°^37^).

#### 7-*epi*-Gedunin (**5**)

White amorphous powder. ^1^H NMR (CDCl_3_, 300 MHz): δ 7.41 (2H, m, H21 and H23), 7.06 (1H, d, *J* = 10.2 Hz, H1), 6.34 (1H, m, H22), 5.88 (1H, d, *J* = 10.2 Hz, H2), 5.56 (1H, s, H17), 5.01 (1H, dd, *J* = 10.8, 4.5 Hz, H7), 3.70 (1H, s, H15), 2.14 (3H, s, CH_3_CO), 1.4–2.1 (m, H5, H6α, H6β, H9, H11α, H11β, H12α, and H12β), 1.25 (3H, s, H18), 1.20 (3H, s, H19), 1.16 (3H, s, H30), 1.14 (3H, s, H28), 1.09 (3H, s, H29). Comparable to partial ^1^H NMR data in the literature.^29^ ^13^C NMR (CDCl_3_, 75 MHz): δ 204.0 (s, C3), 170.3 (s, CH_3_ CO), 167.2 (s, C16), 156.6 (d, C1), 143.1 (d, C23), 141.1 (d, C21), 126.4 (d, C2), 120.2 (s, C20), 109.8 (d, C22), 78.1 (d, C17), 77.9 (d, C7), 70.5 (s, C14), 54.1 (d, C15), 51.2 (d, C5), 45.2 (d, C9), 44.6 (s, C8), 44.0 (s, C10), 39.7 (s, C4), 39.0 (s, C13), 29.3 (t, C6), 27.5 (q, C28), 25.6 (t, C12), 21.4 (q, CH_3_–CO), 21.1 (q, C19), 20.0 (q, C29), 20.0 (q, C30), 16.7 (t, C11), 14.1 (q, C18). HRMS (ESI): *m*/*z* 483.2406 [M + H]^+^, calculated for C_28_H_35_O_7_^+^ m/z 483.2377, Δ = 6.0 ppm, [α]_D_^26^ + 118.3 ± 0.4°.

#### 7-Deacetyl-7-butanoyloxygedunin (**6**)

White amorphous powder. ^1^H and ^13^C NMR data are identical to those in the literature.^32^ HRMS (ESI): *m*/*z* 511.2684 [M + H]^+^, calculated for C_30_H_39_O_7_^+^ *m*/*z* 511.2690, Δ = 1.2 ppm, [α]_D_^26^ +42.3 ± 0.8° (lit.^32^ [α]_D_^27^ + 42).

#### 7-Deacetyl-7-butanoyl-7-*epi*-gedunin (**7**)

White amorphous powder. ^1^H NMR (CDCl_3_, 300 MHz): δ 7.41 (2H, m, H21 and H23), 7.06 (1H, d, *J* = 10.2 Hz, H1), 6.34 (1H, t, *J* = 1.4 Hz, H22), 5.88 (1H, d, *J* = 10.2 Hz, H2), 5.56 (1H, s, H17), 5.00 (1H, dd, *J* = 10.8, 4.5 Hz, H7), 3.69 (1H, s, H15), 2.30–2.43 (2H, m, CH_3_CH_2_CH_2_CO), 2.00* (m, H9), 1.97* (m, H11α), 1.96* (m, H6α), 1.87* (m, H5), 1.84* (m, H11β), 1.80* (m, H12β), 1.71* (m, CH_3_CH_2_CH_2_CO), 1.66* (m, H6β), 1.51* (m, H12α), 1.25 (3H, s, H18), 1.20 (3H, s, H19), 1.17 (3H, s, H30), 1.16 (3H, s, H28), 1.09 (3H, s, H29), 1.00 (3H, *t*, *J* = 7.0 Hz, CH_3_CH_2_CH_2_CO). Note: *chemical shift ascertained from the HSQC spectrum. ^13^C NMR (CDCl_3_, 75 MHz): δ 204.0 (s, C3), 172.9 (s, CH_3_CH_2_CH_2_ CO), 167.2 (s, C16), 156.5 (d, C1), 143.1 (d, C23), 141.1 (d, C21), 126.4 (d, C2), 120.3 (s, C20), 109.8 (d, C22), 78.1 (d, C17), 77.8 (d, C7), 70.8 (s, C14), 54.4 (d, C15), 51.0 (d, C5), 45.0 (d, C9), 44.6 (s, C4), 43.9 (s, C8), 39.7 (s, C10), 39.1 (s, C13), 36.6 (t, CH_3_CH_2_ CH_2_CO), 28.9 (t, C12), 27.5 (q, C28), 25.5 (t, C6), 21.1 (q, C29), 19.9 (q, C19), 19.9 (q, C18), 18.2 (t, CH_3_ CH_2_CH_2_CO), 16.6 (t, C11), 14.2 (q, C30), 13.8 (q, CH_3_CH_2_CH_2_CO). HRMS (ESI): *m*/*z* 511.2704 [M + H]^+^, calculated for C_30_H_39_O_7_^+^ *m*/*z* 511.2690, Δ = 2.7 ppm, [α]_D_^26^ + 95.6 ± 0.5°.

#### 7-Deacetyl-7-pentanoylgedunin (**8**)

White amorphous powder. ^1^H NMR (CDCl_3_, 300 MHz): δ 7.43 (1H, m, H23), 7.42 (1H, m, H21), 7.12 (1H, d, *J* = 10.2 Hz, H1), 6.35 (1H, t, *J* = 1.2 Hz, H22), 5.88 (1H, d, *J* = 10.2 Hz, H2), 5.62 (1H, s, H17), 4.57 (1H, d, *J* = 3.0 Hz, H7), 3.53 (1H, s, H15), 2.50 (1H, dd, *J* = 12.3, 6.0 Hz, H9), 2.3–2.4 (2H, m, CH_3_CH_2_CH_2_CH_2_CO), 2.17 (1H, dd, *J* = 13.1, 2.6 Hz, H5), 2.10* (m, H_α_-11), 1.99* (m, H6α), 1.88* (m, H6β), 1.84* (m, H11β), 1.71* (m, H12β), 1.61* (2H, m, CH_3_CH_2_CH_2_CH_2_CO), 1.50* (m, H12α), 1.31* (m, CH_3_CH_2_CH_2_CH_2_CO), 1.25 (3H, s, H18), 1.23 (3H, s, H19), 1.17 (3H, s, H30), 1.08 (3H, s, H29), 1.06 (3H, s, H28), 0.91 (3H, t, *J* = 7.2 Hz, CH_3_CH_2_CH_2_CH_2_CO). Note: *chemical shift ascertained from the HSQC spectrum. ^13^C NMR (CDCl_3_, 75 MHz): δ 204.1 (s, C3), 172.8 (s, CH_3_CH_2_CH_2_CH_2_ CO), 167.4 (s, C16), 157.1 (d, C1), 143.1 (d, C21), 141.2 (d, C23), 126.0 (d, C2), 120.4 (s, C20), 109.9 (d, C22), 78.2 (d, C17), 72.8 (d, C7), 69.7 (s, C14), 57.0 (d, C15), 46.0 (d, C5), 44.1 (s, C4), 42,6 (s, C8), 40.1 (s, C10), 39.5 (d, C9), 38.7 (s, C13), 34.2 (t, CH_3_CH_2_CH_2_ CH_2_CO), 27.2 (C28), 26.9 (t, CH_3_CH_2_ CH_2_CH_2_CO), 25.9 (t, C12), 23.3 (t, C6), 22.3 (t, CH_3_ CH_2_CH_2_CH_2_CO), 21.2 (q, C29), 19.8 (q, C19), 18.4 (q, C30), 17.7 (q, C18), 15.0 (t, C11), 13.7 (q, CH_3_CH_2_CH_2_CH_2_CO). HRMS (ESI): *m*/*z* 525.2874 [M + H]^+^, calculated for C_31_H_41_O_7_^+^ *m*/*z* 525.2847, Δ = 5.2 ppm, [α]_D_^26^ + 25.0 ± 2.4°.

#### 7-Deacetyl-7-pentanoyl-7-*epi*-gedunin (**9**)

White amorphous powder. ^1^H NMR (CDCl_3_, 300 MHz): δ 7.41 (2H, m, H21 and H23), 7.07 (1H, d, *J* = 10.2 Hz, H1), 6.34 (1H, t, *J* = 1.2 Hz, H22), 5.88 (1H, d, *J* = 10.2 Hz, H2), 5.56 (1H, s, H17), 5.00 (1H, dd, *J* = 10.7, 4.4 Hz, H7), 3.69 (1H, s, H15), 2.30–2.35 (2H, m, CH_3_CH_2_CH_2_CH_2_CO), 2.0* (m, H5), 1.94* (m, H6α and H11α), 1.88* (m, H9), 1.86* (m, H11β), 1.80* (m, H12β), 1.65* (m, H6β), 1.61* (m, CH_3_CH_2_CH_2_CH_2_CO), 1.50* (m, H12α), 1.31* (m, CH_3_CH_2_CH_2_CH_2_CO), 1.25 (3H, s, H18), 1.20 (3H, s, H19), 1.17 (6H, s, H28 and H30), 1.09 (3H, s, H29), 0.98 (3H, t, *J* = 7.0 Hz, CH_3_CH_2_CH_2_CH_2_CO). Note: *chemical shift ascertained from the HSQC spectrum. ^13^C NMR (CDCl_3_, 75 MHz): δ 204.0 (s, C3), 173.1 (s, CH_3_CH_2_CH_2_CH_2_ CO), 167.2 (s, C16), 156.5 (d, C1), 143.1 (d, C21), 141.1 (d, C23), 126.4 (d, C2), 120.3 (s, C20), 109.8 (d, C22), 78.1 (d, C17), 77.8 (d, C7), 70.9 (s, C14), 54.4 (d, C15), 51.0 (d, C9), 45.0 (d, C5), 44.6 (s, C4), 43.9 (s, C8), 39.7 (s, C10), 39.1 (s, C13), 34.4 (t, CH_3_CH_2_CH_2_ CH_2_CO), 28.9 (t, C12), 27.5 (C28), 26.7 (t, CH_3_CH_2_ CH_2_CH_2_CO), 25.5 (t, C6), 22.3 (t, CH_3_ CH_2_CH_2_CH_2_CO), 21.1 (q, C29), 19.9 (q, C18 and C19), 16.6 (t, C11), 14.2 (q, C30), 13.7 (q, CH_3_CH_2_CH_2_CH_2_CO). HRMS (ESI): *m*/*z* 525.2842 [M + H]^+^, calculated for C_31_H_41_O_7_^+^ *m*/*z* 525.2847, Δ = 0.9 ppm, [α]_D_^26^ + 56.7 ± 2.4°.

#### Semisynthesis of 6α-Hydroxy-7-deacetylgedunin (**10**)

Hydrolysis of 6α-acetoxygedunin (**12**) was performed via a modified literature procedure.^25^ Thus, compound **12** (1.0 g) dissolved in MeOH (70 mL) was hydrolyzed by addition of 10% aqueous KOH (10 mL) and stirring under reflux for 40 min at 70–75 °C and then neutralization with 0.1 N HCl. The neutralized reaction mixture was exhaustively extracted with DCM. The combined DCM extracts were dried over anhydrous Na_2_SO_4_ and rotary evaporated to provide crude deacetylation product **10**. The crude product underwent flash chromatography (silica gel 60, 40–63 mm mesh, Ø × *h* = 2.6 × 17 cm, eluents: 7:3 EtOAc/hexanes) using pressure to provide a flow rate of 5 cm/min at the column head. This procedure provided pure **10** whose NMR and HRMS spectrometric data (not shown) were identical to those published in previous work.^24^ This compound exhibited [α]_D_^26^ + 55.6 ± 2.3°. No optical rotation data are provided for **10** in previous reports.^24,25^

### General Procedure for Acylation of 6α-Hydroxy-7-deacetylgedunin (**10**)

#### Semisynthesis of 6α-Acyl Derivatives **13**–**15**

The procedure involved the addition of acylating agent (butyric anhydride, benzoyl chloride or heptanoyl chloride; 200 M%) and the catalysts Et_3_N (17 μL, 0.124 mmol, 114M%) and DMAP (catalytic amount) to a stirred 0 °C solution of compound **10** (50 mg, 0.109 mmol) in DCM (5 mL) under an atmosphere of Ar. The reaction was stirred at 0 °C under an atmosphere of Ar for 1 h and then allowed to come to r.t. over 23 h. Next, the reaction was quenched by the addition of DCM (5.0 mL) and a saturated solution of NH_4_Cl. The phases were separated. The organic layer was dried over Na_2_SO_4_ and then evaporated to yield the crude product. Next, the crude product was chromatographed on a column of flash silica gel 60 (40–63 μm mesh, Merck, Ø × *h* = 1.2 × 16 cm, eluent system: 1:1 hexanes/EtOAc), using pressure to maintain a flow of 5 cm/min at the column head. This procedure provided product (compound **13**, **14**, or **15**) that was further purified by preparative HPLC-PDA using isocratic ACN/H_2_O (75:25), a flow rate of 17 mL/min, and monitoring at a wavelength of 225 nm.

#### 6α-Butanoyloxy-7-deacetylgedunin (**13**)

White amorphous powder. ^1^H NMR (CDCl_3_, 300 MHz): δ 7.41 (2H, m, H21 and H23) 7.06 (1H, d, *J* = 10.2 Hz, H1), 6.35 (1H, m, H22), 5.91 (1H, d, *J* = 10.2 Hz, H2), 5.61 (1H, s, H17), 5.32 (1H, dd, *J* = 12.3, 1.8 Hz, H6), 3.89 (1H, s, H15), 3.47 (1H, s, H7), 2.75 (1H, d, *J* = 12.3 Hz, H5), 2.59 (1H, dd, *J* = 12.6, 6.0 Hz, H9), 2.33 (3H, m*,* 7-OH and CH_3_CH_2_CH_2_CO), 1.95* (m, H11α), 1.84* (m, H12β), 1.69* (m, H11β), 1.68* (m, CH_3_CH_2_CH_2_CO), 1.53* (m, H12α), 1.28 (3H, s, H28), 1.26 (3H, s, H29), 1.20 (3H, s, H18), 1.19 (3H, s, H30), 1.17 (3H, s, H19), 0.98 (3H, t*, J* = 7.4 Hz, CH_3_CH_2_CH_2_CO). Note: *chemical shift ascertained from the HSQC spectrum. ^13^C NMR (CDCl_3_, 75 MHz): δ 204.6 (s, C3), 172.5 (s, CH_3_CH_2_CH_2_ CO), 168.2 (s, C16), 156.8 (d, C1), 143.0 (d, C23), 141.2 (d, C21), 126.3 (d, C2), 120.5 (C20), 109.9 (d, C22), 78.5 (d, C17), 71.9 (d, C6) 71.9 (d, C7), 69.8 (s, C14), 58.0 (d, C15), 46.6 (d, C5), 44.9 (s, C10), 43.4 (s, C8), 40.7 (s, C4), 38.3 (s, C13), 36.7 (d, C9), 36.6 (t, CH_3_CH_2_ CH_2_CO), 31.7 (q, C28), 26.1 (t, C12), 21.5 (q, C29), 20.6 (q, C19), 18.2 (t, CH_3_ CH_2_CH_2_CO), 17.8 (q, C18), 17.8 (q, C30), 15.0 (t, C11), 13.7 (q, CH_3_CH_2_CH_2_CO). HRMS (ESI): *m*/*z* 527.2640 [M + H]^+^, calculated for C_30_H_39_O_8_^+^ *m*/*z* 527.2639, Δ = 0.0 ppm, [α]_D_^26^ + 10.7 ± 1.9°.

##### 6α-Benzoyloxy-7-deacetylgedunin (**14**)

White amorphous powder, ^1^H NMR (CDCl_3_, 300 MHz): δ 8.13 (2H, *m*, PhH_*ortho*_), 7.63 (1H, *m*, PhH_*para*_), 7.48 (2H, *m*, PhH_*meta*_), 7.40 (2H, *m*, H21 and H23), 7.08 (1H, *d*, *J* = 10.2 Hz, H1), 6.33 (1H, *t*, *J* = 1.2 Hz, H22), 5.92 (1H, *d*, *J* = 10.2 Hz, H2), 5.60 (1H, *dd*, *J* ∼ 12.4, 2.0 Hz, H6), 5.58 (1H, *s*, H17), 3.79 (1H, *s*, H15), 3.55 (1H, *d*, *J* = 2.0 Hz, H7), 2.90 (1H, *d*, *J* = 12.4 Hz, H5), 2.63 (1H, *dd*, *J* = 12.3, 5.7 Hz, H9), 2.00* (*m*, H11α), 1.85* (*m*, H11β), 1.74* (*m*, H12β), 1.62* (*m*, H12α), 1.33 (3H, *s*, H30), 1.25 (3H, *s*, H28), 1.23 (3H, *s*, H18), 1.22 (3H, *s*, H29), 1.16 (3H, *s*, H19). Note: *chemical shift ascertained from the HSQC spectrum. ^13^C NMR (CDCl_3_, 75 MHz): δ 204.6 (*s*, C3), 168.1 (*s*, PhCO) 165.4 (*s*, C16), 156.8 (*d*, C1), 143.0 (*d*, C21), 141.2 (*d*, C23), 133.9 (*d*, C_*para*_-Ph), 130.1 (C_*ipso*_-Ph), 129.7 (*d*, C_*ortho*_-Ph), 129.3 (*d*, C_*meta*_-Ph), 126.4 (*d*, C2), 120.5 (*s*, C20), 109.9 (*d*, C22), 78.5 (*d*, C17), 72.7 (*d*, C6), 72.1 (*d*, C7), 69.8 (*s*, C14), 57.9 (*d*, C15), 46.9 (*d*, C5), 45.0 (*s*, C4), 43.6 (*s*, C8), 40.8 (*s*, C10), 38.4 (*s*, C13), 36.7 (*d*, C9), 31.9 (*q*, C28), 26.2 (*t*, C12), 21.6 (*q*, C19), 20.7 (*q*, C29), 17.9 (*q*, C18), 17.8 (*q*, C30), 15.1 (*t*, C11). HRMS (ESI): *m*/*z* 561.2500 [M + H]^+^, calculated for C_33_H_37_O_8_^+^ *m*/*z* 561.2483, Δ = 3.0 ppm, [α]_D_^26^ +137.8 ± 0.9°.

##### 6α-Heptanoyloxy-7-deacetylgedunin (**15**)

White amorphous powder. ^1^H NMR (CDCl_3_, 300 MHz): δ 7.40–7.45 (2H, m, H21 and H23), 7.06 (1H, d, *J* = 10.2 Hz, H1), 6.35 (1H, dd, *J* = 1.5, 0.9 Hz, H22), 5.92 (1H, d, *J* = 10.2 Hz, H2), 5.61 (1H, s, H17), 5.33 (1H, dd, *J* = 12.3, 2.1 Hz, H6), 3.88 (1H, s, H15), 3.46 (1H, d, *J* = 1.8 Hz, H7), 2.74 (1H, d, *J* = 12.3 Hz, H5), 2.59 (1H, dd, *J* = 12.5, 5.9 Hz, H9), 2.32–2.45 (2H, m, CH_3_CH_2_CH_2_CH_2_CH_2_CH_2_CO), 1.96* (m, H11α), 1.86* (m, H11β), 1.75* (m, H12β), 1.65* (m, CH_3_CH_2_CH_2_CH_2_CH_2_CH_2_CO), 1.56* (m, H12α), 1.31* (6H, m, CH_3_CH_2_CH_2_CH_2_CH_2_CH_2_CO), 1.27 (3H, *s*, H28), 1.25 (3H, s, H18), 1.20 (3H, s, H19), 1.19 (3H, s, H30), 1.17 (3H, s, H29), 0.91 (3H, m, CH_3_CH_2_CH_2_CH_2_CH_2_CH_2_CO). Note: *chemical shift ascertained from the HSQC spectrum. ^13^C NMR (CDCl_3_, 75 MHz): δ 204.5 (s, C3), 172.6 (s, CH_3_CH_2_CH_2_CH_2_CH_2_CH_2_ CO), 168.1 (s, C16), 156.7 (d, C1), 143.0 (d, C23), 141.2 (d, C21), 126.3 (d, C2), 120.5 (s, C20), 109.9 (d, C22), 78.4 (d, C17), 72.0 (d, C7), 71.9 (d, C6), 69.7 (s, C14), 58.0 (d, C15), 46.6 (d, C5), 44.9 (s, C10), 43.4 (s, C8), 40.7 (s, C4), 38.3 (s, C13), 36.7 (d, C9), 34.8 (t, CH_3_CH_2_CH_2_CH_2_CH_2_ CH_2_CO), 31.7 (q, C28), 31.4 (t, CH_3_CH_2_CH_2_ CH_2_CH_2_CH_2_CO), 28.8 (t, CH_3_CH_2_ CH_2_CH_2_CH_2_CH_2_CO), 26.1 (t, C12), 24.7 (t, CH_3_CH_2_CH_2_CH_2_ CH_2_CH_2_CO), 22.5 (t, CH_3_ CH_2_CH_2_CH_2_CH_2_CH_2_CO), 21.5 (q, C29), 20.6 (q, C19), 17.9 (q, C30), 17.7 (q, C18), 15.0 (t, C11), 14.0 (q, CH_3_CH_2_CH_2_CH_2_CH_2_CH_2_CO). HRMS (ESI): *m*/*z* 569.3122 [M + H]^+^, calculated for C_33_H_45_O_8_^+^ *m*/*z* 569.3109, Δ = 2.3 ppm, [α]_D_^26^ + 102.7 ± 1.5°.

### In Vitro Culture of *Plasmodium falciparum* and Susceptibility Assay (Microtest)

In vitro culture of the K1 strain of *P. falciparum* (MRA-159, MR4, ATCC, Manassas, Virginia, USA) proceeded following an established method^43^ with modifications.^44^ Briefly, this method involved the growth of parasites in human type A+ red blood cells and RPMI 1640 medium supplemented with 10% type A+ human plasma at 37 °C under an atmosphere of 5% O_2_, 5% CO_2_, and 90% N_2_.

As initial condition for the susceptibility assay, cultures were synchronized by treatment with 5% d-sorbitol, which provided young trophozoites (ring stages).^45^ Performance of the microtest followed a previously described procedure^46^ with modifications.^44^ Compounds were dissolved in DMSO with the aid of an ultrasound bath to provided stock solutions. Stock solutions then underwent sequential dilution in culture medium (RPMI 1640) which resulted in seven diluted samples with concentrations in the range 1.56–100 μg/mL (well concentrations) and final (well) DMSO concentrations of ≤1%. These diluted samples were evaluated in duplicate in a 96-well test plate. A suspension of parasitized red blood cells (pRBCs) at 2% hematocrit and 1% parasitemia was added to each well. Microplate incubation proceeded for 48 h at 37 °C under the same atmosphere used for parasite culture in two independent experiments conducted on different days. Control wells contained chloroquine (0.003–2.5 μg/mL) and quinine (0.003–2.5 μg/mL). Six (6) control (blank) wells were prepared with pRBCs suspension. Six cosolvent control wells were prepared with 1% DMSO and pRBCs suspension. An average of these 12 controls defined drug-free (untreated) growth. After the incubation period, thin blood smears of the contents of each well were prepared on microscope slides. Optical microscopy was used to analyze the blood smears and establish the parasitemia of each well. Interpolation of the nonlinear curve using GraphPad Prism permitted the calculation of estimates of the sample concentrations able to inhibit 50% of parasite growth (IC_50_) compared to drug-free controls. In general, the IC_50_ values represent the results from two independent experiments performed on different days with a confidence interval of 95%.

### Cytotoxicity/Cell Viability Assay

Medical Research Council cell strain 5 (MRC-5) human fetal fibroblasts were cultivated in Dulbecco′s modified Eagle’s medium (DMEM) supplemented with 10% bovine fetal serum and 1% antibiotic at 37 °C under an atmosphere of 5% CO_2_. Cell viability assessment relied on the Alamar blue method.^47^ Each substance was initially diluted in DMSO (well concentration did not exceed 0.2% DMSO) and the resulting solution was further diluted with culture medium and tested at a concentration of 50 μg/mL for 48 h. Doxorubicin at concentrations of 0.312, 0.625, 1.25, 2.50, 5.00, 10.0, and 20.0 μM was used as a positive control for cell death. The negative control wells received DMSO (sample diluent) at 0.1%. In preparation for the assay, this method involved plating 0.5 × 10^4^ cells/well on 96 well test plates and incubating for 24 h under the same conditions used for cell culture. This initial 24 h period was necessary for cell adhesion. The assay consisted of triplicate addition of diluted samples and controls to wells followed by 48 h of incubation. For quantification of viable cells, 10 μL of 0.4% Alamar blue (Sigma-Aldrich)—which works out to an in-well concentration of 0.02%—were added to each well 3 h before the end of the treatment. Alamar blue was allowed to react for 3 h followed by quantification of the optical density (OD) of each well at an excitation wavelength of 465 nm and emission wavelength of 540 nm using an ELISA plate reader (DTX-800, Beckman Coulter). Comparison of individual sample viability OD to that of controls (blanks) afforded the cell viability. Interpolation of the nonlinear curve using GraphPad Prism software permitted the calculation of estimates of the sample IC_50_ values compared to drug-free controls.

### Selectivity Index

Calculation of the selectivity index (SI) using the following formula afforded the relative toxicity of each sample to human fibroblasts and to *P. falciparum* blood stages: SI = IC_50_ (human fibroblasts) ÷ IC_50_ (*P. falciparum*).

## Supplementary Material



## Notes

Additional experimental details, LC-PDA-HRMS and NMR spectra for all compounds; and primary (FID) 1D and 2D NMR data for all compounds (PDF)

## 1

Universidade Estadual do Amazonas, Centro de Estudos Superiores de Parintins, Estrada Odovaldo Novo (Estrada do Aeroporto), s/n, CEP 69152–470, Parintins, Amazonas, Brazil
