# Supplementary material for: Chemical and Antiplasmodial Investigations on Carapa-Derived Gedunin Derivatives and Semisynthetic 6- and 7‑Substituted Gedunin Analogues from the Brazilian Amazon
Source: ACS Omega. 2025 Sep 29;10(40):46832–43. doi: 10.1021/acsomega.5c04736 (PMC12529149; doi:10.1021/acsomega.5c04736)
Supplement: Supplementary file 1 [file ao5c04736_si_001.pdf]

Supporting Information: Additional experimental details, LC-PDA-HRMS and NMR spectra for all compounds.

Chemical and Antiplasmodial Investigations on *Carapa*-derived Gedunin Derivatives and Semisynthetic 6- and 7-Substituted Gedunin Analogues from the Brazilian Amazon

Tiago Barbosa Pereira,[a,b,1] Laís Garcia Jordão,[c] Djalma da Silva Pereira,[a,1] Gustavo Souza dos Santos,[b] Daniel Soares dos Santos,[b] Roberto Figliuolo,[d] Jaqueline Siqueira da Costa,[e] Leilane de Sousa Mendonça,[f] Emersom Silva Lima,[f] Marne Carvalho de Vasconcellos[f] and Adrian Martin Pohlit[a,b,c,*]

[a]*Universidade Federal do Amazonas, Instituto de Ciências Exatas e da Terra, Programa de Pós-graduação em Química, Avenida General Rodrigo Otávio, 6200, Campus Universitário Senador Arthur Virgílio Filho, Setor Norte, Bloco 3 (ICE), Coroado I, CEP 69077-000, Manaus, Amazonas, Brazil.*

[b]*Instituto Nacional de Pesquisas da Amazônia, Coordenação de Tecnologia e Inovação, Laboratório de Princípios Ativos da Amazônia, Avenida André Araújo, 2936, Petrópolis, CEP 69067-375, Manaus, Amazonas, Brazil.*

[c]*Universidade Federal do Amazonas, Instituto de Ciências Biológicas, Programa de Pós-graduação em Biotecnologia, Avenida General Rodrigo Octávio Jordão Ramos, 6200, Campus Universitário Senador Arthur Virgílio Filho, Setor Sul, Bloco M, Coroado I, CEP 69077-000, Manaus, Amazonas, Brazil.*

[1] Present address: Universidade Estadual do Amazonas, Centro de Estudos Superiores de Parintins, Estrada Odovaldo Novo (Estrada do Aeroporto), s/n, CEP 69152-470, Parintins, Amazonas, Brazil.

[d]*Instituto Nacional de Pesquisas da Amazônia, Coordenação de Tecnologia e Inovação, Laboratório de Extração Semi-industrial, Avenida André Araújo, 2936, Petrópolis, CEP 69067-375, Manaus, Amazonas, Brazil.*

[e]*Instituto Nacional de Pesquisas da Amazônia, Coordenação de Sociedade, Ambiente e Saúde, Laboratório de Cultivo de Plasmodium, Avenida André Araújo, 2936, Petrópolis, CEP 69067-375, Manaus, Amazonas, Brazil.*

[f]*Universidade Federal do Amazonas, Faculdade de Ciências Farmacêuticas, Progama de Pós-graduação em Inovação Farmacêutica, Avenida General Rodrigo Octávio, 6200, Campus Universitário Senador Arthur Virgílio Filho, Mini-campus, Coroado I, CEP 69077-000, Manaus, Amazonas, Brazil.*

*Adrian Martin Pohlit - *adrian.pohlit@inpa.gov.br*

Contents

Summary

Figure S1. Structures of gedunin derivatives isolated from *Carapa* spp. (**1**, **11**, **12**) and semisynthetic compounds gedunin (**4**) and derivatives (**2**, **3**, **5–10**, **13-16**).

Limonoid isolation method 1

Limonoid isolation method 2

Limonoid isolation method 3

7-Deacetylgedunin (2):

^{1}H and ^{13}C NMR chemical shift data for 7-deacetylgedunin (**2**).

Figure S2: UFLC-PDA-ESI (+)-HRMS data for 7-deacetylgedunin (**2**).

Figure S3: ^{1}H NMR ($CDCl_3$, 300 MHz) spectrum of 7-deacetylgedunin (**2**).

Figure S4: ^{13}C NMR ($CDCl_3$, 75 MHz) spectrum of 7-deacetylgedunin (**2**).

Figure S5: DEPT 135º ($CDCl_3$, 75 MHz) spectrum of 7-deacetylgedunin (**2**).

Figure S6: DEPT 90º ($CDCl_3$, 75 MHz) spectrum of 7-deacetylgedunin (**2**).

Figure S7: COSY ($CDCl_3$, 300 MHz) spectrum of 7-deacetylgedunin (**2**).

Figure S8: HSQC ($CDCl_3$, 300; 75 MHz) spectrum of 7-deacetylgedunin (**2**).

Figure S9: HMBC ($CDCl_3$, 300; 75 MHz) spectrum of 7-deacetylgedunin (**2**).

7-Deacetyl-7-*epi*-gedunin (3):

Figure S10: UFLC-PDA-ESI-(+)-HRMS data for 7-deacetyl-7-*epi*-gedunin (**3**).

Figure S11: ^{1}H NMR ($CDCl_3$, 300 MHz) spectrum of 7-deacetyl-7-*epi*-gedunin (**3**).

Figure S12:^{13}C NMR ($CDCl_3$, 75 MHz) spectrum of 7-deacetyl-7-*epi*-gedunin (**3**).

Figure S13: DEPT 135º ($CDCl_3$, 75 MHz) spectrum of 7-deacetyl-7-*epi*-gedunin (**3**).

Figure S14: DEPT 90º ($CDCl_3$, 75 MHz) spectrum of 7-deacetyl-7-*epi*-gedunin (**3**).

Figure S15: COSY ($CDCl_3$, 300 MHz) spectrum of 7-deacetyl-7-*epi*-gedunin (**3**).

Figure S16: HSQC ($CDCl_3$, 300; 75 MHz) spectrum of 7-deacetyl-7-*epi*-gedunin (**3**).

Figure S17: HMBC ($CDCl_3$, 300; 75 MHz) spectrum of 7-deacetyl-7-*epi*-gedunin (**3**).

Gedunin (4):

^{1}H and ^{13}C NMR chemical shift data for gedunin (**4**).

Figure S18: UFLC-PDA-ESI-(+)-HRMS data for gedunin (**4**).

Figure S19: ^{1}H NMR ($CDCl_3$, 300 MHz) spectrum of gedunin (**4**).

Figure S20: ^{13}C NMR ($CDCl_3$, 75 MHz) spectrum of gedunin (**4**).

Figure S21: DEPT 135º ($CDCl_3$, 75 MHz) spectrum gedunin (**4**).

Figure S22: DEPT 90º ($CDCl_3$, 75 MHz) spectrum of gedunin (**4**).

Figure S23: COSY ($CDCl_3$, 300 MHz) spectrum of gedunin (**4**).

Figure S24: HSQC ($CDCl_3$, 300; 75 MHz) spectrum of gedunin (**4**).

Figure S25: HMBC ($CDCl_3$, 300; 75 MHz) spectrum of gedunin (**4**).

7-*Epi*-gedunin (5):

Figure S26: UFLC-PDA-ESI-(+)-HRMS data for 7-*epi*-gedunin (**5**).

Figure S27: ^{1}H NMR ($CDCl_3$, 300 MHz) spectrum of 7-*epi*-gedunin (**5**).

Figure S28: ^{13}C NMR ($CDCl_3$, 75 MHz) spectrum of 7-*epi*-gedunin (**5**).

Figure S29: DEPT 135º ($CDCl_3$, 75 MHz) spectrum of 7-*epi*-gedunin (**5**).

Figure S30: DEPT 90º ($CDCl_3$, 75 MHz) spectrum of 7-*epi*-gedunin (**5**).

Figure S31: COSY ($CDCl_3$, 300 MHz) spectrum of 7-*epi*-gedunin (**5**).

Figure S32: HSQC ($CDCl_3$, 300; 75 MHz) spectrum of 7-*epi*-gedunin (**5**).

Figure S33: HMBC ($CDCl_3$, 300; 75 MHz) spectrum of 7-*epi*-gedunin (**5**).

7-Deacetyl-7α-butanoyloxygedunin (6):

^{1}H and ^{13}C NMR chemical shift data for 7-deacetyl-7α-butanoyloxygedunin (**6**).

Figure S34: UFLC-PDA-ESI-(+)-HRMS data for 7α-butanoyloxygedunin **6**.

Figure S35: ^{1}H NMR ($CDCl_3$, 300 MHz) spectrum of 7α-butanoyloxygedunin **6**.

Figure S36: ^{13}C NMR ($CDCl_3$, 75 MHz) spectrum of 7α-butanoyloxygedunin **6**.

Figure S37: DEPT 135º ($CDCl_3$, 75 MHz) spectrum of 7α-butanoyloxygedunin **6**.

Figure S38: DEPT 90º ($CDCl_3$, 75 MHz) spectrum of 7α-butanoyloxygedunin **6**.

Figure S39: COSY ($CDCl_3$, 300 MHz) spectrum of 7α-butanoyloxygedunin **6**.

Figure S40: HSQC ($CDCl_3$, 300; 75 MHz) spectrum of 7α-butanoyloxygedunin **6**.

Figure S41: HMBC ($CDCl_3$, 300; 75 MHz) spectrum of 7α-butanoyloxygedunin **6**.

7-Deacetyl-7β-butanoyloxygedunin (7):

Figure S42: UFLC-PDA-ESI-(+)-HRMS data for new 7β-butanoyloxygedunin **7**.

Figure S43: ^{1}H NMR ($CDCl_3$, 300 MHz) spectrum of new 7β-butanoyloxygedunin **7**.

Figure S44: ^{13}C NMR ($CDCl_3$, 75 MHz) spectrum of new 7β-butanoyloxygedunin **7**.

Figure S45: DEPT 135º ($CDCl_3$, 75 MHz) spectrum of new 7β-butanoyloxygedunin **7**.

Figure S46: DEPT 90º ($CDCl_3$, 75 MHz) spectrum of new 7β-butanoyloxygedunin **7**.

Figure S47: COSY ($CDCl_3$, 300 MHz) spectrum of new 7β-butanoyloxygedunin **7**.

Figure S48: HSQC ($CDCl_3$, 300; 75 MHz) spectrum of new 7β-butanoyloxygedunin **7**.

Figure S49: HMBC ($CDCl_3$, 300; 75 MHz) spectrum of new 7β-butanoyloxygedunin **7**.

7-Deacetyl-7α-pentanoyloxygedunin (8):

Figure S50: UFLC-PDA-ESI-(+)-HRMS data for new 7α-pentanoyloxygedunin **8**.

Figure S51: ^{1}H NMR ($CDCl_3$, 300 MHz) spectrum of new 7α-pentanoyloxygedunin **8**.

Figure S52: ^{13}C NMR ($CDCl_3$, 75 MHz) spectrum of new 7α-pentanoyloxygedunin **8**.

Figure S53: DEPT 135º ($CDCl_3$, 75 MHz) spectrum of new 7α-pentanoyloxygedunin **8**.

Figure S54: DEPT 90º ($CDCl_3$, 75 MHz) spectrum of new 7α-pentanoyloxygedunin **8**.

Figure S55: COSY ($CDCl_3$, 300 MHz) spectrum of new 7α-pentanoyloxygedunin **8**.

Figure S56: HSQC ($CDCl_3$, 300; 75 MHz) spectrum of new 7α-pentanoyloxygedunin **8**.

Figure S57: HMBC ($CDCl_3$, 300; 75 MHz) spectrum of new 7α-pentanoyloxygedunin **8**.

7-Deacetyl-7β-pentanoyloxygedunin (9):

Figure S58: UFLC-PDA-ESI-(+)-HRMS data for new 7β-pentanoyloxygedunin **9**.

Figure S59: ^{1}H NMR ($CDCl_3$, 300 MHz) spectrum of new 7β-pentanoyloxygedunin **9**.

Figure S60: ^{13}C NMR ($CDCl_3$, 75 MHz) spectrum of new 7β-pentanoyloxygedunin **9**.

Figure S61: DEPT 135º ($CDCl_3$, 75 MHz) spectrum of new 7β-pentanoyloxygedunin **9**.

Figure S62: DEPT 90º ($CDCl_3$, 75 MHz) spectrum of new 7β-pentanoyloxygedunin **9**.

Figure S63: COSY ($CDCl_3$, 300 MHz) spectrum of new 7β-pentanoyloxygedunin **9**.

Figure S64: HSQC ($CDCl_3$, 300; 75 MHz) spectrum of new 7β-pentanoyloxygedunin **9**.

Figure S65 HMBC ($CDCl_3$, 300; 75 MHz) spectrum of new 7β-pentanoyloxygedunin **9**.

6α-Hydroxygedunin (11):

Figure S66: UFLC-PDA-ESI-(+)-HRMS data for 6α-hydroxygedunin (**11**).

Figure S67: ^{1}H NMR ($CDCl_3$, 300 MHz) spectrum of 6α-hydroxygedunin (**11**).

Figure S68: ^{13}C NMR ($CDCl_3$, 75 MHz) spectrum of 6α-hydroxygedunin (**11**).

Figure S69: DEPT 135º ($CDCl_3$, 75 MHz) spectrum of 6α-hydroxygedunin (**11**).

Figure S70: DEPT 90º ($CDCl_3$, 75 MHz) spectrum of 6α-hydroxygedunin (**11**).

Figure S71: COSY ($CDCl_3$, 300 MHz) spectrum of 6α-hydroxygedunin (**11**).

Figure S72: HSQC ($CDCl_3$, 300; 75 MHz) spectrum of 6α-hydroxygedunin (**11**).

Figure S73: HMBC ($CDCl_3$, 300; 75 MHz) spectrum of 6α-hydroxygedunin (**11**).

7-Deacetyl-6α-butanoyloxygedunin (13):

Figure S74: UFLC-ESI-(+)-HRMS data for 7-deacetyl-6α-butanoyloxygedunin (**13**).

Figure S75: ^{1}H NMR ($CDCl_3$, 300 MHz) spectrum of 6α-butanoyloxygedunin **13**.

Figure S76: ^{13}C NMR ($CDCl_3$, 75 MHz) spectrum of 6α-butanoyloxygedunin **13**.

Figure S77: DEPT 90º ($CDCl_3$, 75 MHz) spectrum of 6α-butanoyloxygedunin **13**.

Figure S78: COSY ($CDCl_3$, 300 MHz) spectrum of 6α-butanoyloxygedunin **13**.

Figure S79: HSQC ($CDCl_3$, 300; 75 MHz) spectrum of 6α-butanoyloxygedunin **13**.

Figure S80: HMBC ($CDCl_3$, 300; 75 MHz) spectrum of 6α-butanoyloxygedunin **13**.

7-Deacetyl-6α-benzoxygedunin (14):

Figure S81: UFLC-PDA-ESI-(+)-HRMS data for new 6α-benzoxygedunin **14**.

Figure S82: ^{1}H NMR ($CDCl_3$, 300 MHz) spectrum of new 6α-benzoxygedunin **14**.

Figure S83: ^{13}C NMR ($CDCl_3$, 75 MHz) spectrum of new 6α-benzoxygedunin **14**.

Figure S84: COSY ($CDCl_3$, 300 MHz) spectrum of new 6α-benzoxygedunin **14**.

Figure S85: HSQC ($CDCl_3$, 300; 75 MHz) spectrum of new 6α-benzoxygedunin **14**.

Figure S86: HMBC ($CDCl_3$, 300; 75 MHz) spectrum of new 6α-benzoxygedunin **14**.

7-Deacetyl-6α-heptanoyloxygedunin (15):

Figure S87: UFLC-PDA-ESI-(+)-HRMS data for new 6α-heptanoyloxygedunin **15**.

Figure S88: ^{1}H NMR ($CDCl_3$, 300 MHz) spectrum of new 6α-heptanoyloxygedunin **15**.

Figure S89: ^{13}C NMR ($CDCl_3$, 75 MHz) spectrum of new 6α-heptanoyloxygedunin **15**.

Figure S90: DEPT 135º ($CDCl_3$, 75 MHz) spectrum of new 6α-heptanoyloxygedunin **15**.

Figure S91: DEPT 90º ($CDCl_3$, 75 MHz) spectrum of new 6α-heptanoyloxygedunin **15**.

Figure S92: COSY ($CDCl_3$, 300 MHz) spectrum of new 6α-heptanoyloxygedunin **15**.

Figure S93: HSQC ($CDCl_3$, 300; 75 MHz) spectrum of new compound **15**.

Figure S94: HMBC ($CDCl_3$, 300; 75 MHz) spectrum of new compound **15**.

3-Deoxo-1,2-dihydro-7-deacetyl-3α-hydroxy-7-*epi*-gedunin (16):

Figure S95: UFLC-PDA-ESI-(+)-HRMS data for new compound **16**.

Figure S96: ^{1}H NMR (C_5D_5N, 300 MHz) spectrum of new compound **16**.

Figure S97: ^{13}C NMR (C_5D_5N, 75 MHz) spectrum of new compound **16**.

Figure S98: DEPT 135º (C_5D_5N, 75 MHz) spectrum of new compound **16**.

Figure S99: COSY (C_5D_5N, 300 MHz) spectrum of new compound **16**.

Figure S100: HSQC (C_5D_5N, 300; 75 MHz) spectrum of new compound **16**.

Figure S101: HMBC (C_5D_5N, 300; 75 MHz) spectrum of new compound **16**.

Summary

Presented herein are: a) descriptions of the three different methods used in the isolation of gedunin derivatives **1**, **11** and **12**, b) UFLC-PDA chromatograms, UFLC-HRMS chromatograms and spectra, and 1D and 2D NMR spectra for gedunin (**4**) and gedunin derivatives **2–3**, **5-9**, **11** and **13–16** and 3) our assigned ^{1}H and ^{13}C NMR chemical shift data for 7-deacetylgedunin (**2**), gedunin (**4**) and 7-deacetyl-7α-butanoyloxygedunin (**6**) (Figure S1).

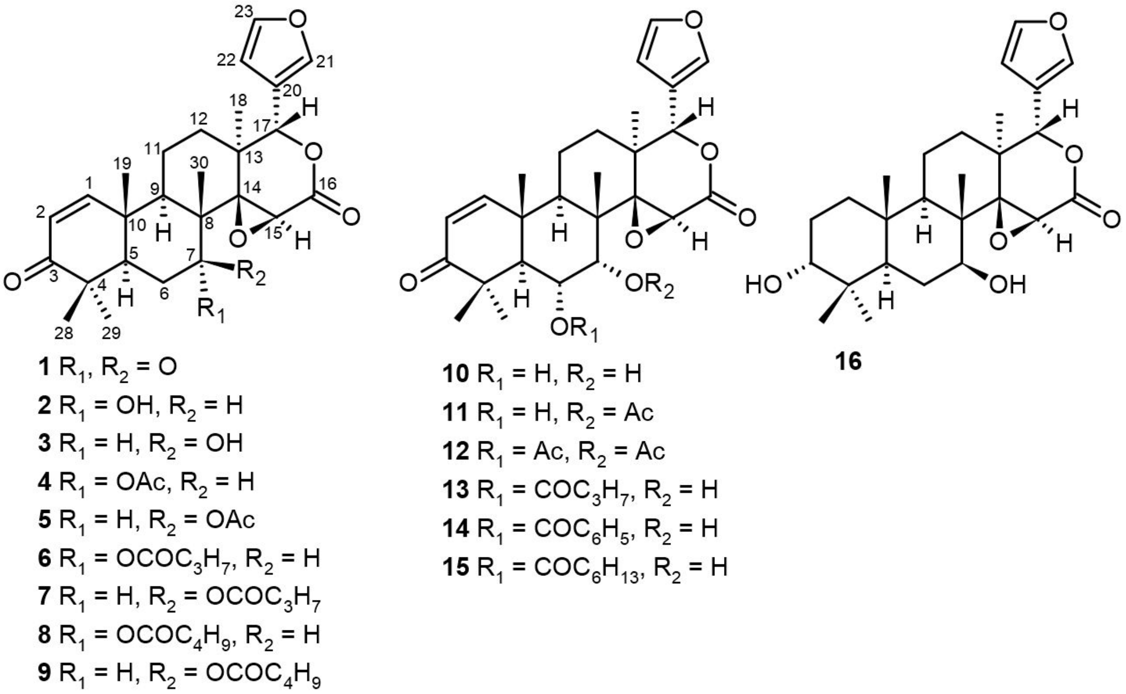

Figure S1. Structures of gedunin derivatives isolated from *Carapa* spp. (**1**, **11**, **12**) and semisynthetic compounds gedunin (**4**) and derivatives (**2**, **3**, **5–10**, **13–16**).

Limonoid isolation method 1

Multigram scale isolations of cedrolide (1) and 6α-acetoxygedunin (12). At the Roque Community Oil Plant in Carauari, Amazonas State (S 4° 54’ W 66° 55’), the procedure for carapa (andiroba) oil production was as follows. First, *Carapa guianensis* (and presumably also *C. procera* DC.) seeds were ground to a pulp. Next, the ground seed

materials were heated with constant stirring on an open air, steel drying plate for 1 h. Then, the resulting dried seed mass was pressed at 90ºC to obtain carapa oil and residual seed cake (RSC). The RSC (10 kg) was extracted (2 × 6 h) with hexanes, then with EtOH, in a semi-industrial Soxhlet apparatus. The EtOH extracts were allowed to stand at room temperature (r.t.) for 10 days. After this time, an amorphous, limonoid-rich solid had precipitated and was collected by r.t. filtration to yield the limonoid rich fraction (LRF, 4.30 g, 0.043% based on RSC). Repetition of the extraction, precipitation and partition procedures a total of four more times provided a total of 38.9 g (0.078%) of LRFs from an estimated 50 kg of RSC. Recrystallization of an aliquot of the LRF (7.4 g) from Carauari from EtOH furnished crystals that underwent further purification by preparative reverse-phase HPLC using isocratic ACN/H_2O (7:3) as mobile phase, a flow rate of 15 mL/min and monitoring at a wavelength of 220 nm. Repetition of the HPLC procedure provided **1** (3.9 g, 53%) and **12** (2.6 g, 35%).

Limonoid isolation method 2

*Multi-milligram scale isolations of cedrolide (**1**), 6α-hydroxygedunin (**11**) and 6α-acetoxygedunin (**12**).* In Manaus Municipality, *C. guianensis* seeds were collected at INPA´s Adolpho Ducke Forest Reserve from an individual tree previously identified in the INPA Herbarium (voucher INPA 203918). Preparation of the LRF at INPA´s Semi-Industrial Extraction Laboratory was performed as follows. First, seeds were selected that showed no signs of damage (as by insects or fungi). Next, seeds were washed with H_2O, dried in a forced air flow oven at 40ºC for 5 days and then ground. Extraction of the ground seed materials (10 kg) with hexanes (1 × 6 h) in a semi-industrial Soxhlet apparatus provided the LRF (3.48 g, 0.035%) after r.t. filtration of this precipitate from the hexane extracts. Chromatographic separation of the LRF (3.4 g, 0.034%) from

Manaus Municipality on a column of silica gel 60 (63–20 mm mesh, ∅ × h = 4 × 27 cm) in an increasing polarity gradient of EtOAc (10 to 40%) in hexanes provided a semi-pure fraction. Further purification of this fraction (50 mg/mL) by repeated semi-preparative HPLC with isocratic ACN/H_2O (3:2), a flow rate of 15 mL/min, and monitoring at a wavelength of 220 nm, provided limonoids **1** (61.6 mg, 1.8%), **11** (11.5 mg, 0.34%) and **12** (146.9 mg, 4.3%).

Limonoid isolation method 3

*Multigram scale isolation of 6α-acetoxygedunin (**12**). Carapa* spp. seeds (22 kg) were collected in Parintins in the Djard Vieira district (S 2º 38' 15" W 56º 43' 44"), then boiled in H_2O. After boiling, the softened seeds were kneaded into a paste or "bread". The seed paste was loaded into a "tipiti" (chee-pee-CHEE) strainer/press from which carapa oil (1.5 L) filtered out and was collected. The resulting seed materials (19 kg) were removed from the tipiti and placed in cotton bags. Then, the cotton bags were centrifuged in a washing machine (Brastemp, Clean model, Brazil). Centrifuging resulted in more carapa oil (3 L) and RSC (15 kg). This process was carried out by students of the Chemistry Teacher Education Program at the Center for Advanced Studies in Parintins (CESP) of the State University of Amazonas (UEA) under the guidance of Prof. Célia Maria Serrão Eleutério and one of the authors (D.S.P.). The RSC (15 kg) was kindly donated to this study. At LAPAAM/INPA, the RSC (15 kg) was next ground in a common blender, sieved (0.25 mm pore size), and the finely divided RSC was stored at r.t. Then, a portion of the powdered RSC (400 g) underwent continuous acetone extraction in a Soxhlet apparatus (3 × 6 h). Next, the resulting extracts were filtered, combined, and concentrated under reduced pressure on a rotary evaporator to yield crude acetone extract (138 g). Subsequently, the crude acetone extract (128 g) was refluxed in MeOH (300 mL) for 1 h.

After reflux, the resulting mixture was filtered, and the filtrate was allowed to stand at r.t. After 7 days, needle-like crystals formed and were collected by vacuum filtration, washed with cold MeOH, and dried in a sand bath at 40-50ºC. The yield of crystals (LRF) was 9.5 g. Next, LRF (6.5 g) was chromatographed on a column of silica gel 60 (0.063–0.200 mm, Merck) and eluted with a gradient of hexanes and EtOAc (9:1 to 3:2), EtOAc (100%) and finally MeOH (100%). Fractions were combined based on TLC analysis to yield 6α-acetoxygedunin (**12**, 3.8 g).

7-Deacetylgedunin (2):

^{1}H NMR ($CDCl_3$, 300 MHz): δ 7.41 (2H, *m*, H21 and H23), 7.11 (1H, *d*, *J* = 10.2 Hz, H1), 6.35 (1H, *m*, H22), 5.86 (1H, *d*, *J* = 10.2 Hz, H2), 5.60 (1H, *s*, H17), 3.91 (1H, *s*, H15), 3.58 (1H, *m*, H7), 2.5–2.6 (1H, *m*, H9), 2.4–2.5 (1H, *m*, H5), 1.94* (*m*, H11α), 1.90* (*m*, H6α), 1.80* (*m*, H11β), 1.66* (*m*, H12β), 1.66* (*m*, H6β), 1.60* (*m*, H12α), 1.24 (3H, *s*, H18), 1.20 (3H, *s*, H29), 1.15 (3H, *s*, H28), 1.10 (3H, *s*, H19), 1.09 (3H, *s*, H30). Note: *chemical shift ascertained from the HSQC spectrum. ^{13}C NMR ($CDCl_3$, 75 MHz): δ 204.6 (*s*, C3), 168.3 (*s*, C16), 157.8 (*d*, C1), 143.0 (*d*, C21), 141.2 (*d*, C23), 125.8 (*d*, C2), 120.6 (*s*, C20), 110.0 (*d*, C22), 78.5 (*d*, C17), 70.0 (*s*, C14), 69.7 (*d*, C7), 57.8 (*d*, C15), 44.6 (*d*, C5), 44.2 (*s*, C4), 43.6 (*s*, C8), 40.2 (*s*, C10), 38.3 (*s*, C13), 37.9 (*d*, C9), 27.3 (*t*, C12), 27.3 (*q*, C28), 26.4 (*t*, C6), 21.5 (*q*, C19), 19.9 (*q*, C29), 18.7 (*q*, C30), 17.8 (*q*, C18), 15.0 (*t*, C11).

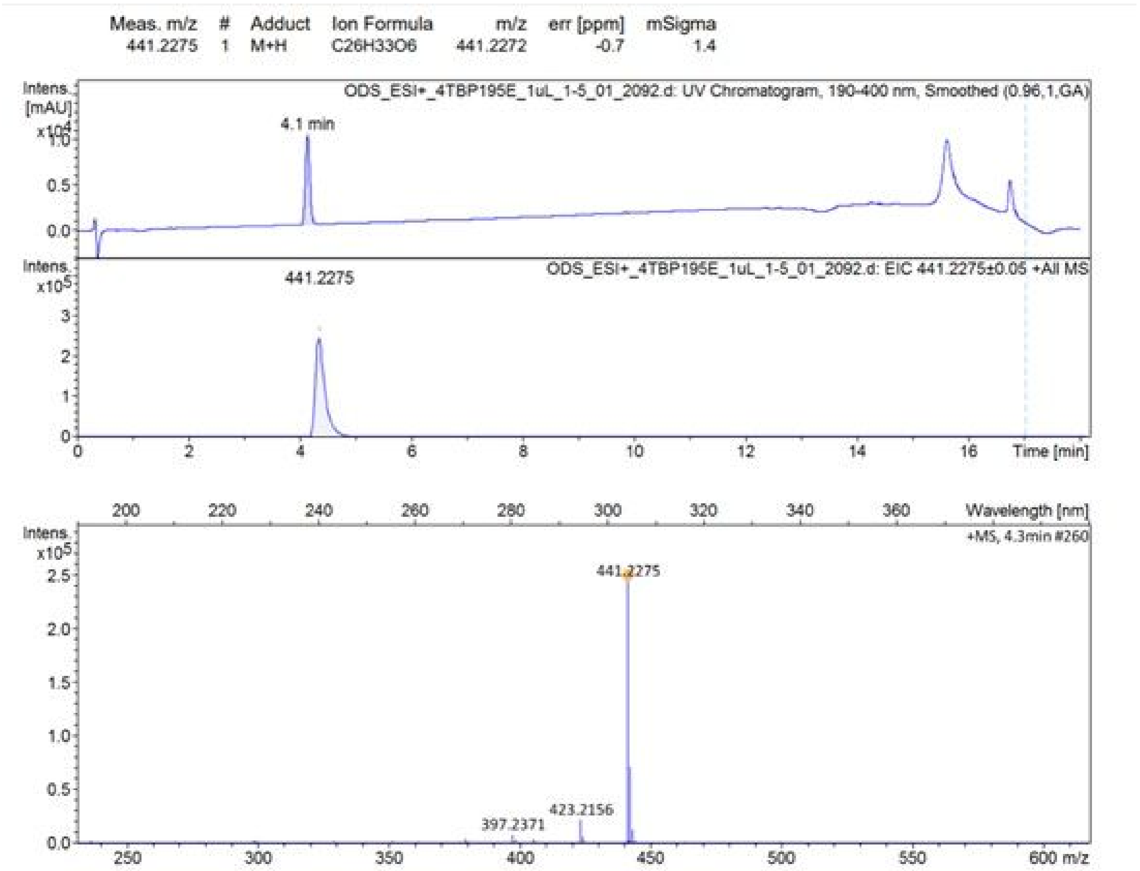

Figure S2: UFLC-PDA-ESI (+)-HRMS data for 7-deacetylgedunin (**2**).

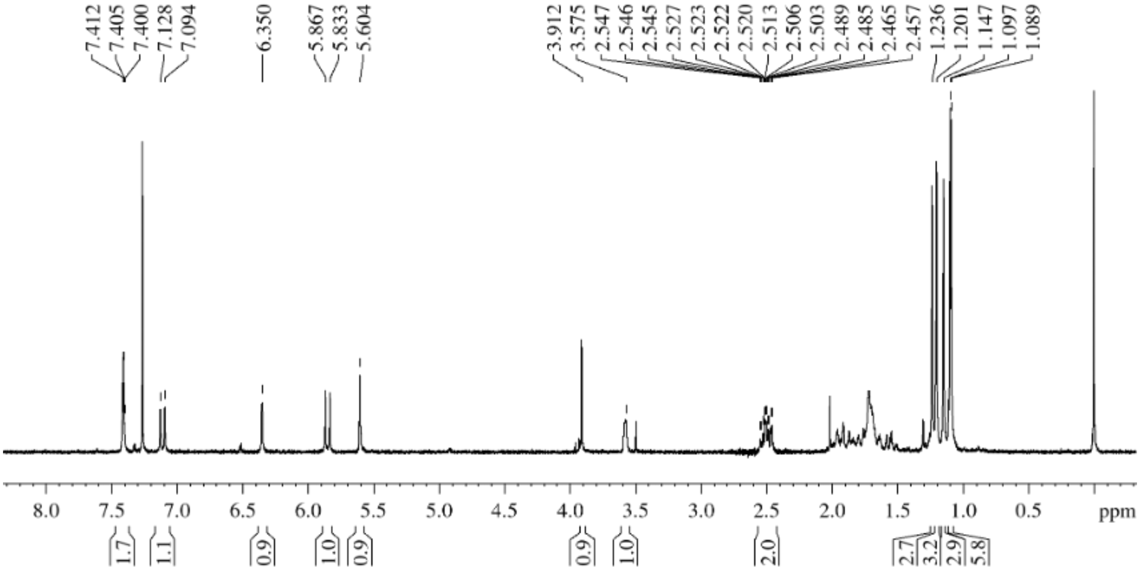

Figure S3: ^{1}H NMR ($CDCl_3$, 300 MHz) spectrum of 7-deacetylgedunin (**2**).

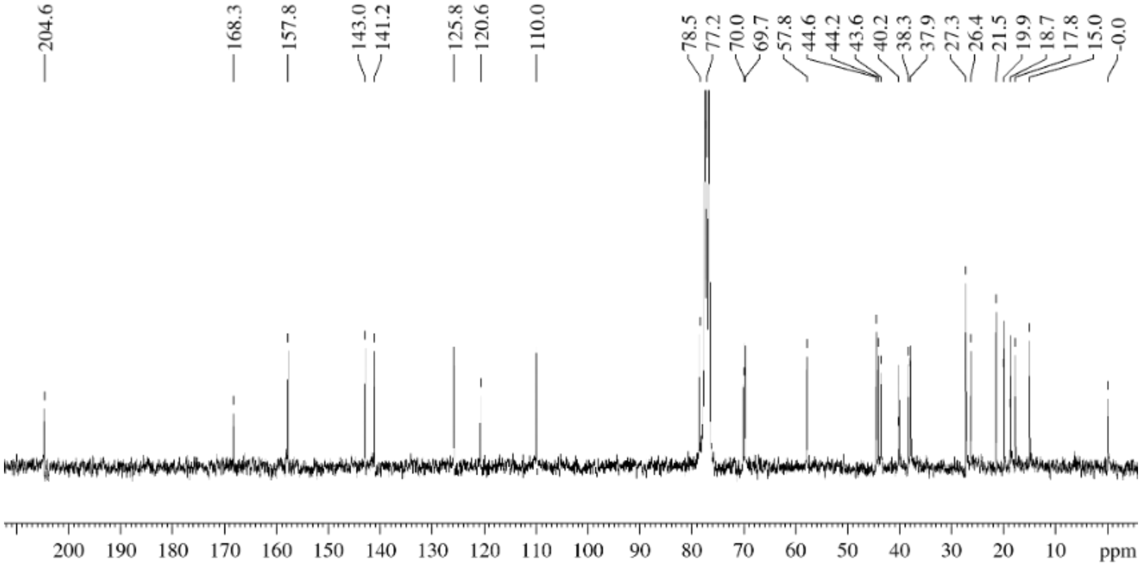

Figure S4: ^{13}C NMR ($CDCl_3$, 75 MHz) spectrum of 7-deacetylgedunin (**2**).

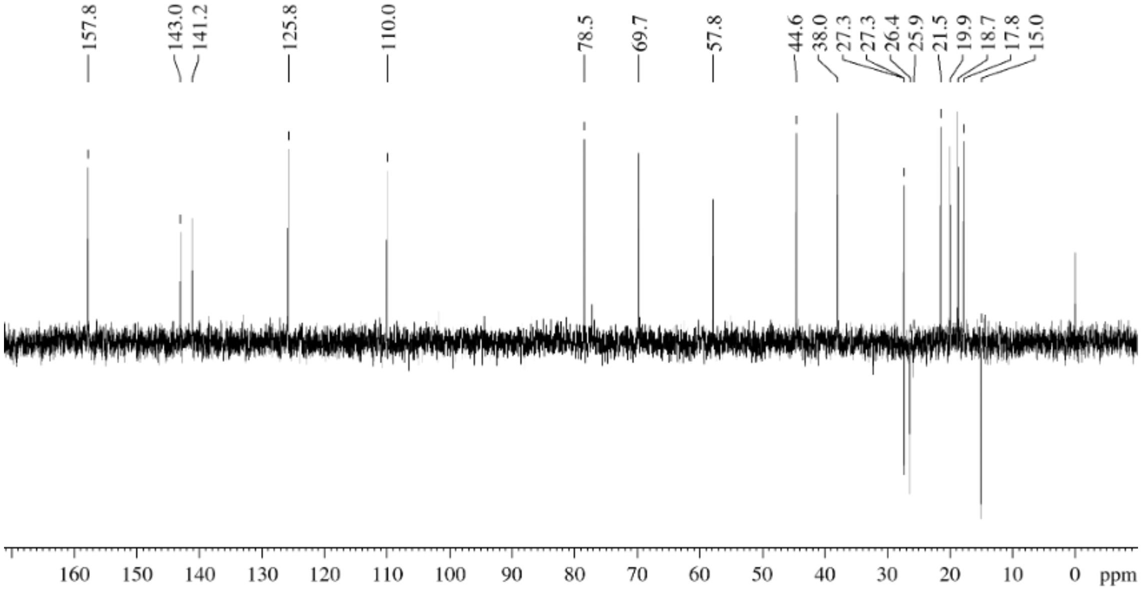

Figure S5: DEPT 135º ($CDCl_3$, 75 MHz) spectrum of 7-deacetylgedunin (**2**).

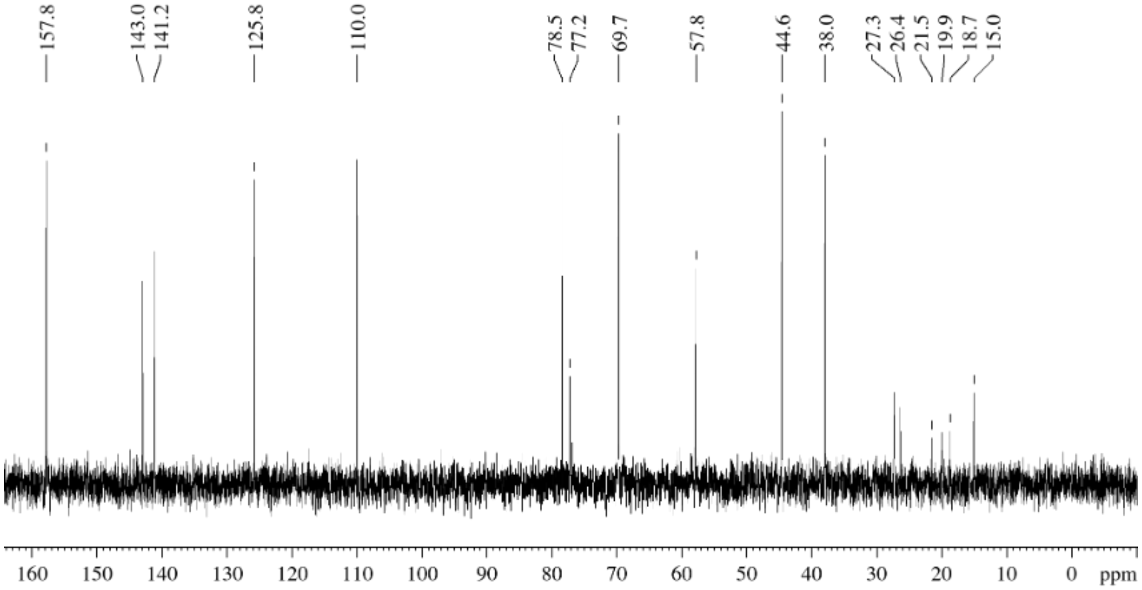

Figure S6: DEPT 90º ($CDCl_3$, 75 MHz) spectrum of 7-deacetylgedunin (**2**).

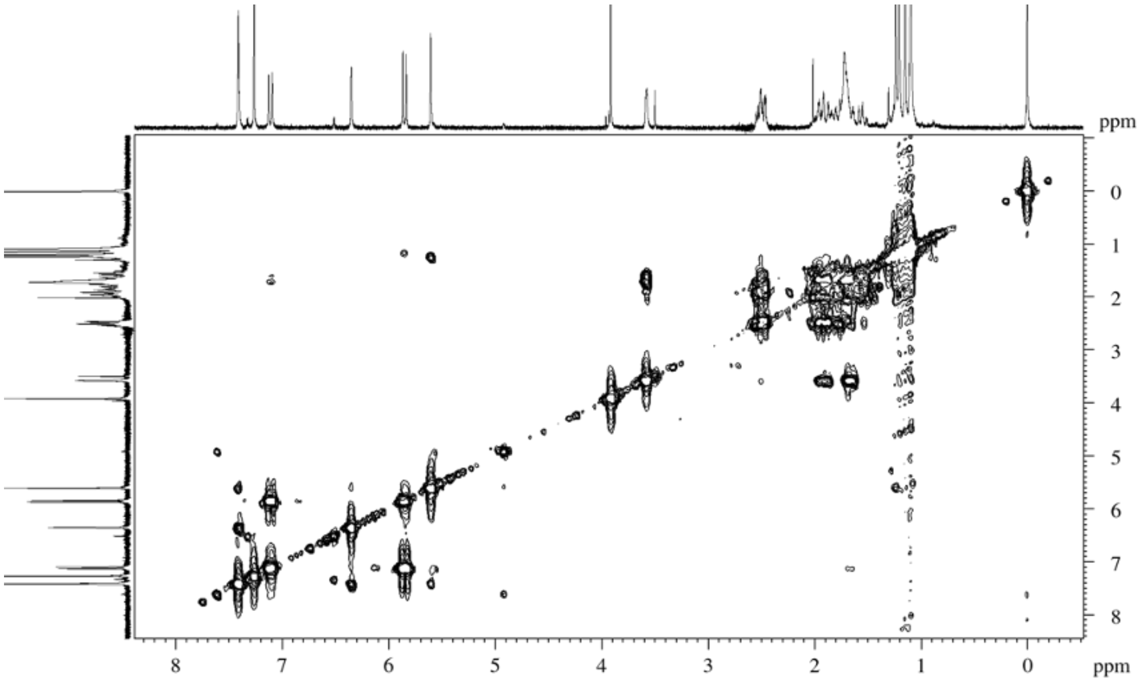

Figure S7: COSY ($CDCl_3$, 300 MHz) spectrum of 7-deacetylgedunin (**2**).

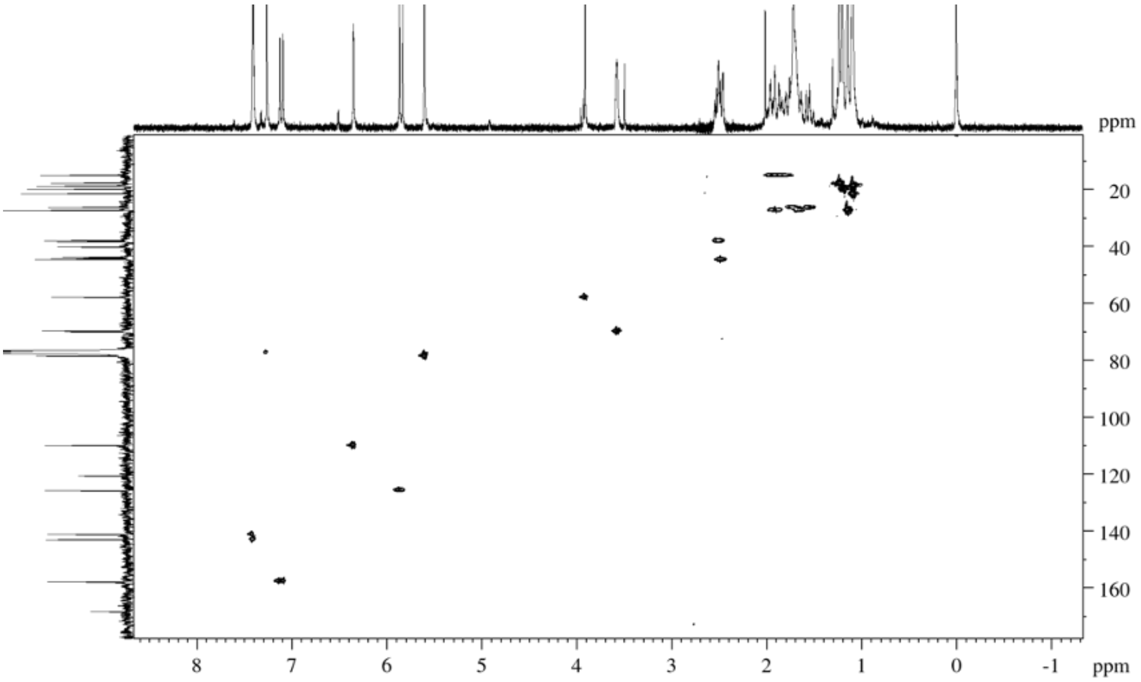

Figure S8: HSQC ($CDCl_3$, 300; 75 MHz) spectrum of 7-deacetylgedunin (**2**).

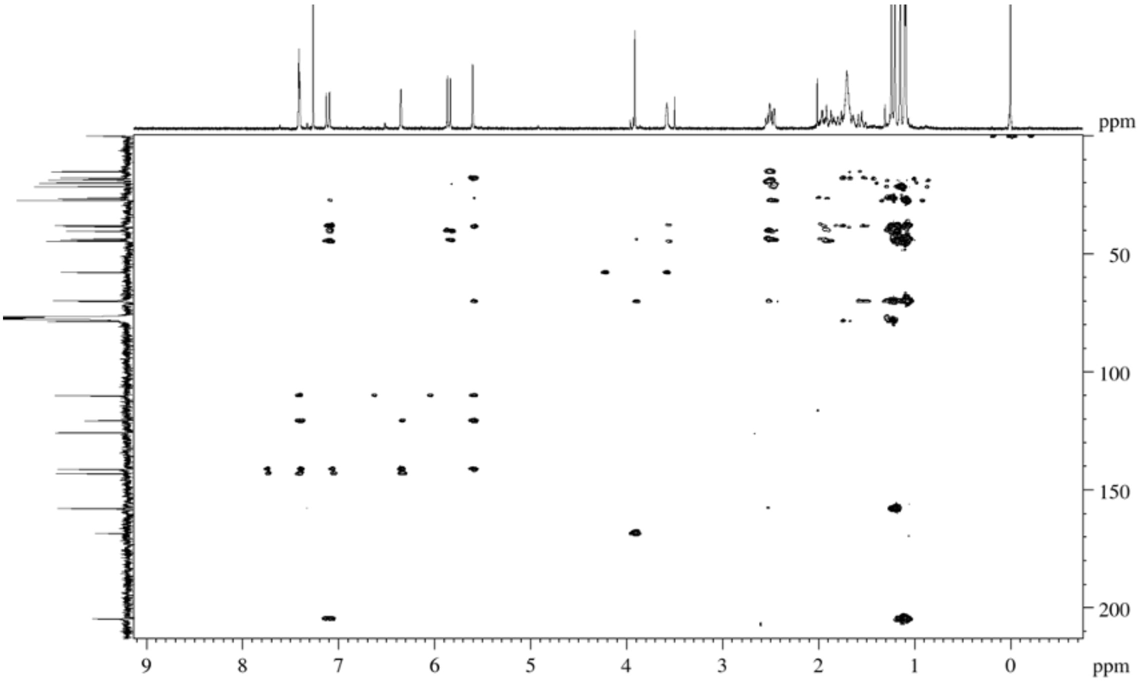

Figure S9: HMBC ($CDCl_3$, 300; 75 MHz) spectrum of 7-deacetylgedunin (**2**).

7-Deacetyl-7-*epi*-gedunin (3):

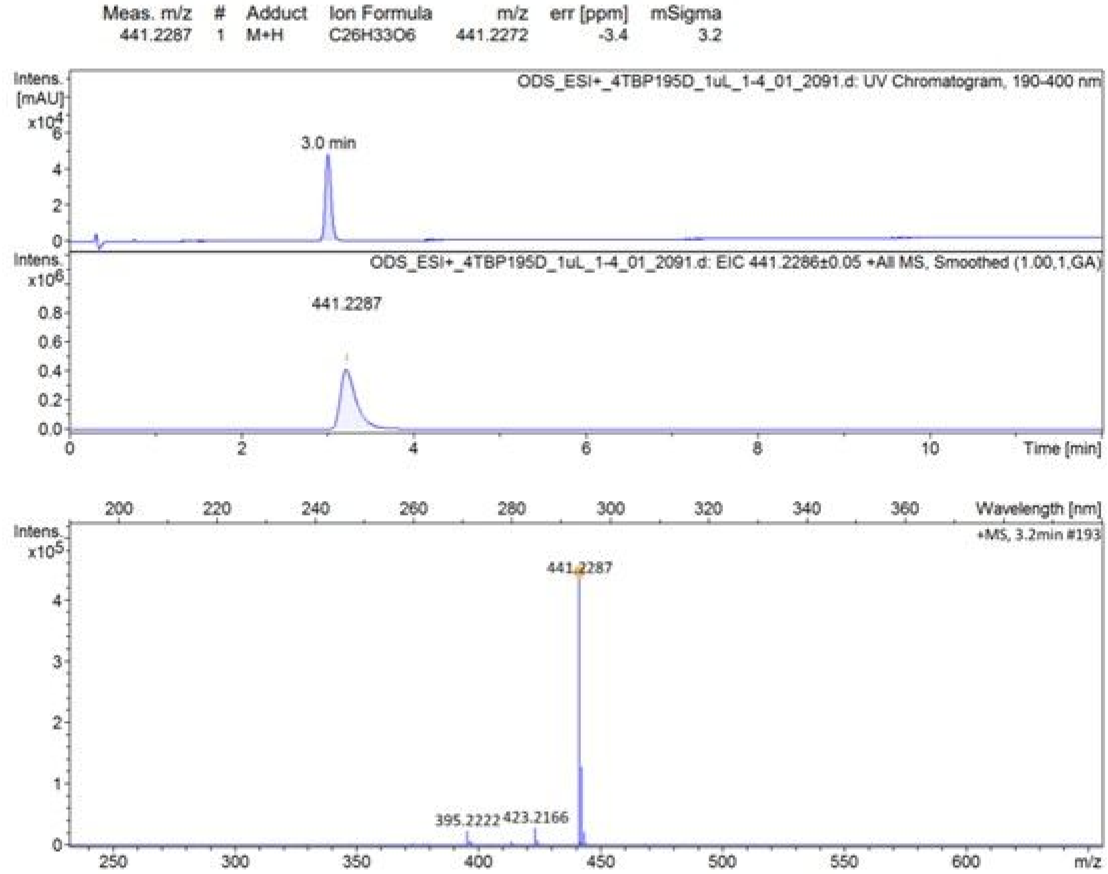

Figure S10: UFLC-PDA-ESI-(+)-HRMS data for 7-deacetyl-7-*epi*-gedunin (**3**).

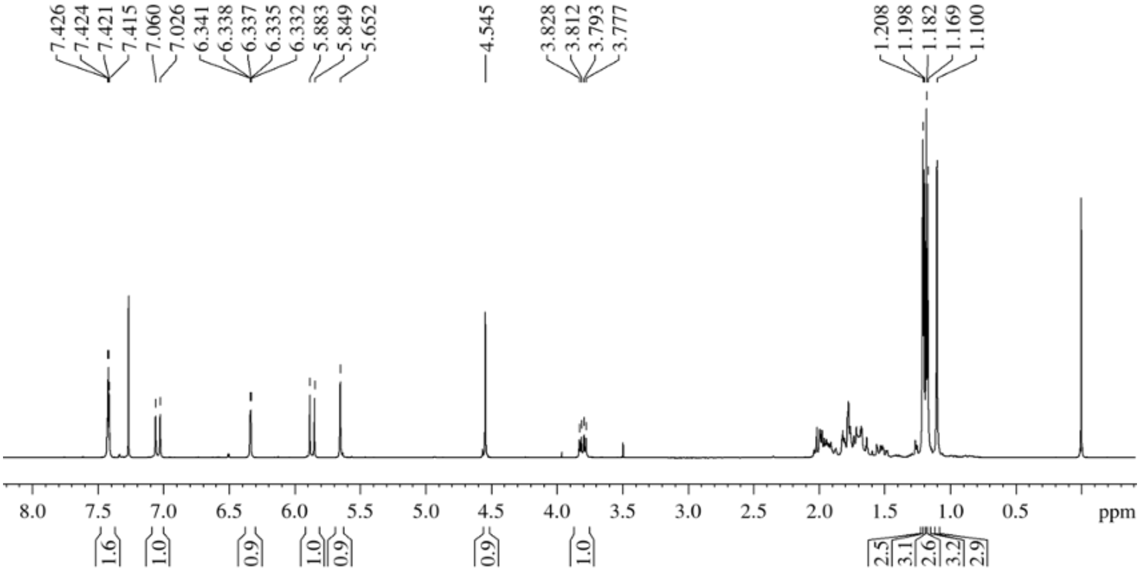

Figure S11: ^{1}H NMR ($CDCl_3$, 300 MHz) spectrum of 7-deacetyl-7-*epi*-gedunin (**3**).

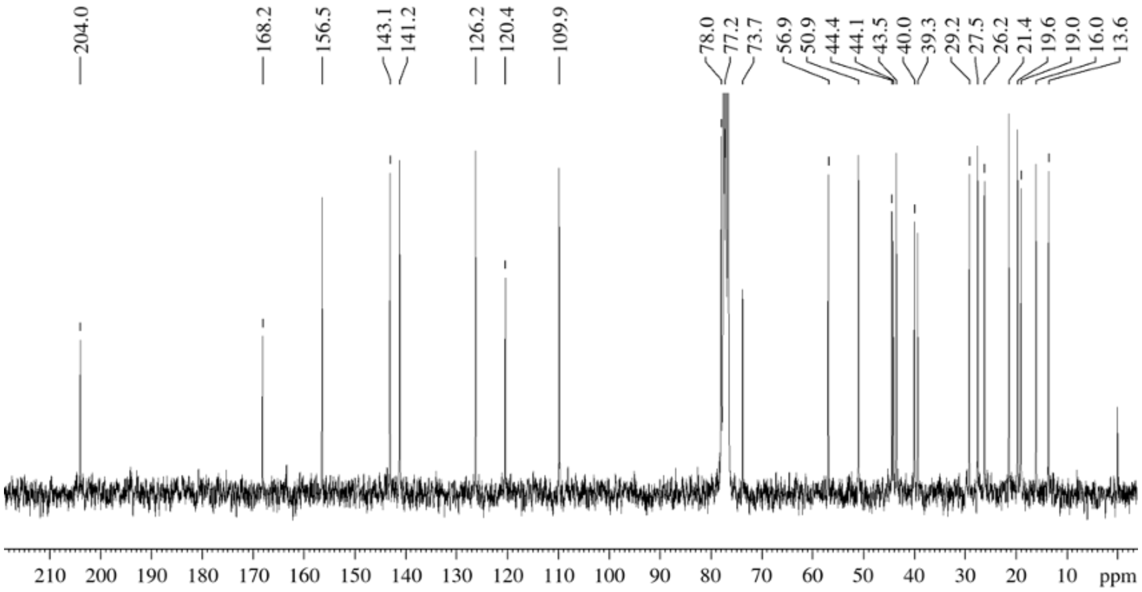

Figure S12: ^{13}C NMR ($CDCl_3$, 75 MHz) spectrum of 7-deacetyl-7-*epi*-gedunin (**3**).

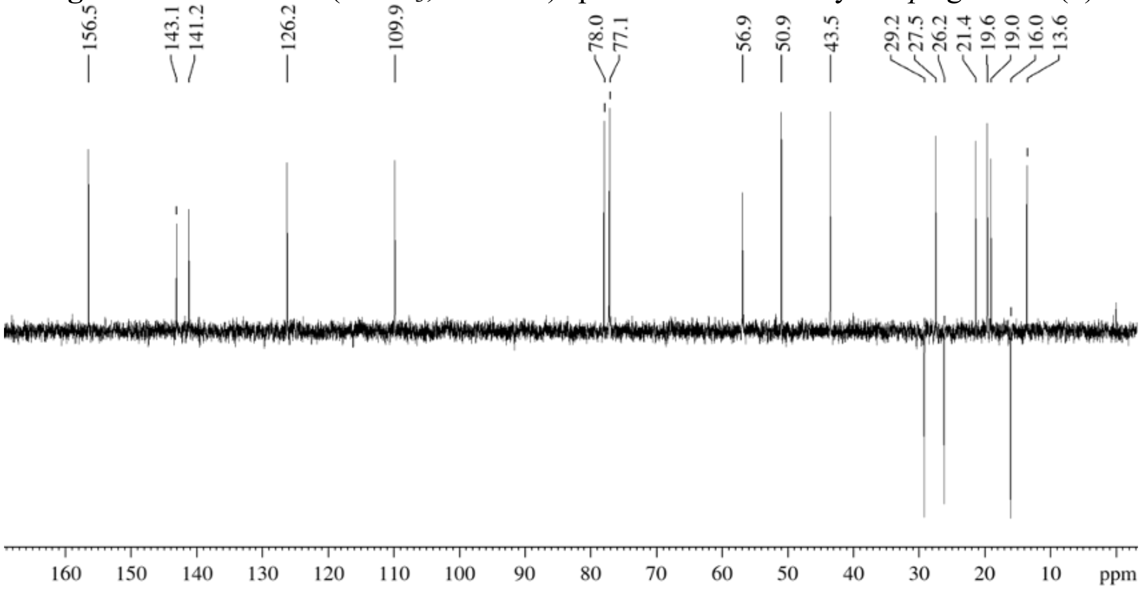

Figure S13: DEPT 135º ($CDCl_3$, 75 MHz) spectrum of 7-deacetyl-7-*epi*-gedunin (**3**).

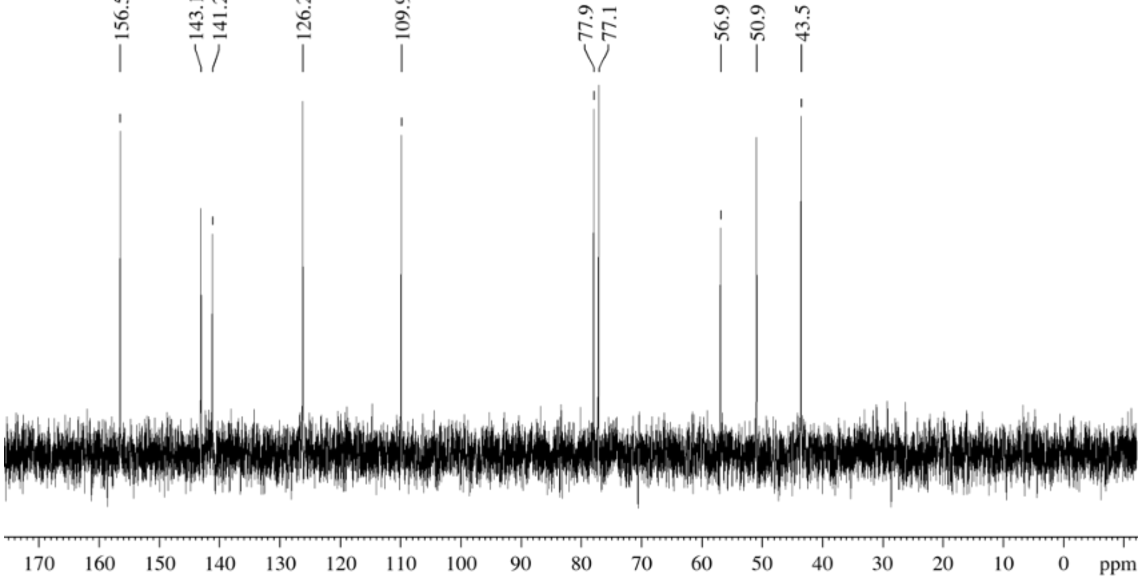

Figure S14: DEPT 90º ($CDCl_3$, 75 MHz) spectrum of 7-deacetyl-7-*epi*-gedunin (**3**).

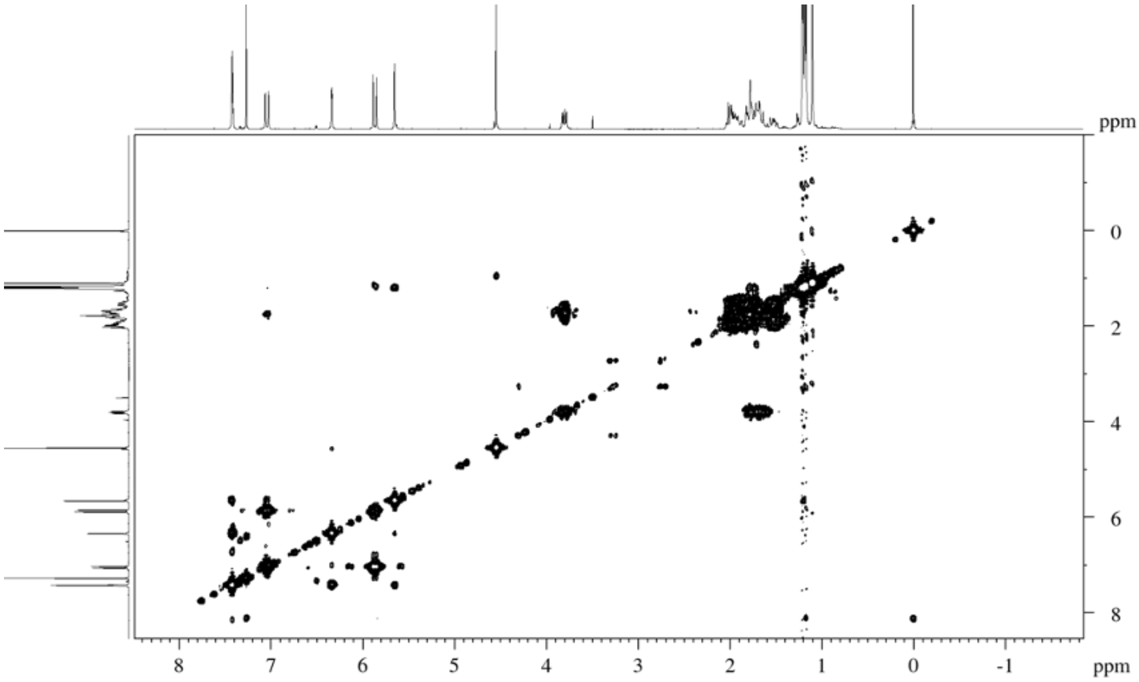

Figure S15: COSY ($CDCl_3$, 300 MHz) spectrum of 7-deacetyl-7-*epi*-gedunin (**3**).

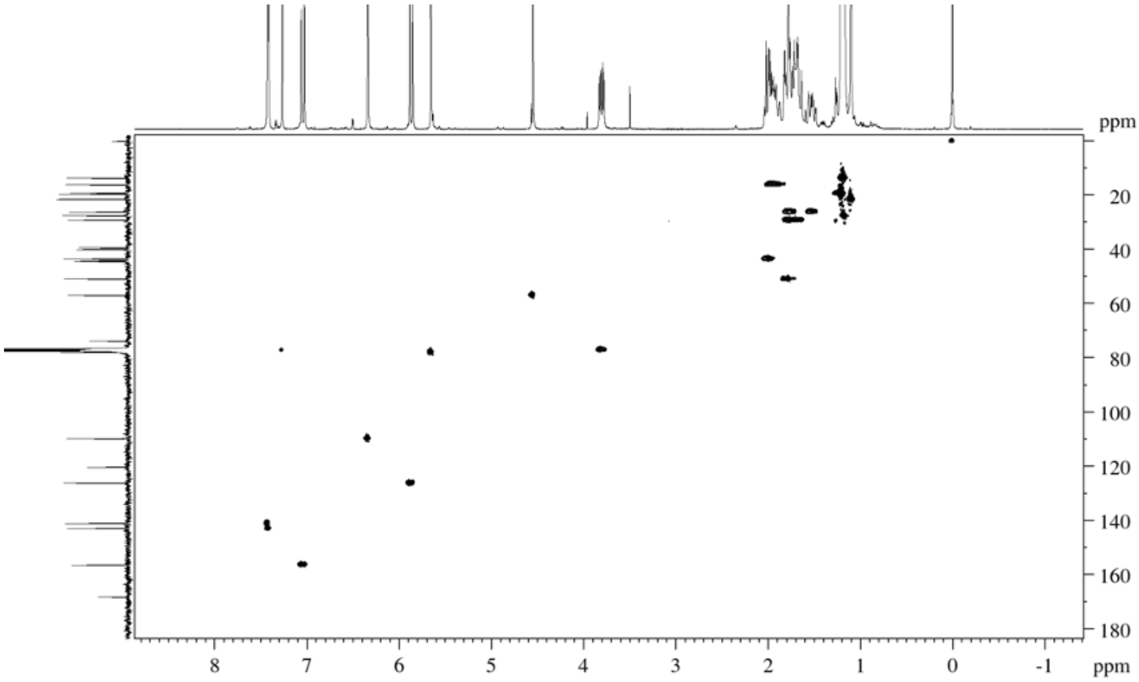

Figure S16: HSQC ($CDCl_3$, 300; 75 MHz) spectrum of 7-deacetyl-7-*epi*-gedunin (**3**).

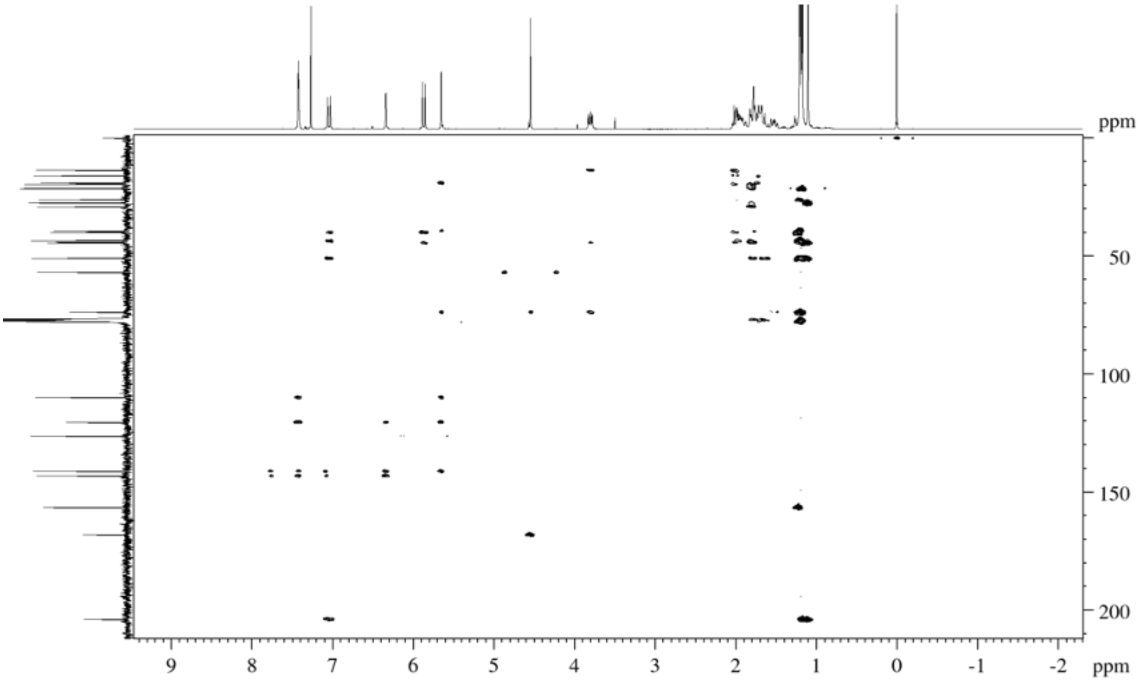

Figure S17: HMBC ($CDCl_3$, 300; 75 MHz) spectrum of deacetyl-7-*epi*-gedunin (**3**).

Gedunin (4):

^{1}H NMR ($CDCl_3$, 300 MHz): δ 7.42 (1H, *m*, H23), 7.42 (1H, *m*, H21), 7.10 (1H, *d*, *J* = 10.2 Hz, H1), 6.34 (1H, *t*, *J* = 1.4 Hz, H22), 5.87 (1H, *d*, *J* = 9.9 Hz, H2), 5.62 (1H, *s*, H17), 4.56 (1H, *dd*, *J* = 3.3, 2.1 Hz, H7), 3.53 (1H, *s*, H15), 2.49 (1H, *dd*, *J* = 12.8, 6.3 Hz, H9), 2.17 (1H, *dd*, *J* = 13.1, 2.6 Hz, H5), 2.11 (3H, *s*, $C\underline{H}_3CO$), 2.00* (*m*, H11α), 1.92* (*m*, H6α), 1.87* (*m*, H11β), 1.81* (*m*, H6β), 1.73* (*m*, H12β), 1.60* (*m*, H12α), 1.25 (3H, *s*, H18), 1.23 (3H, *s*, H19), 1.16 (3H, *s*, H30), 1.08 (3H, *s*, H28), 1.07 (3H, *s*, H29). Note: *chemical shift ascertained from the HSQC spectrum. ^{13}C NMR ($CDCl_3$, 75 MHz): δ 204.0 (*s*, C3), 169.9 (*s*, $CH_3\underline{C}O$), 167.5 (*s*, C16), 157.0 (*d*, C1), 143.1 (*d*, C21), 141.2 (*d*, C23), 126.0 (*d*, C2), 120.4 (*s*, C20), 109.9 (*d*, C22), 78.2 (*d*, C17), 73.2 (*d*, C7), 69.8 (*s*, C14), 56.9 (*d*, C15), 46.0 (*d*, C5), 44.0 (*s*, C4), 42.6 (*s*, C8), 40.0 (*s*, C10), 39.5 (*d*, C9), 38.7 (*s*, C13), 27.2 (*q*, C28), 26.0 (*t*, C12), 23.2 (*t*, C6), 21.2 (*q*, C29), 21.1 (*q*, $\underline{C}H_3CO$), 19.8 (*q*, C19), 18.3 (*q*, C30), 17.7 (*q*, C18), 14.9 (*t*, C11).

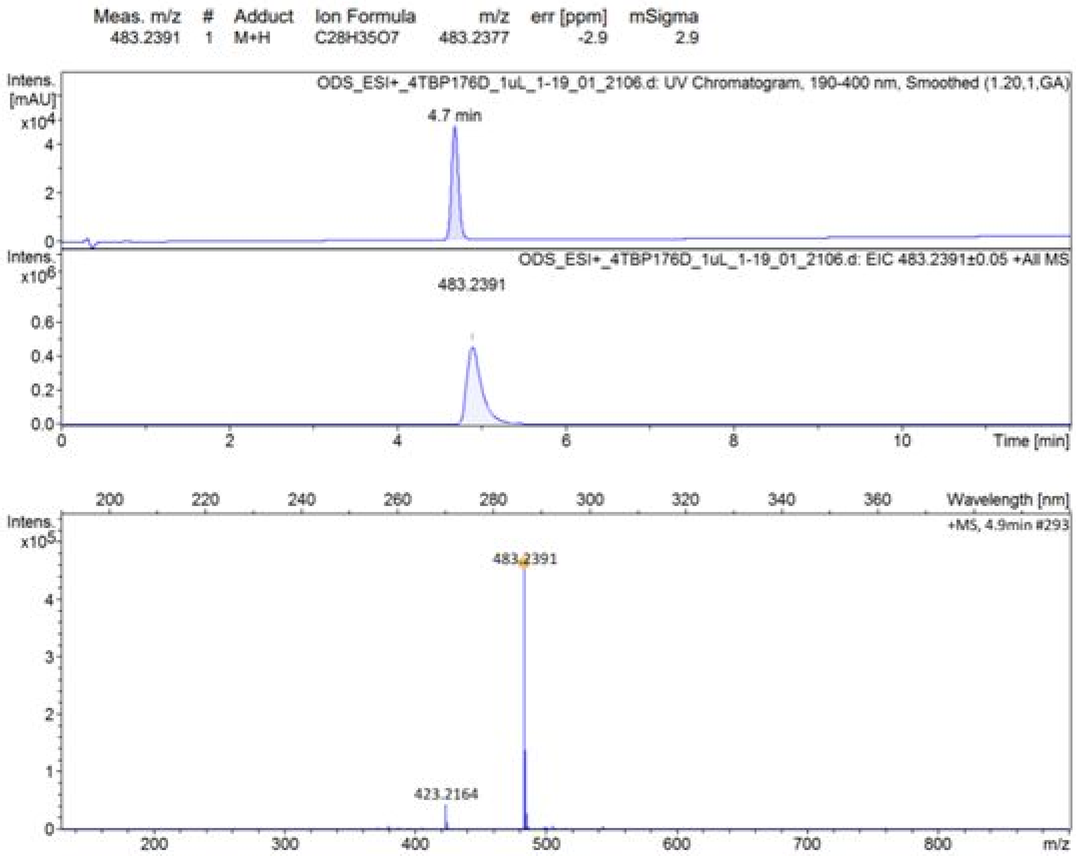

Figure S18: UFLC-PDA-ESI-(+)-HRMS data for gedunin (**4**).

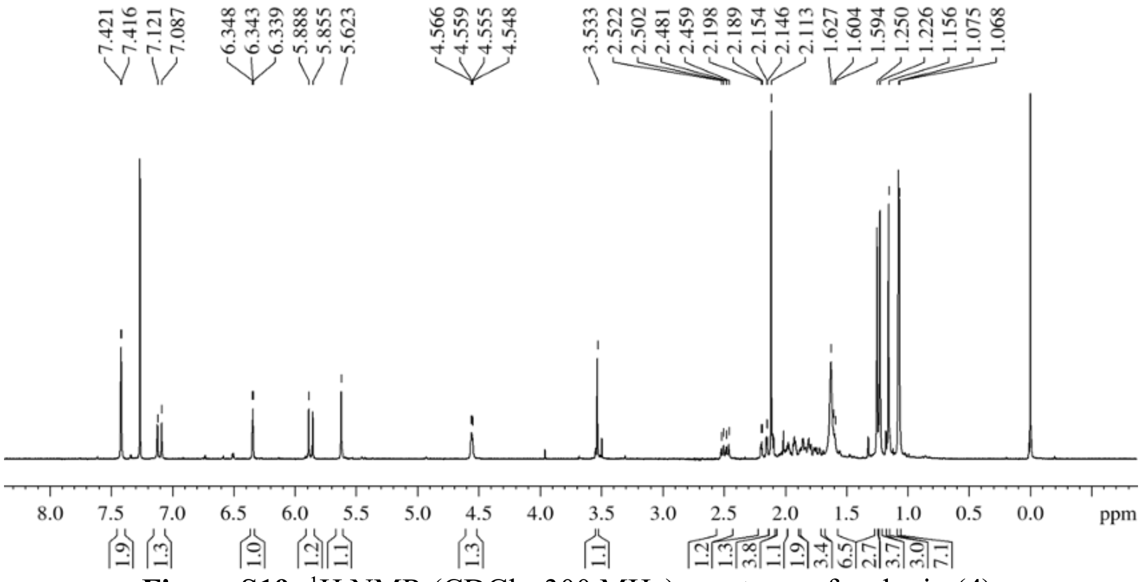

Figure S19: ^{1}H NMR ($CDCl_3$, 300 MHz) spectrum of gedunin (**4**).

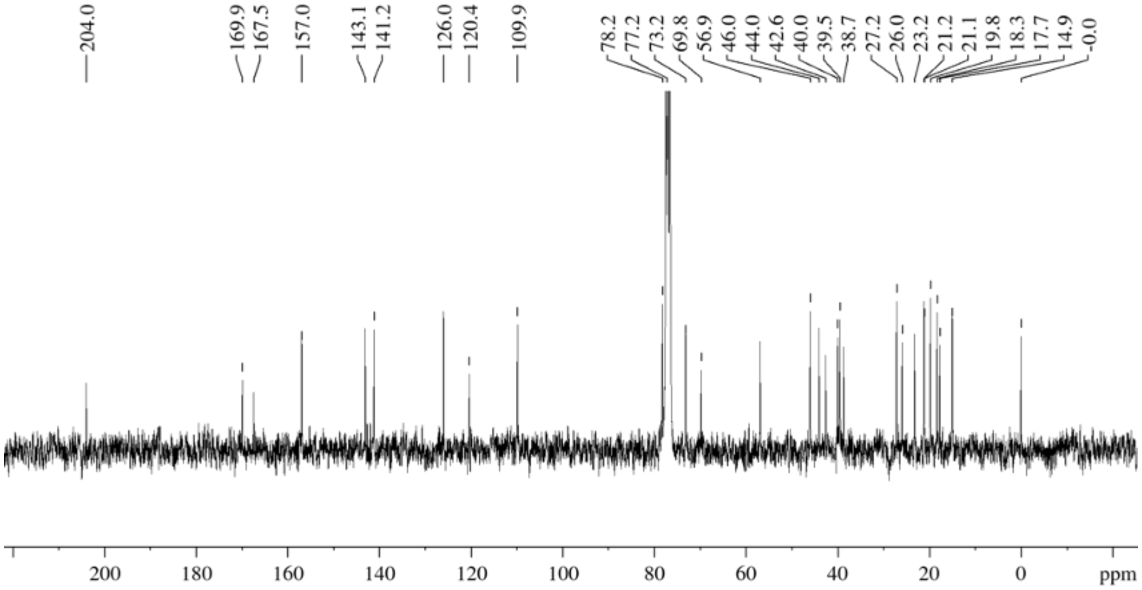

Figure S20: ^{13}C NMR ($CDCl_3$, 75 MHz) spectrum of gedunin (**4**).

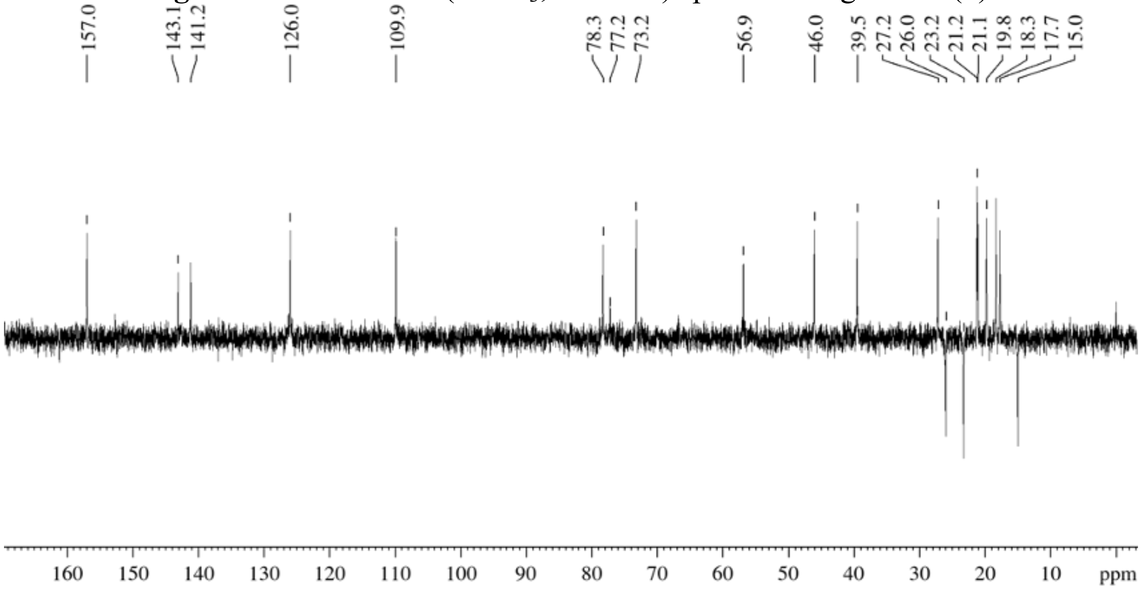

Figure S21: DEPT 135º ($CDCl_3$, 75 MHz) spectrum gedunin (**4**).

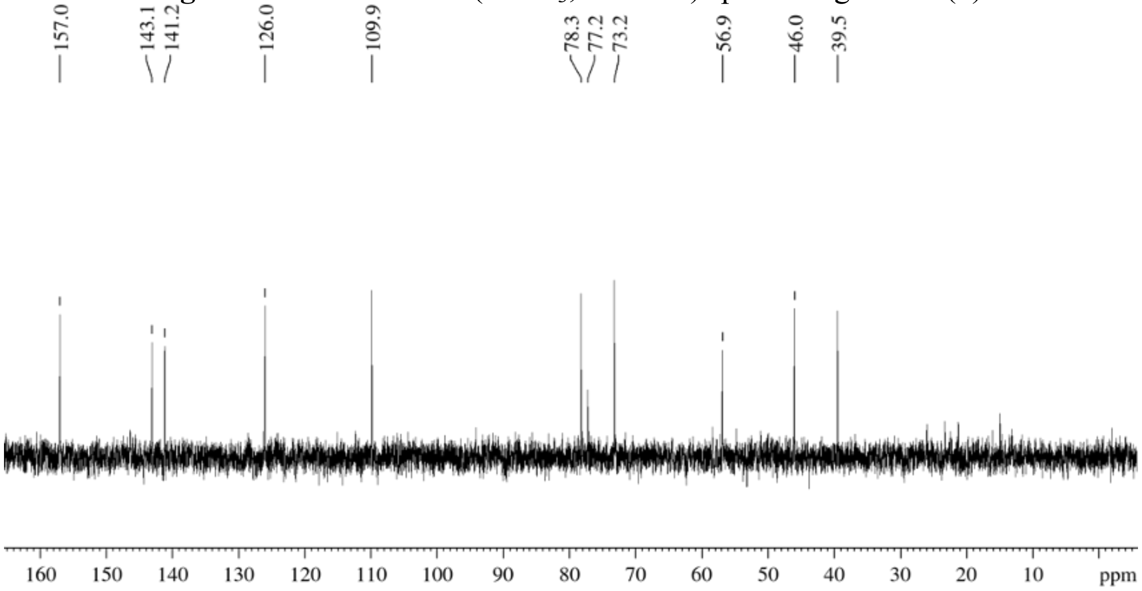

Figure S22: DEPT 90º ($CDCl_3$, 75 MHz) spectrum of gedunin (**4**).

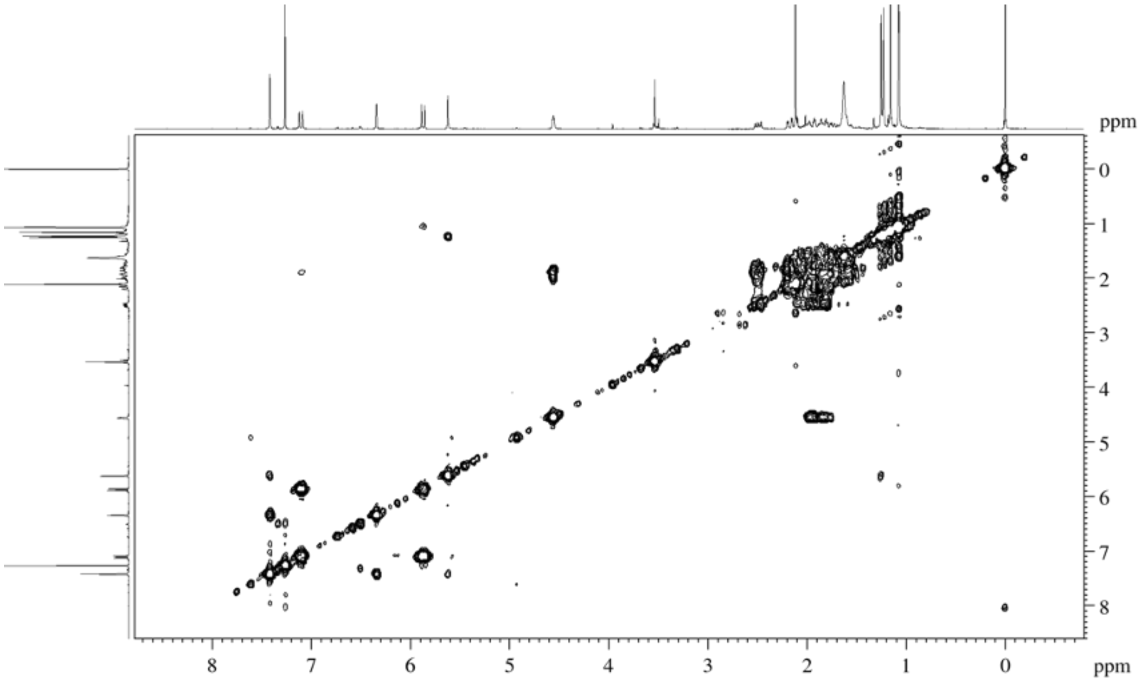

Figure S23: COSY ($CDCl_3$, 300 MHz) spectrum of gedunin (**4**).

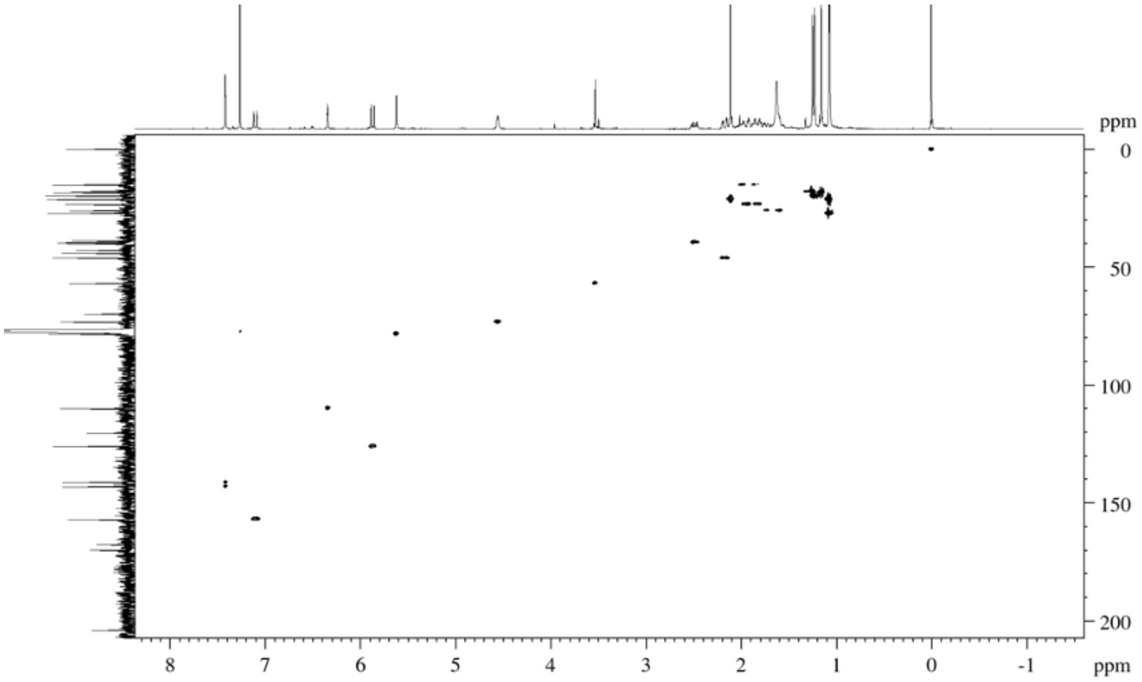

Figure S24: HSQC ($CDCl_3$, 300; 75 MHz) spectrum of gedunin (**4**).

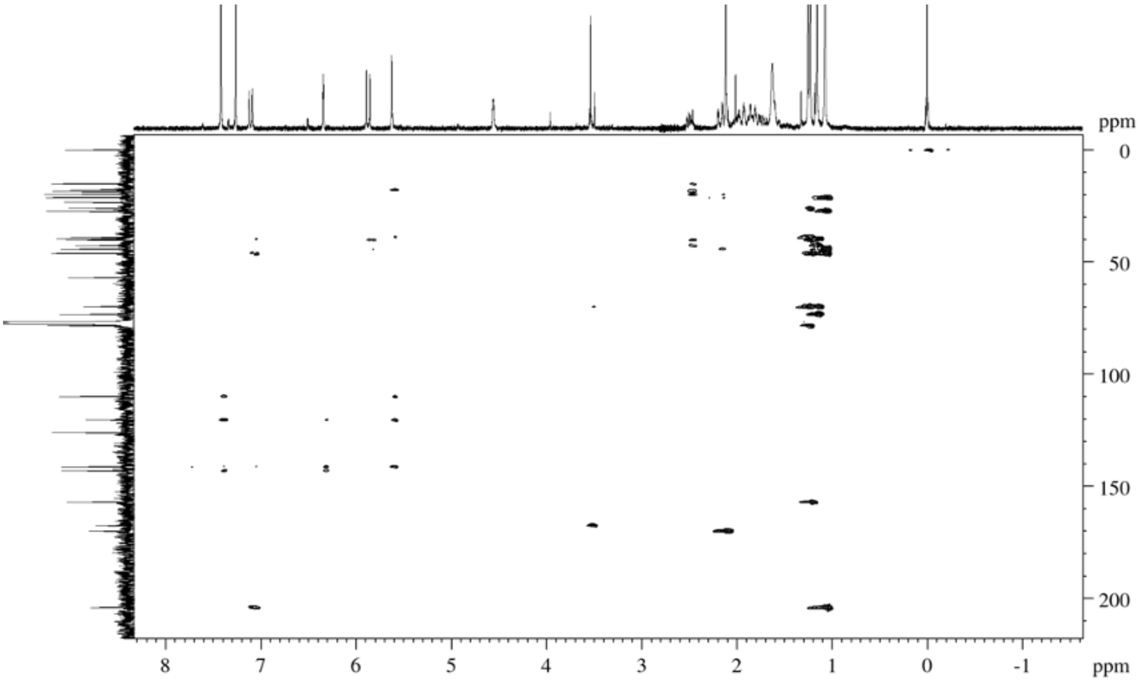

Figure S25: HMBC ($CDCl_3$, 300; 75 MHz) spectrum of gedunin (**4**).

7-*Epi*-gedunin (5):

Meas. m/z	#	Ion Formula	m/z	err [ppm]	mSigma	# mSigma	Score	rdb	e⁻ Conf	N-Rule
483.2406	1	C28H35O7	483.2377	-6.0	3.1	1	41.78	11.5	even	ok
	2	C29H31N4O3	483.2391	-3.3	14.8	2	100.00	16.5	even	ok
	3	C21H39O12	483.2436	6.1	33.9	3	21.00	2.5	even	ok
	4	C34H31N2O	483.2431	5.1	38.3	4	29.82	20.5	even	ok

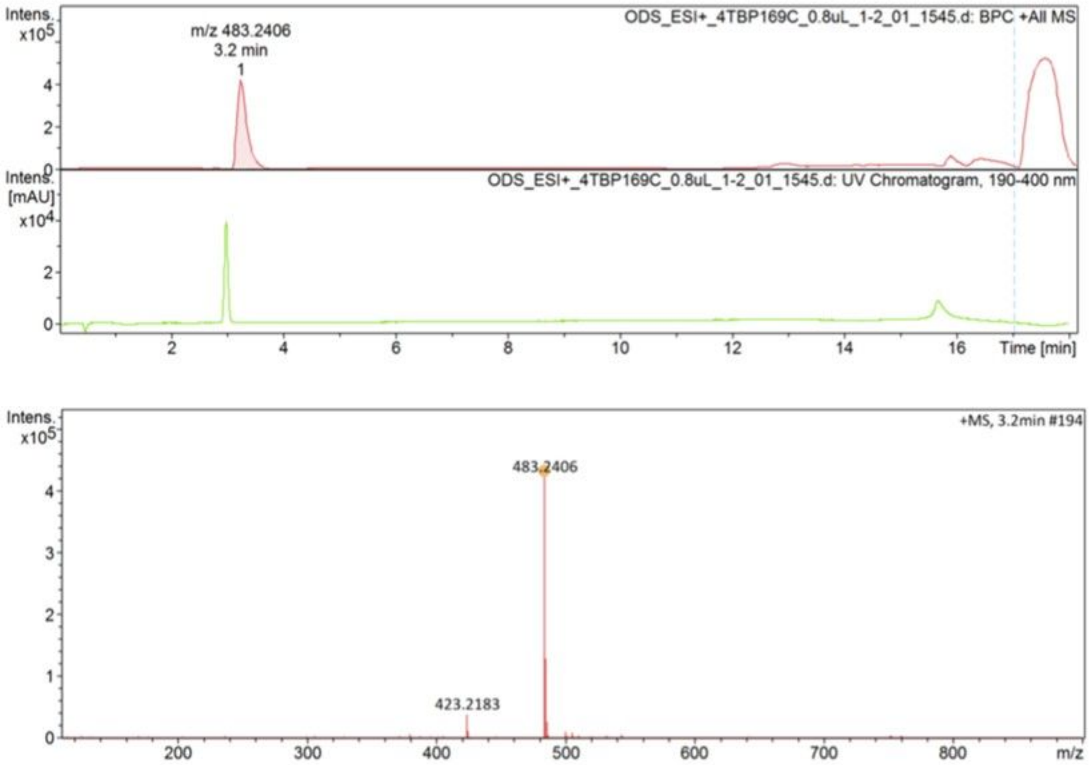

Figure S26: UFLC-PDA-ESI-(+)-HRMS data for 7-*epi*-gedunin (**5**).

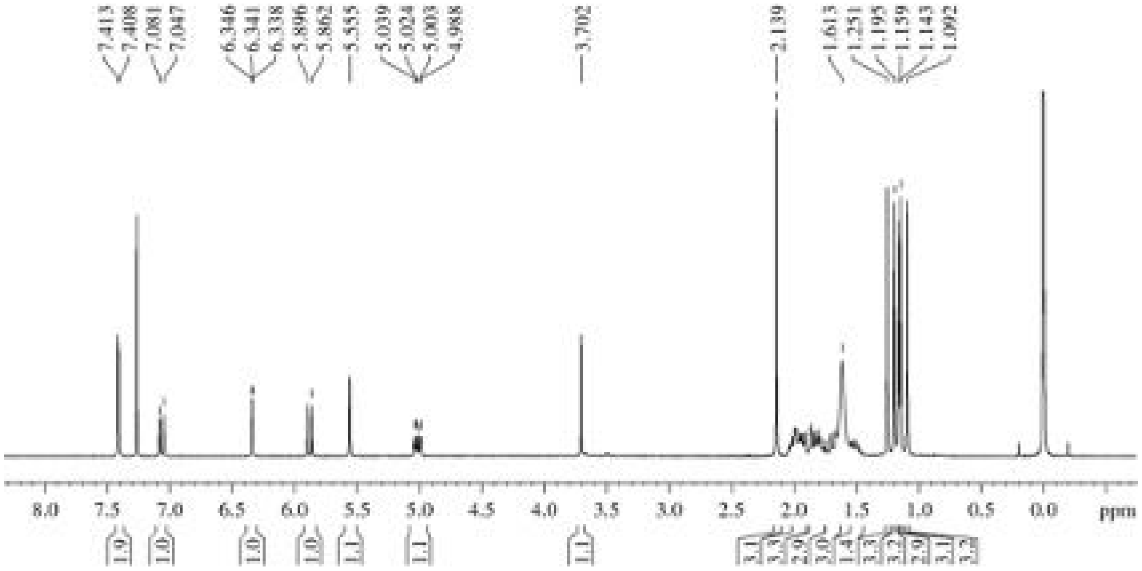

Figure S27: ^{1}H NMR ($CDCl_3$, 300 MHz) spectrum of 7-*epi*-gedunin (**5**).

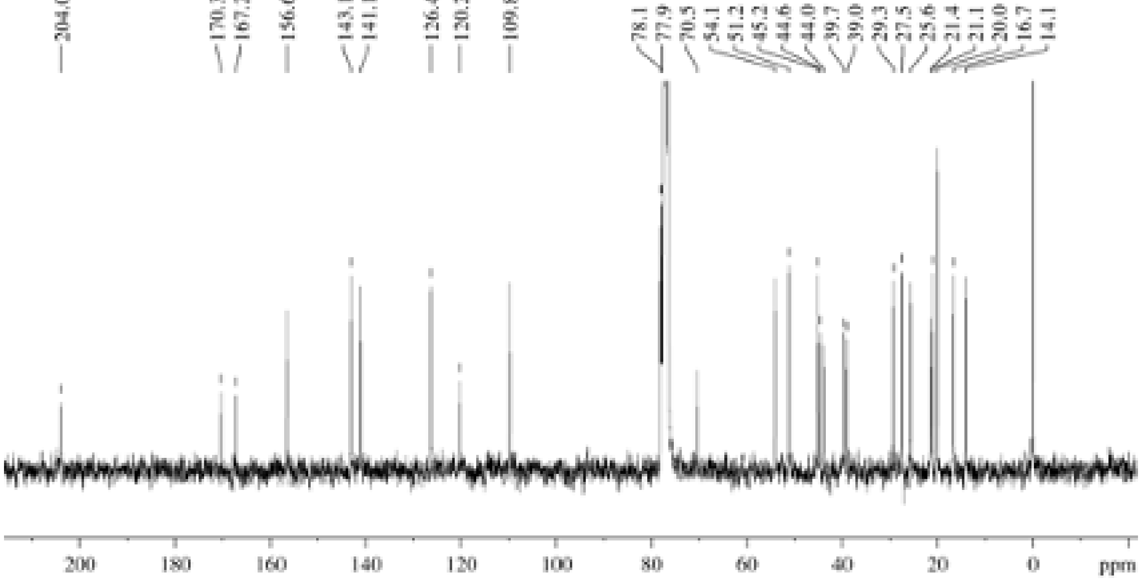

Figure S28: ^{13}C NMR ($CDCl_3$, 75 MHz) spectrum of 7-*epi*-gedunin (**5**).

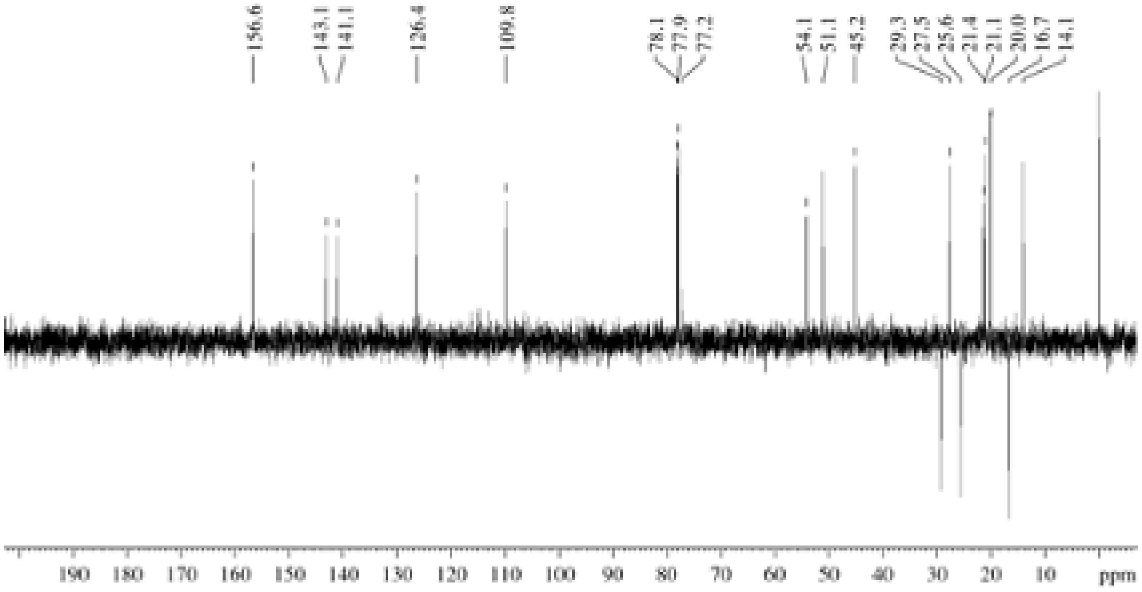

Figure S29: DEPT 135º ($CDCl_3$, 75 MHz) spectrum of 7-*epi*-gedunin (**5**).

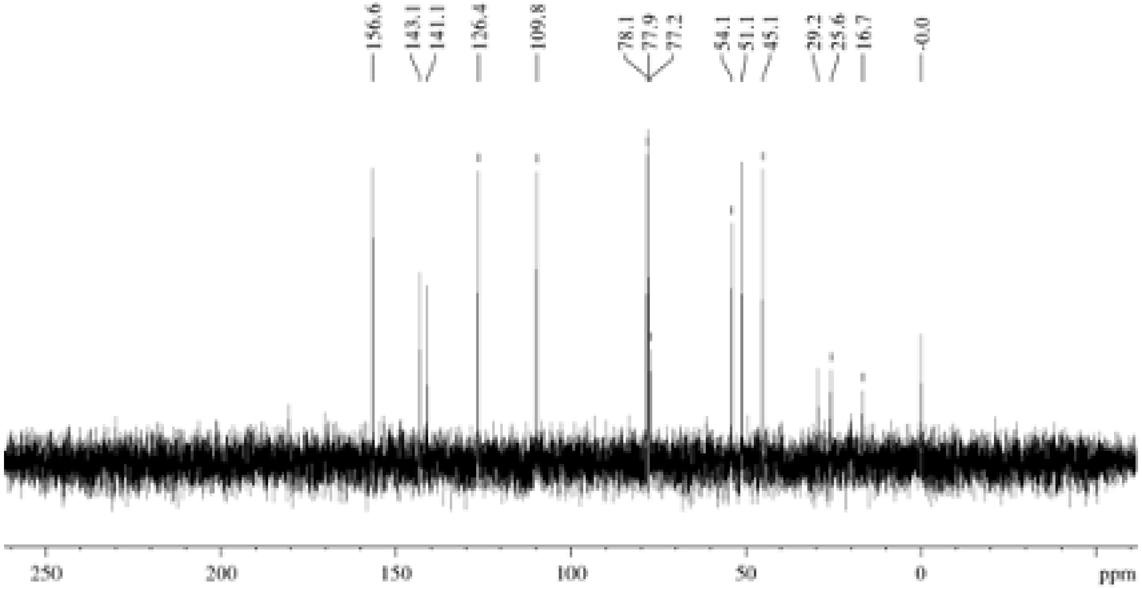

Figure S30: DEPT 90º ($CDCl_3$, 75 MHz) spectrum of 7-*epi*-gedunin (**5**).

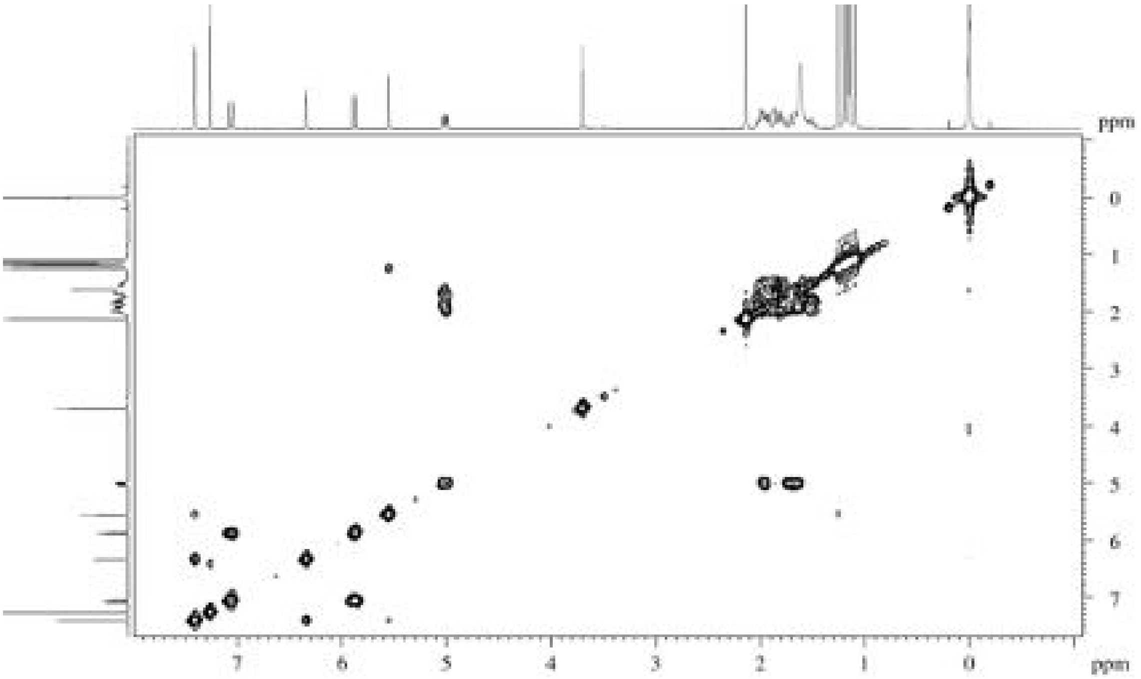

Figure S31: COSY ($CDCl_3$, 300 MHz) spectrum of 7-*epi*-gedunin (**5**).

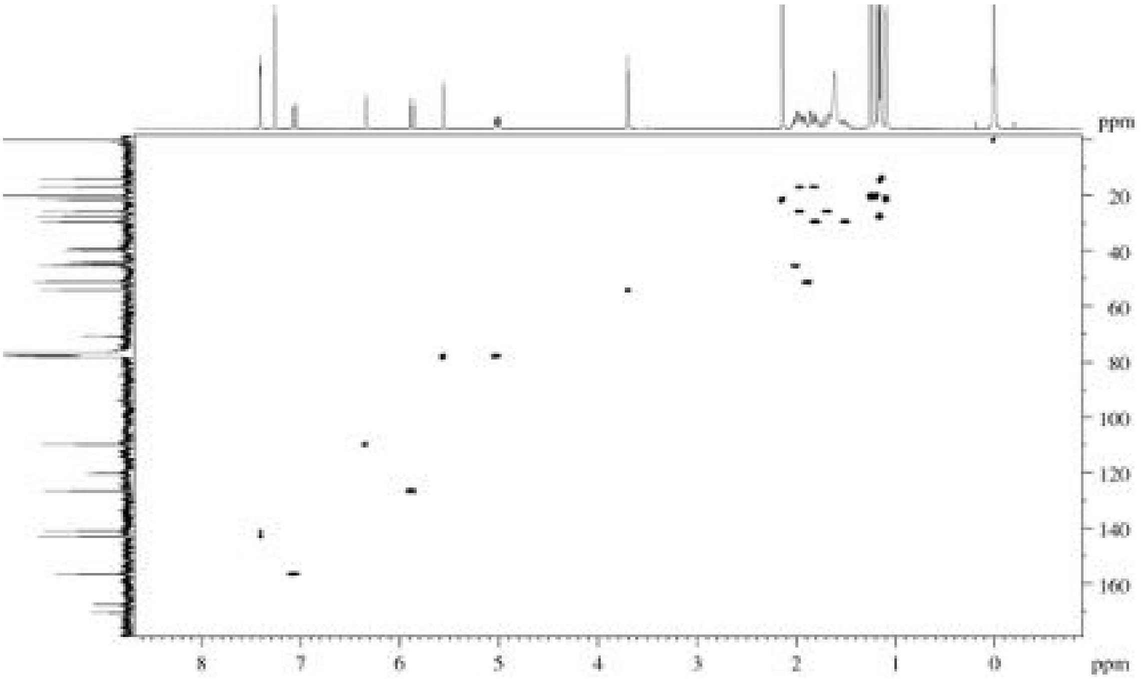

Figure S32: HSQC ($CDCl_3$, 300; 75 MHz) spectrum of 7-*epi*-gedunin (**5**).

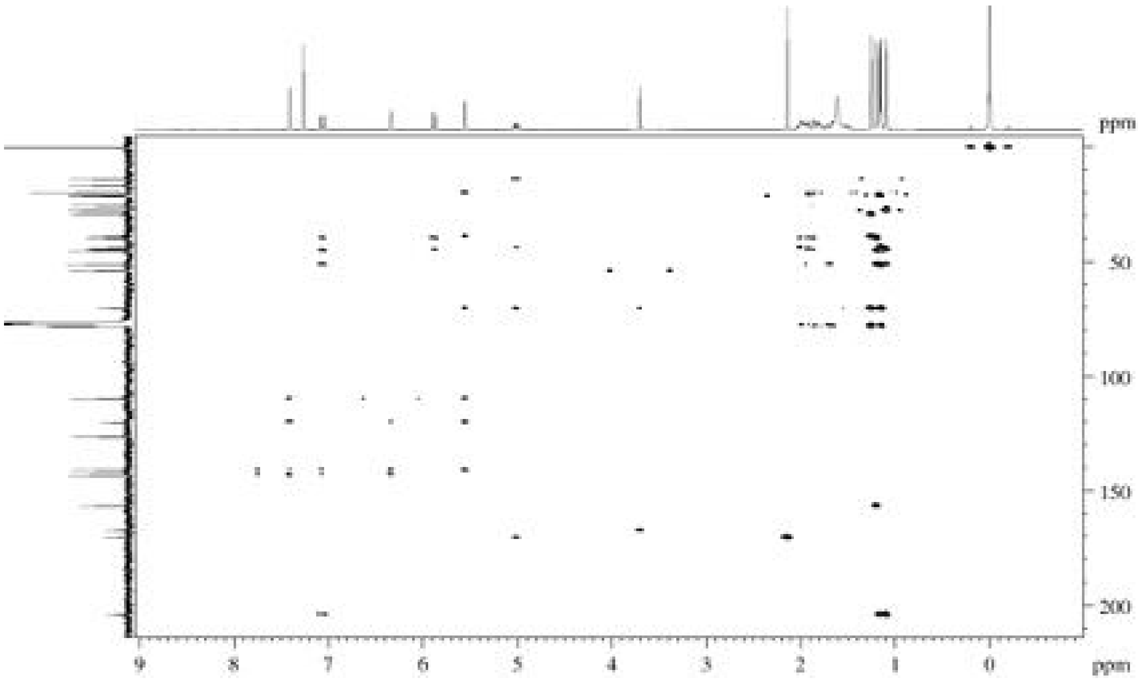

Figure S33: HMBC ($CDCl_3$, 300; 75 MHz) spectrum of 7-*epi*-gedunin (**5**).

7-Deacetyl-7α-butanoyloxygedunin (6):

^{1}H NMR ($CDCl_3$, 300 MHz): δ 7.42 (2H, *m*, H21 and H23), 7.11 (1H, *d*, *J* = 10.2 Hz, H1), 6.35 (1H, *t*, *J* = 1.2 Hz, H22), 5.87 (1H, *d*, *J* = 10.2 Hz, H2), 5.61 (1H, *s*, H17), 4.58 (1H, *dd*, *J* = 3.0, 1.8 Hz, H7), 3.53 (1H, *s*, H15), 2.50 (1H, *dd*, *J* = 12.3, 5.7 Hz, H9), 2.33 (2H, *m*, $CH_3CH_2C\underline{H_2}CO$), 2.17 (1H, *dd*, *J* = 13.2, 2.4 Hz, H5), 1.98* (*m*, H11α), 1.94* (*m*, H6α), 1.86* (*m*, H11β), 1.83* (*m*, H6β), 1.78* (*m*, H12α), 1.62* (*m*, $CH_3C\underline{H_2}CH_2CO$), 1.61* (*m*, H12β), 1.25 (3H, *s*, H18), 1.23 (3H, *s*, H29), 1.16 (3H, *s*, H30), 1.08 (3H, *s*, H19), 1.05 (3H, *s*, H28), 1.00 (3H, *t*, *J* = 7.0 H, $C\underline{H_3}CH_2CH_2CO$). Note: *chemical shift ascertained from the HSQC spectrum. ^{13}C NMR ($CDCl_3$, 75 MHz): δ 204.0 (*s*, C3), 172.6 (*s*, $CH_3CH_2CH_2\underline{C}O$), 167.4 (*s*, C16), 157.1 (*d*, C1), 143.1 (*d*, C23), 141.2 (*d*, C21), 126.0 (*d*, C2), 120.4 (*s*, C20), 109.9 (*d*, C22), 78.2 (*d*, C17), 72.8 (*d*, C7), 69.7 (*s*, C14), 57.0 (*d*, C15), 46.0 (*d*, C5), 44.1 (*s*, C4), 42.6 (*s*, C8), 40.1 (*s*, C10), 39.5 (*d*, C9), 38.7 (*s*, C13), 36.4 (*t*, $CH_3CH_2\underline{C}H_2CO$), 27.2 (*q*, C28), 25.9 (*t*, C12), 23.3 (*t*, C6),

21.2 (*q*, C19), 19.8 (*q*, C29), 18.4 (*q*, C30), 18.3 (*t*, $CH_3\underline{C}H_2CH_2CO$), 17.7 (*q*, C18), 15.0 (*t*, C11), 13.8 (*q*, $\underline{C}H_3CH_2CH_2CO$).

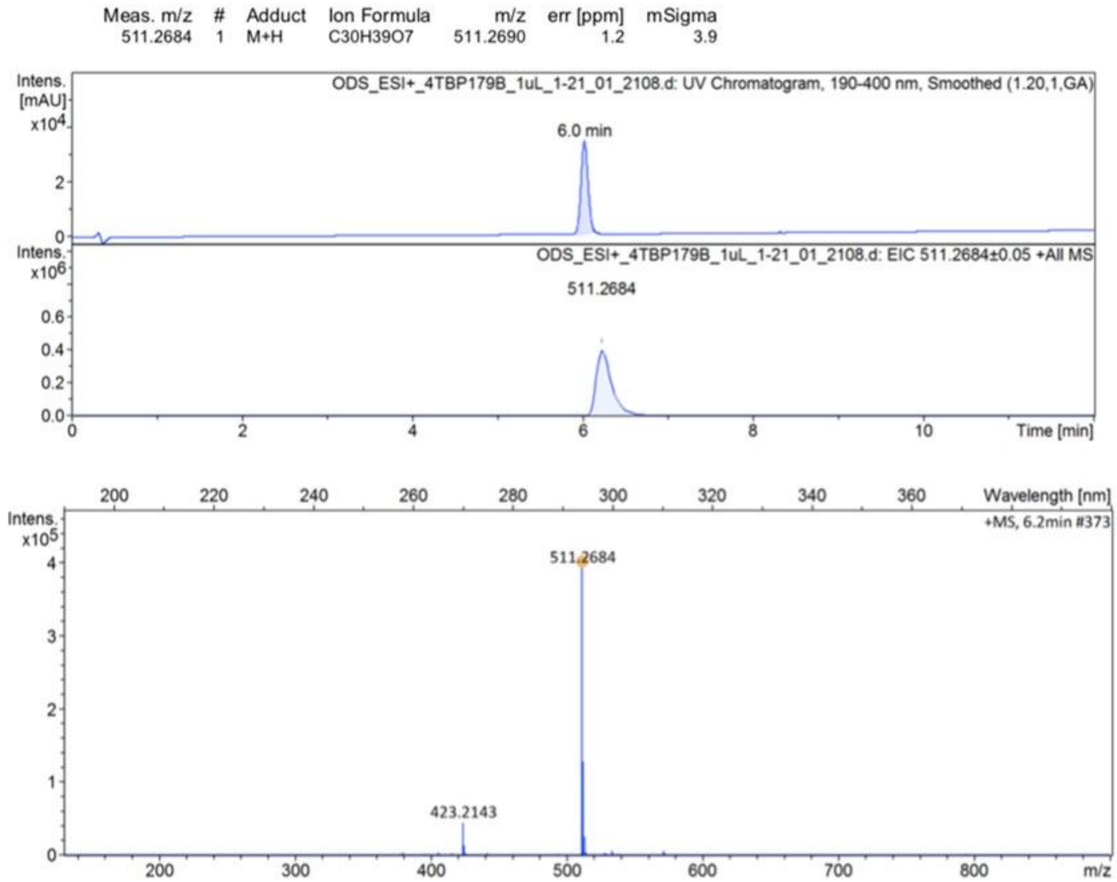

Figure S34: UFLC-PDA-ESI-(+)-HRMS data for 7α-butanoyloxygedunin **6**.

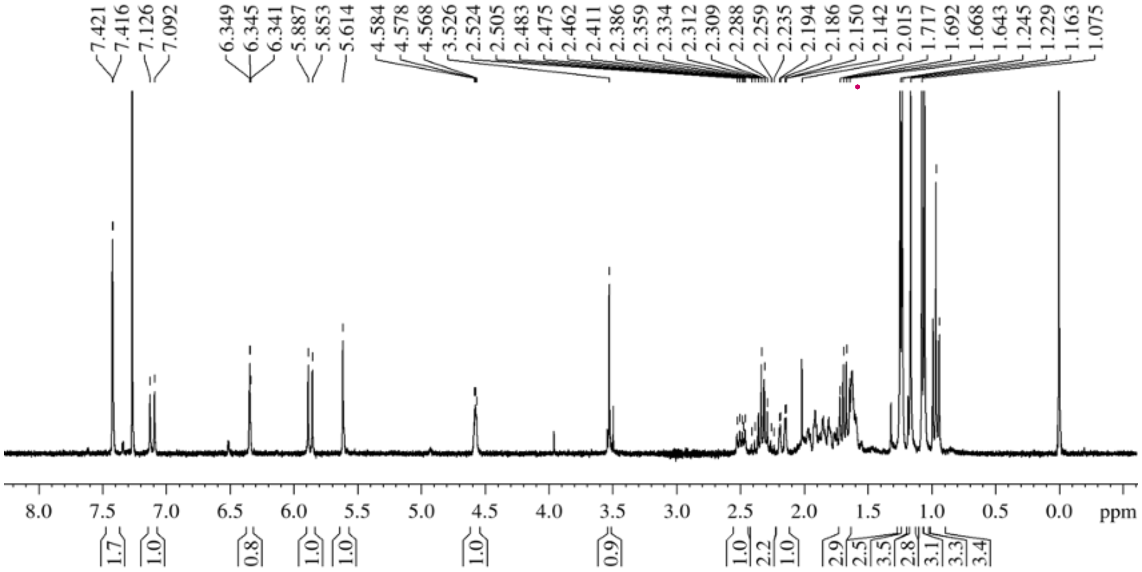

Figure S35: ^1H NMR ($CDCl_3$, 300 MHz) spectrum of 7α-butanoyloxygedunin **6**.

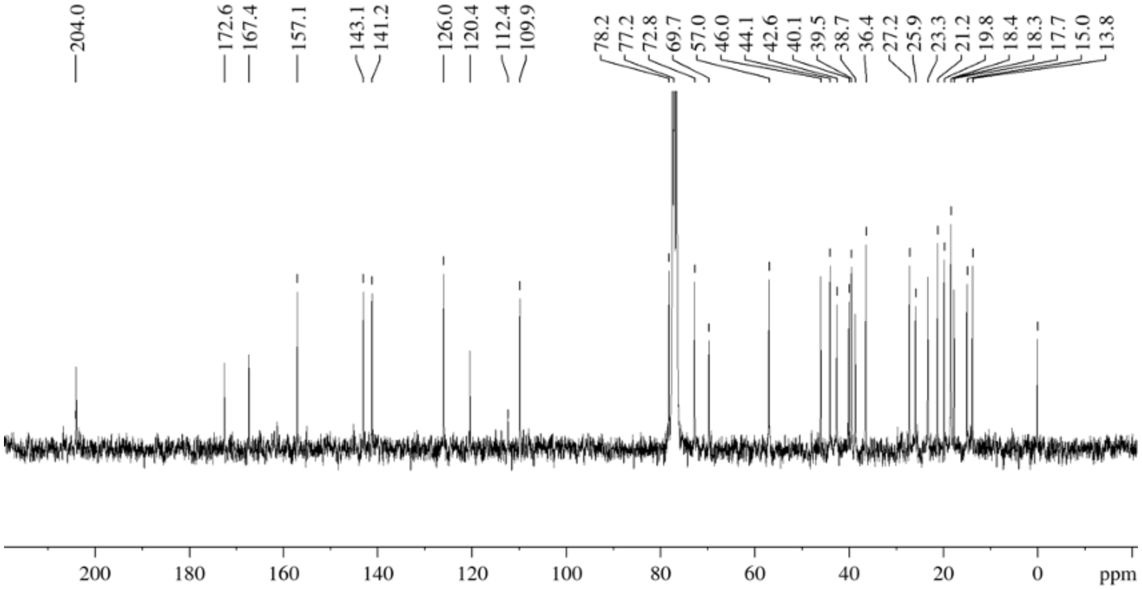

Figure S36: ^{13}C NMR ($CDCl_3$, 75 MHz) spectrum of 7α-butanoyloxygedunin **6**.

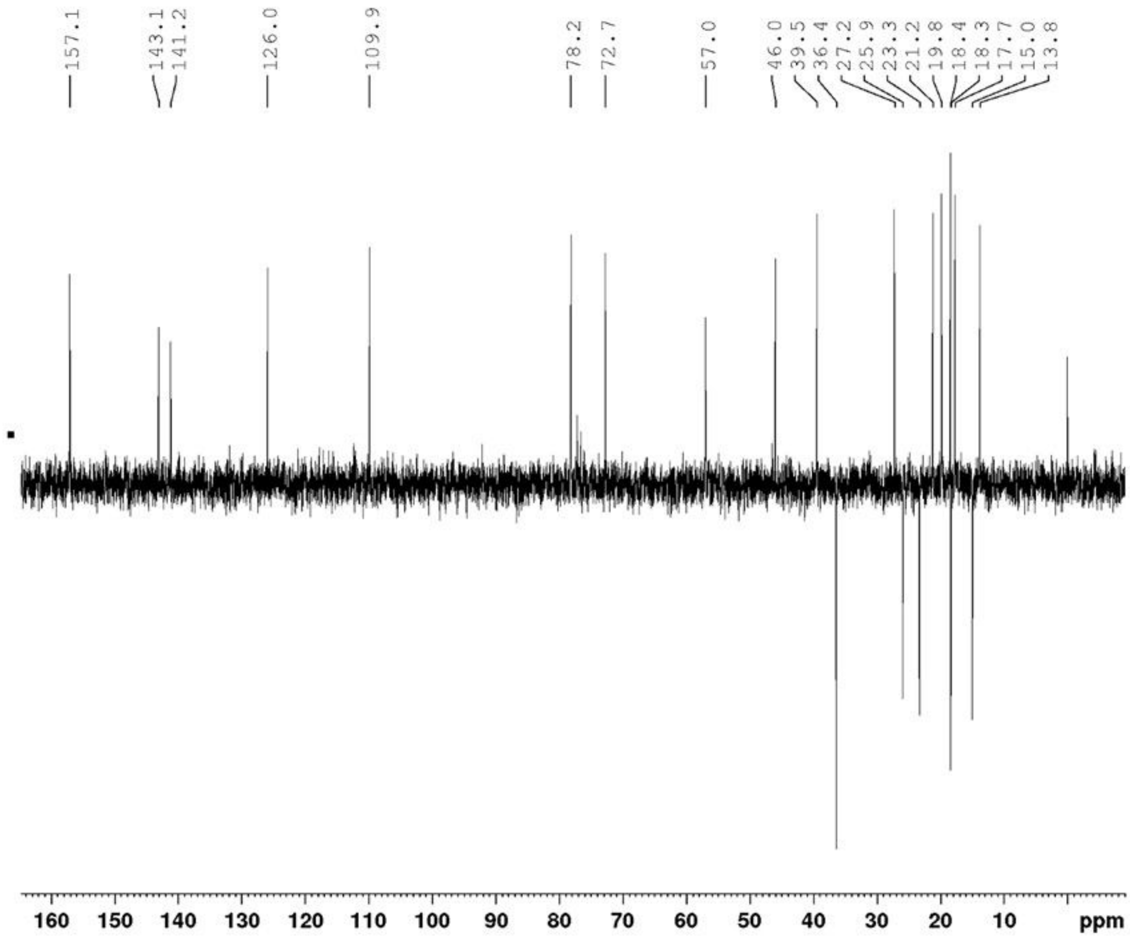

Figure S37: DEPT 135° ($CDCl_3$, 75 MHz) spectrum of 7α-butanoyloxygedunin **6**.

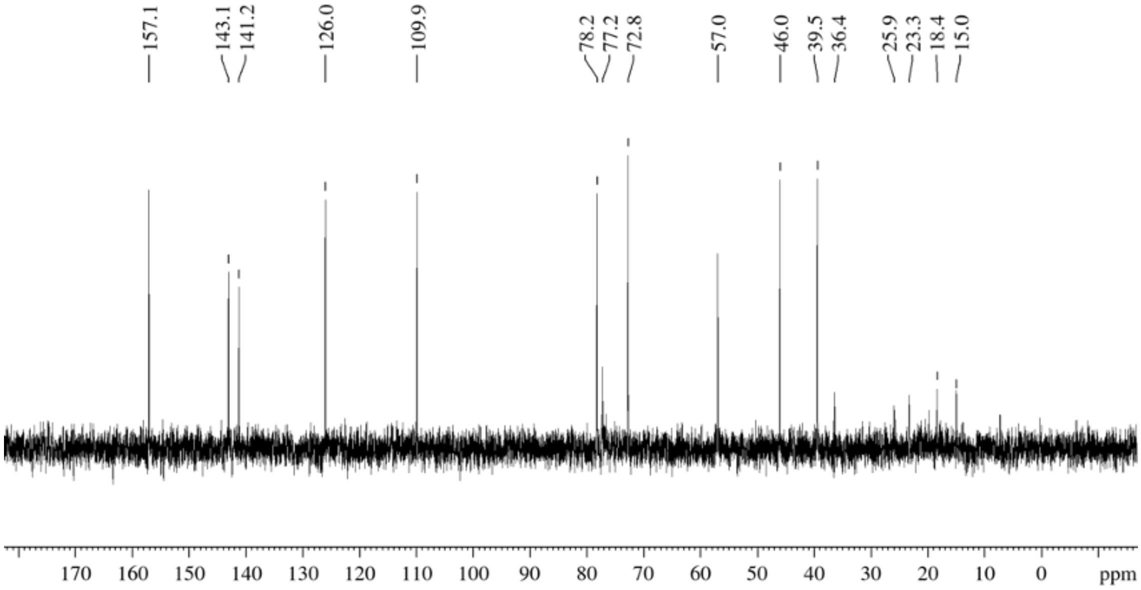

Figure S38: DEPT 90º ($CDCl_3$, 75 MHz) spectrum of 7α-butanoyloxygedunin **6**.

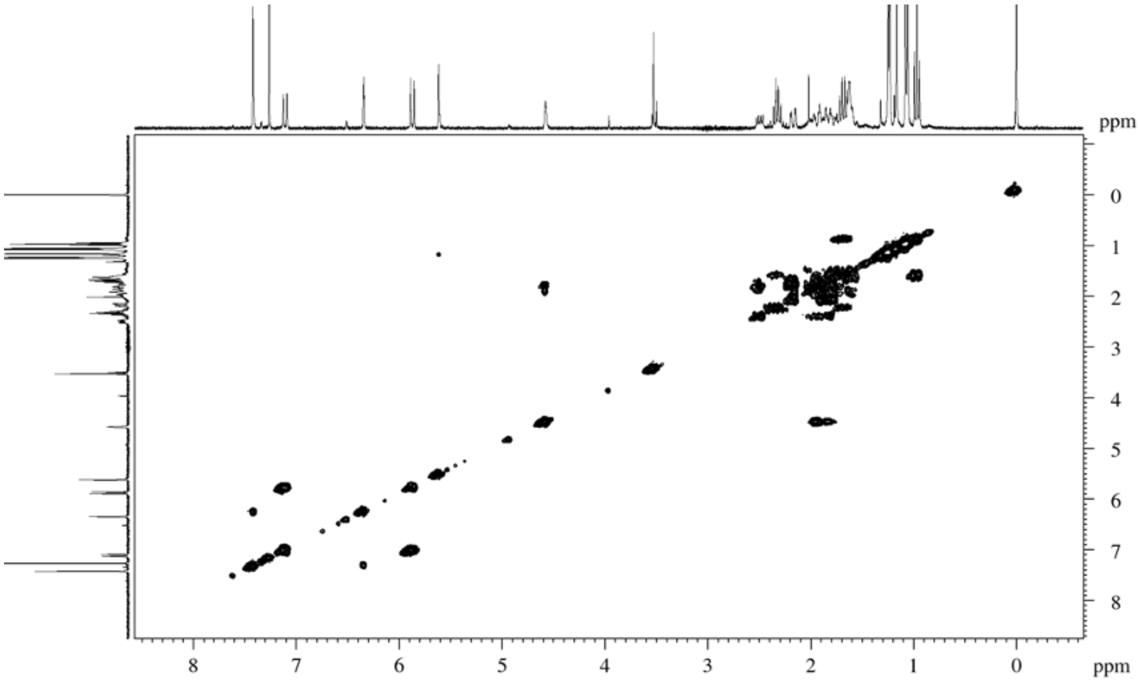

Figure S39: COSY ($CDCl_3$, 300 MHz) spectrum 7α-butanoyloxygedunin **6**.

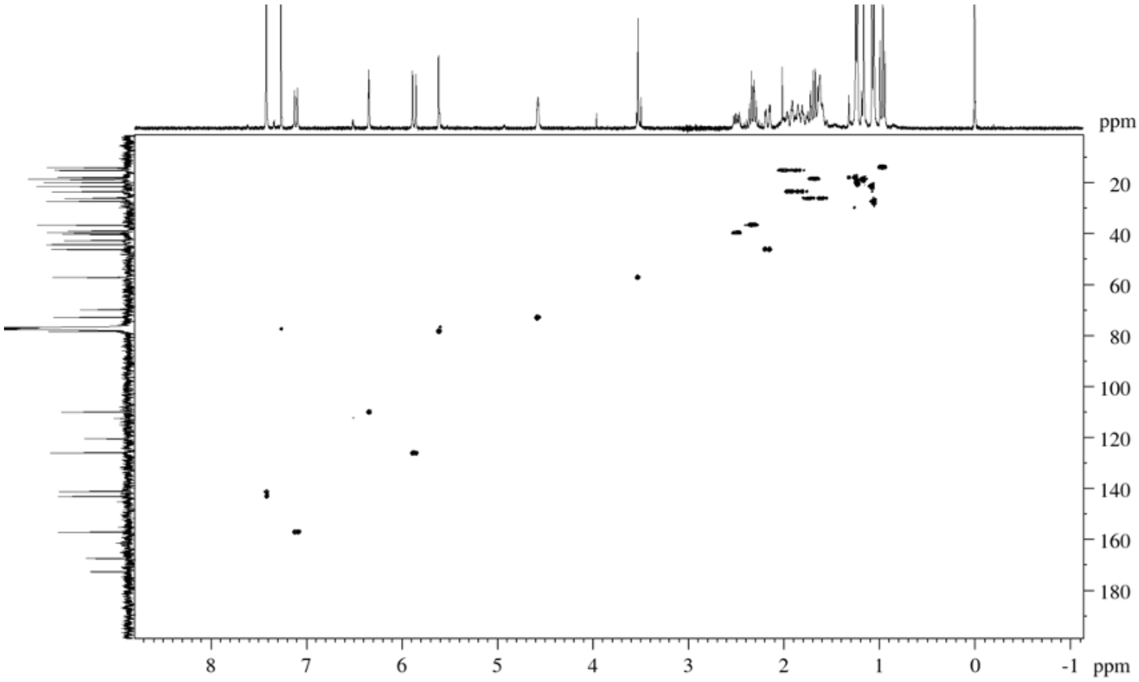

Figure S40: HSQC ($CDCl_3$, 300; 75 MHz) spectrum of 7α-butanoyloxygedunin **6**.

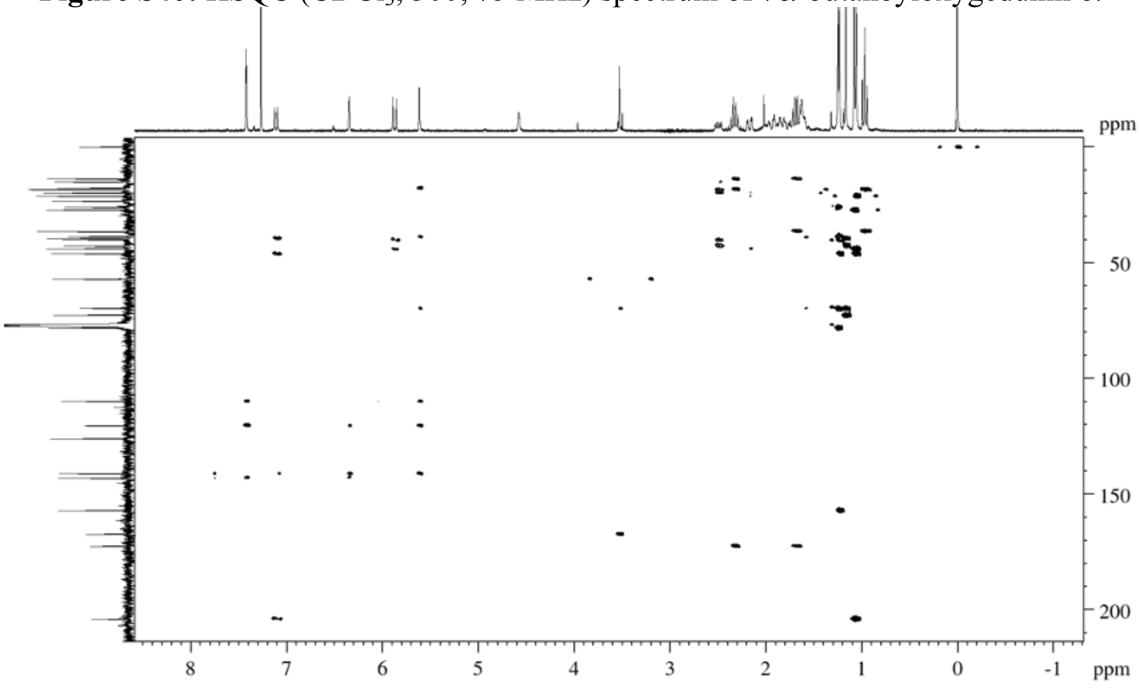

Figure S41: HMBC ($CDCl_3$, 300; 75 MHz) spectrum of 7α-butanoyloxygedunin **6**.

7-Deacetyl-7β-butanoyloxygedunin (7):

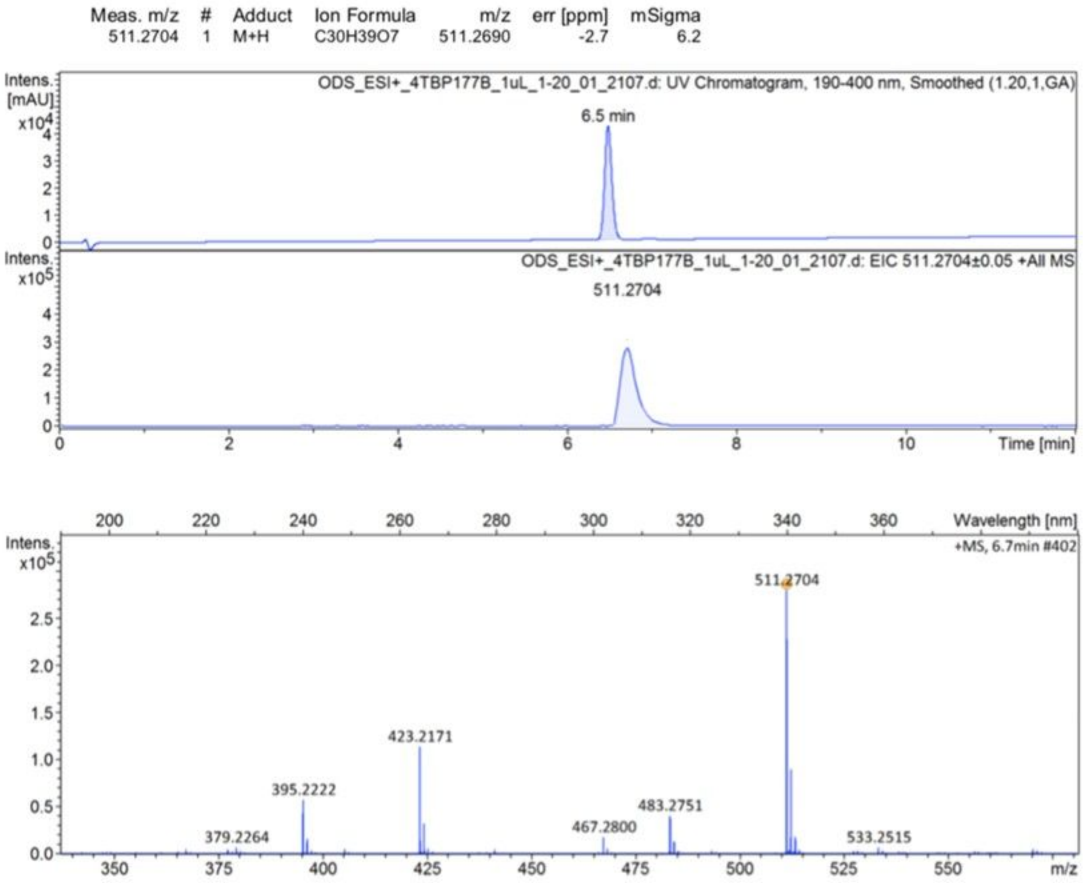

Figure S42: UFLC-PDA-ESI-(+)-HRMS data for new 7β-butanoyloxygedunin **7**.

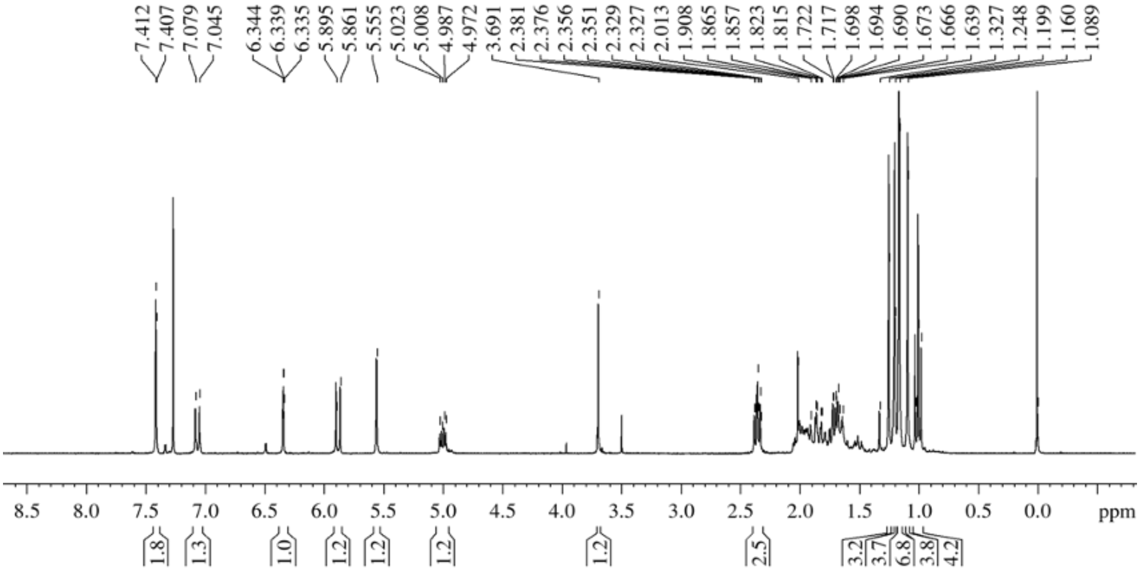

Figure S43: ^{1}H NMR ($CDCl_3$, 300 MHz) spectrum of new 7β-butanoyloxygedunin **7**.

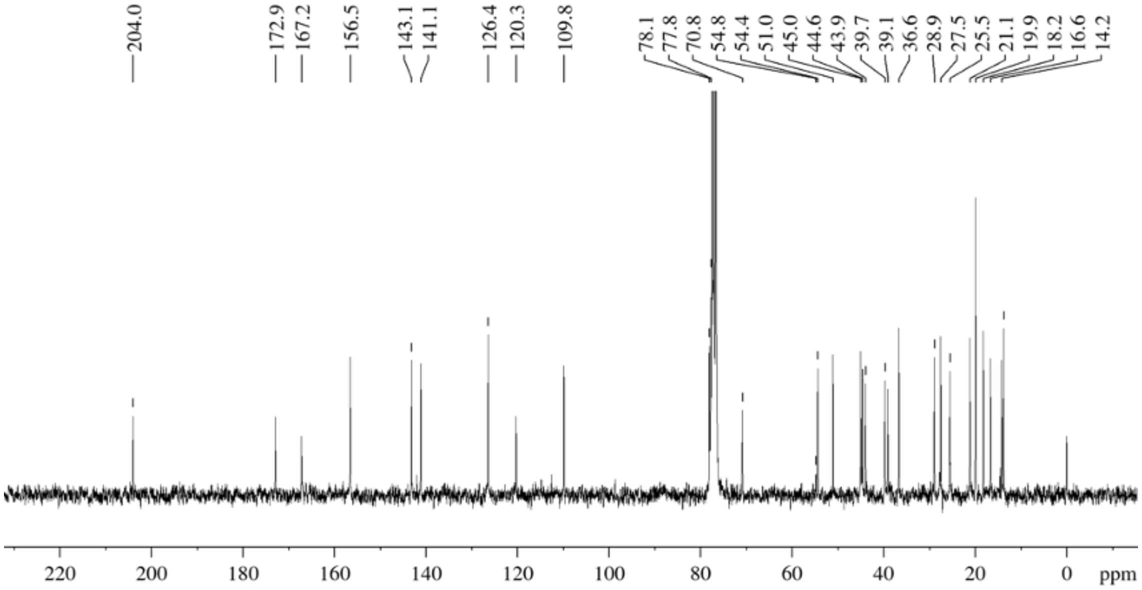

Figure S44: ^{13}C NMR ($CDCl_3$, 75 MHz) spectrum of new 7β-butanoyloxygedunin **7**.

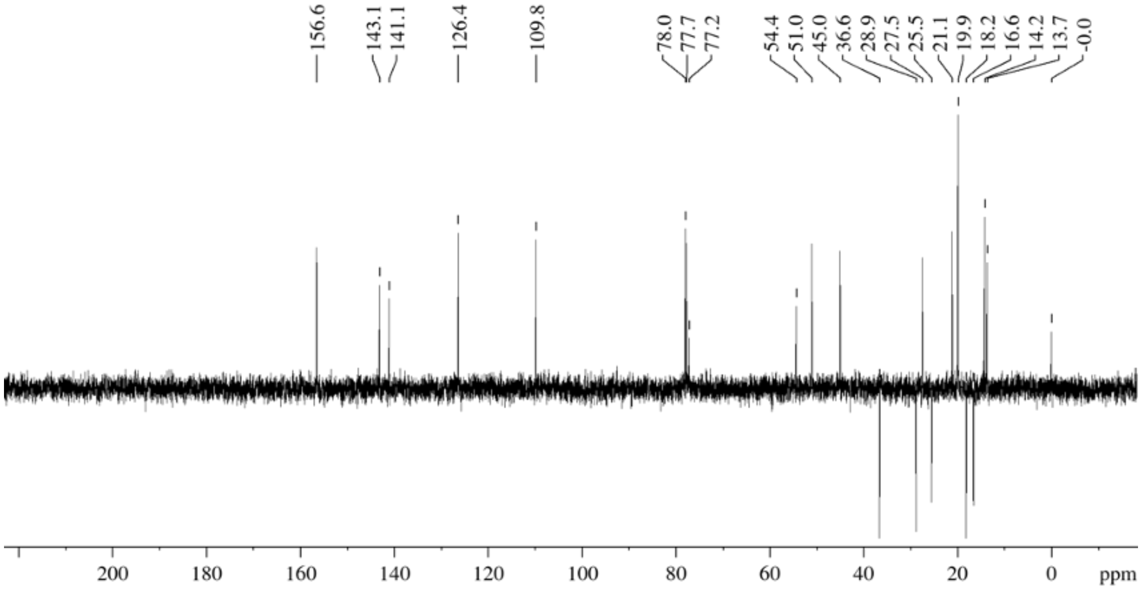

Figure S45: DEPT 135º ($CDCl_3$, 75 MHz) spectrum of new 7β-butanoyloxygedunin **7**.

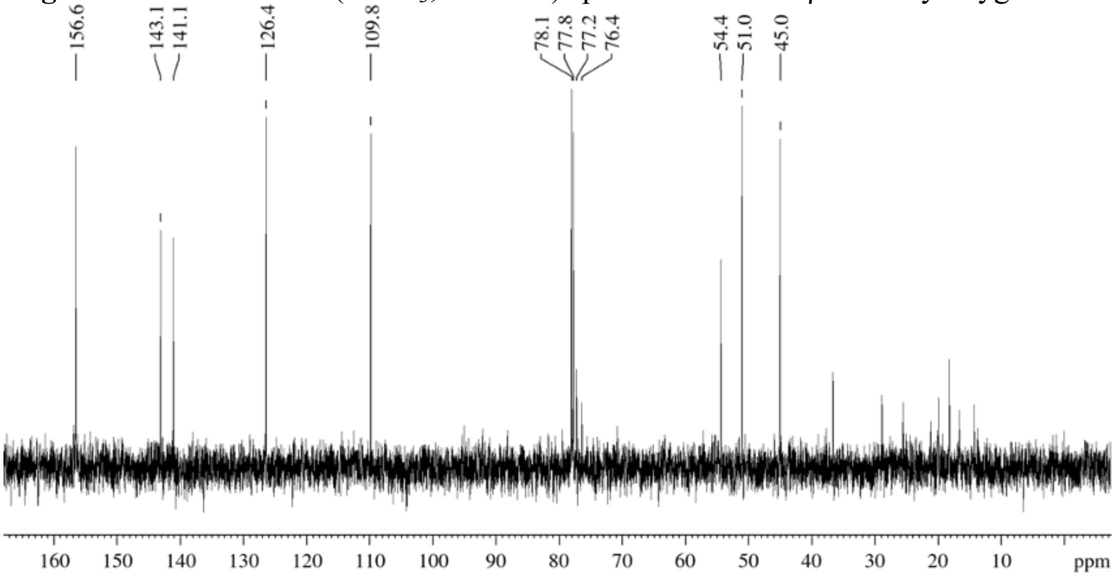

Figure S46: DEPT 90º ($CDCl_3$, 75 MHz) spectrum of new 7β-butanoyloxygedunin **7**.

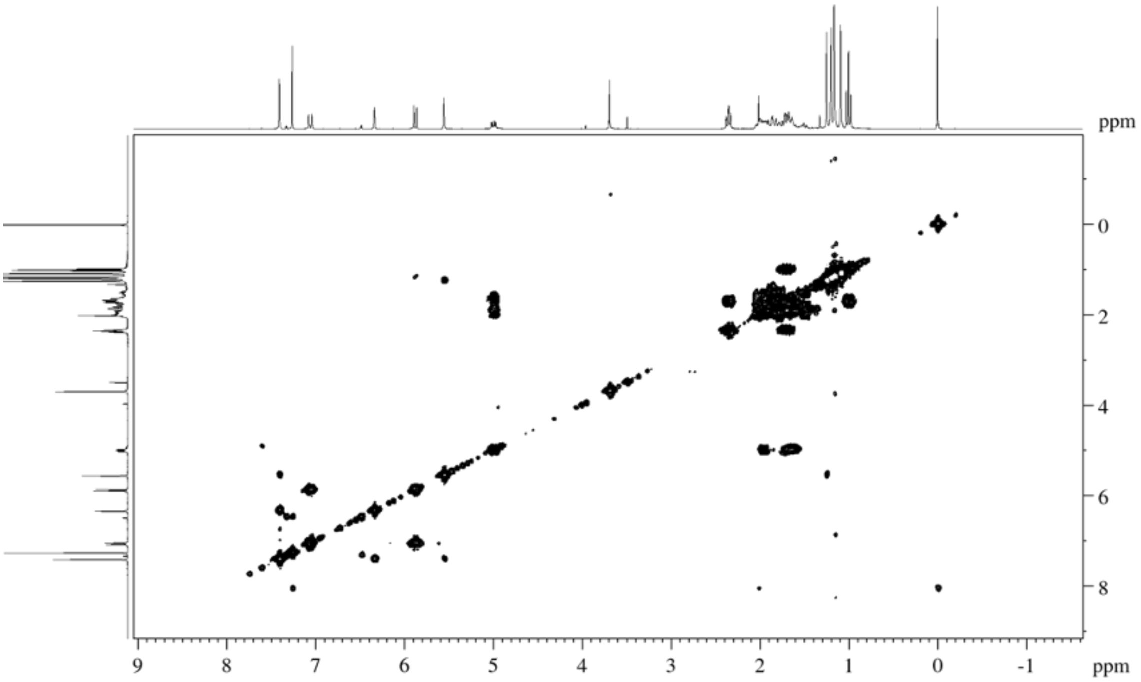

Figure S47: COSY ($CDCl_3$, 300 MHz) spectrum of new 7β-butanoyloxygedunin **7**.

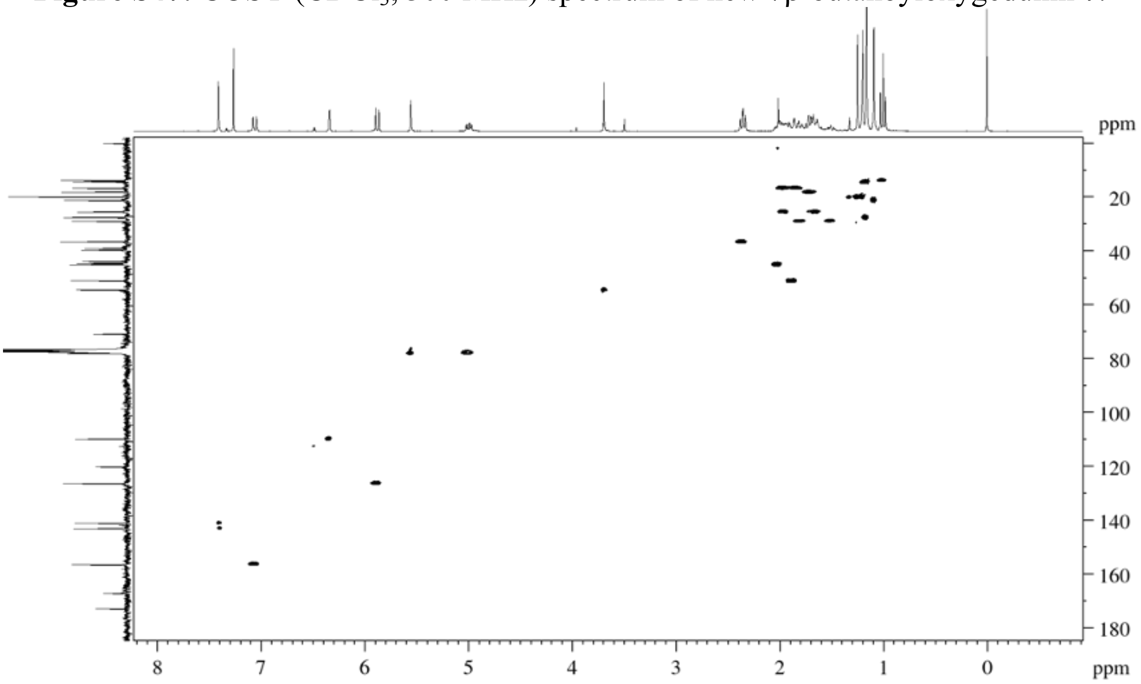

Figure S48: HSQC ($CDCl_3$, 300; 75 MHz) spectrum of new 7β-butanoyloxygedunin **7**.

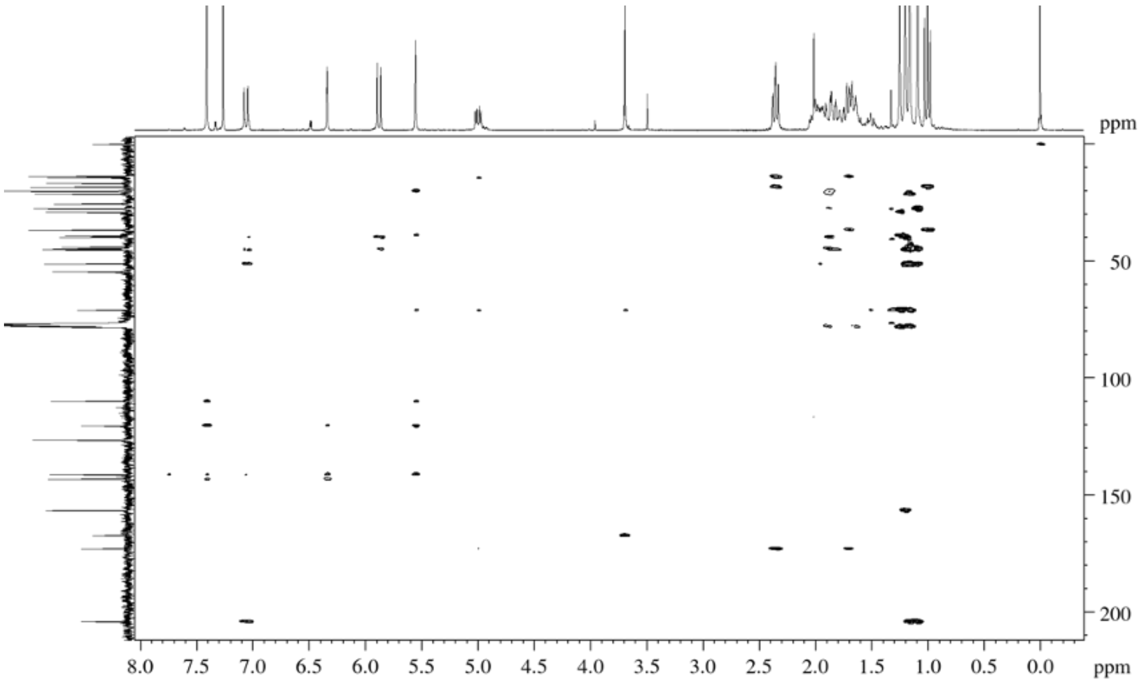

Figure S49: HMBC ($CDCl_3$, 300; 75 MHz) spectrum of new 7β-butanoyloxygedunin **7**.

7-Deacetyl-7α-pentanoyloxygedunin (8):

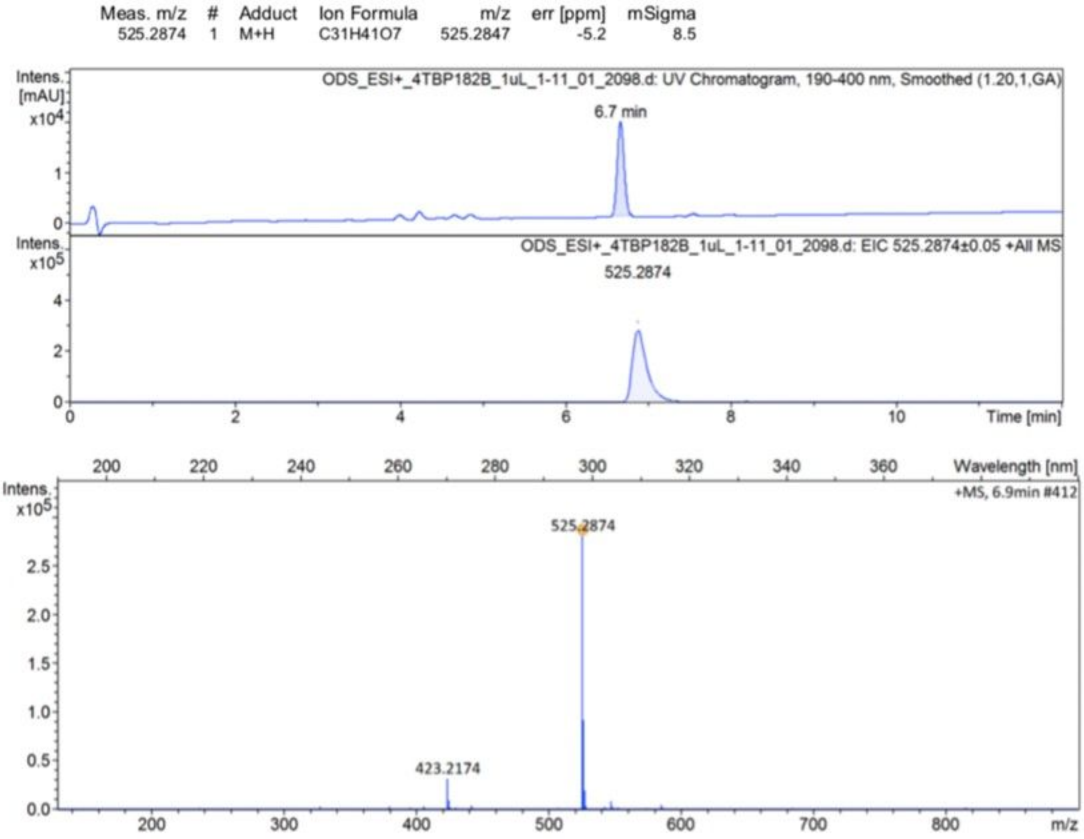

Figure S50: UFLC-PDA-ESI-(+)-HRMS data for new 7α-pentanoyloxygedunin **8**.

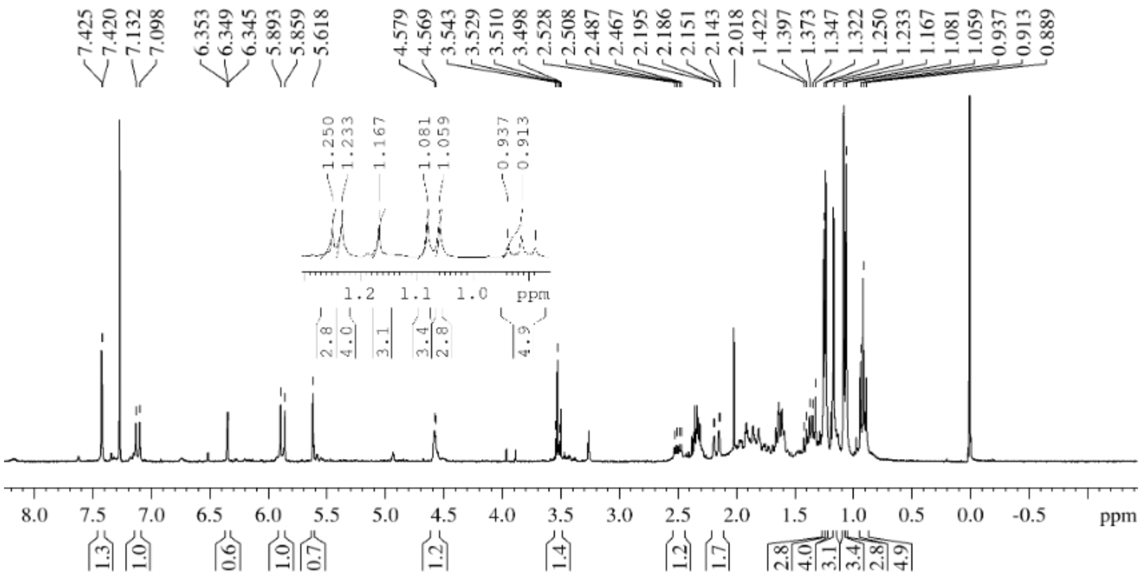

Figure S51: ^{1}H NMR ($CDCl_3$, 300 MHz) spectrum of new 7α-pentanoyloxygedunin **8**.

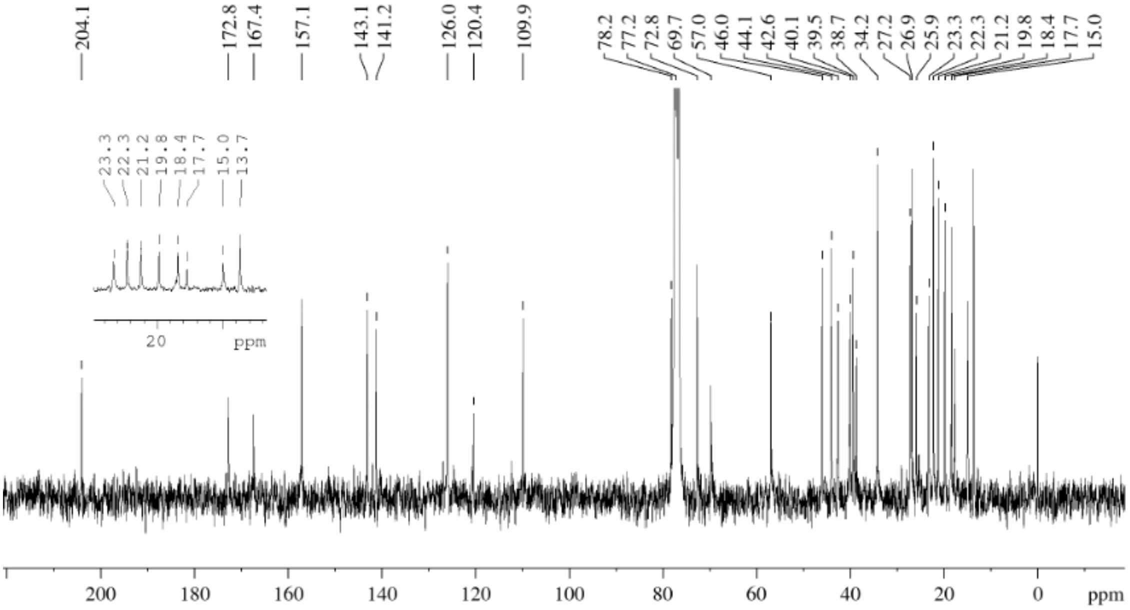

Figure S52: ^{13}C NMR ($CDCl_3$, 75 MHz) spectrum of new 7α-pentanoyloxygedunin **8**.

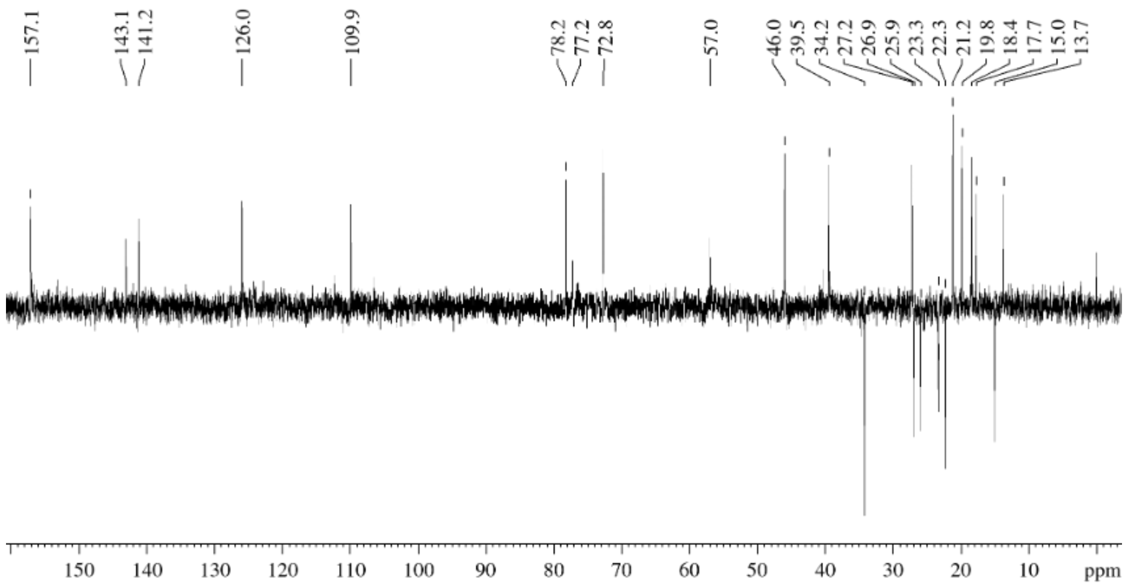

Figure S53: DEPT 135º ($CDCl_3$, 75 MHz) spectrum of new 7α-pentanoyloxygedunin **8**.

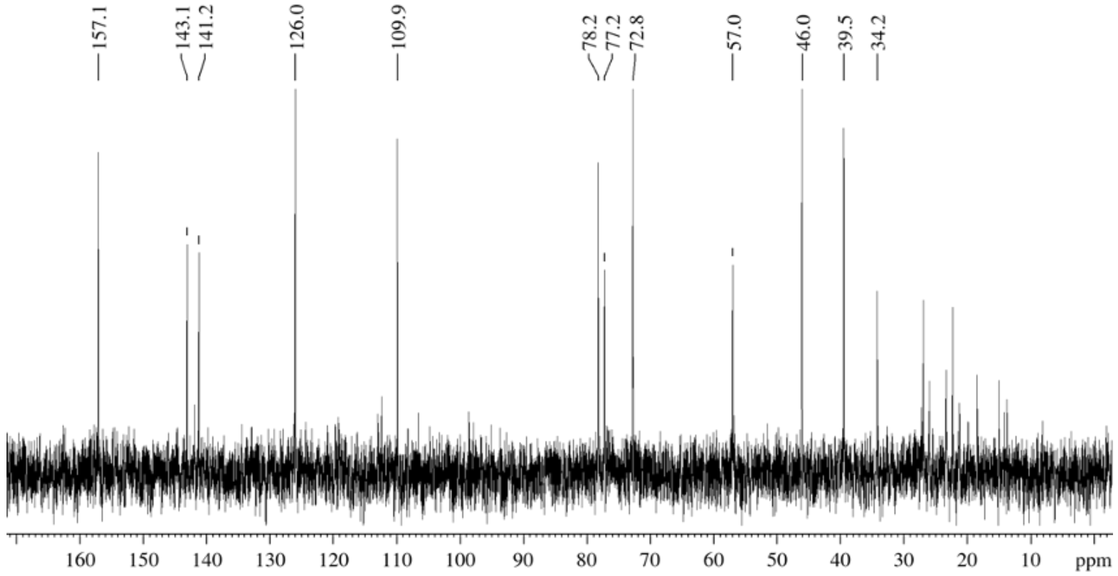

Figure S54: DEPT 90º ($CDCl_3$, 75 MHz) spectrum of new 7α-pentanoyloxygedunin **8**.

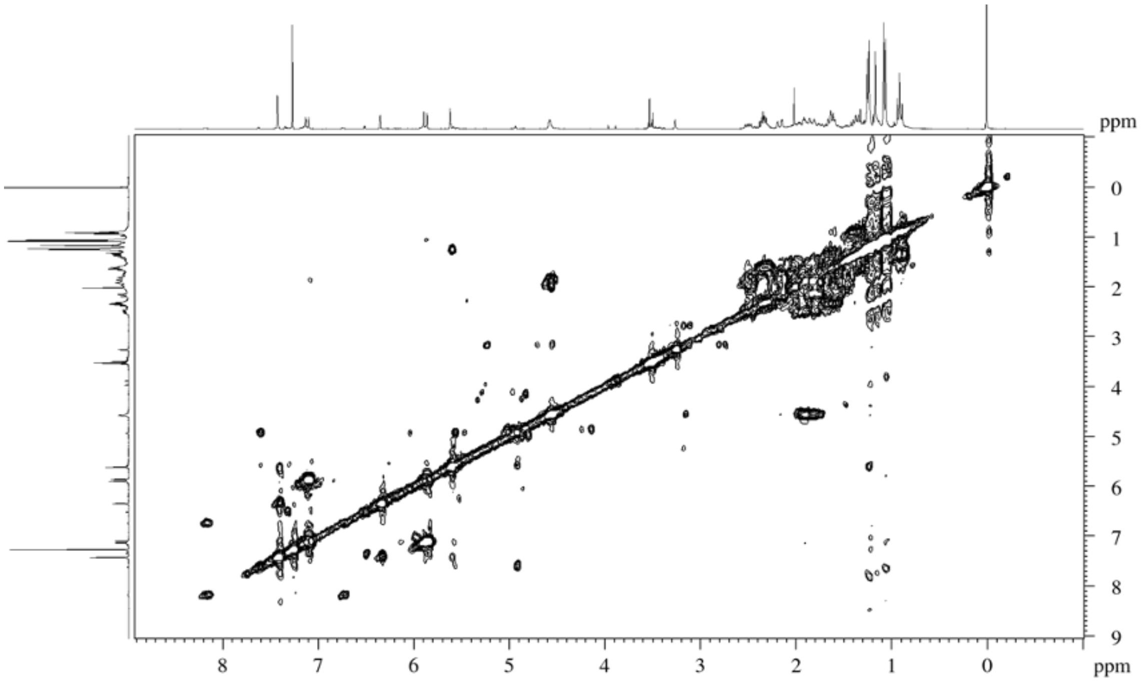

Figure S55: COSY ($CDCl_3$, 300 MHz) spectrum of new 7α-pentanoyloxygedunin **8**.

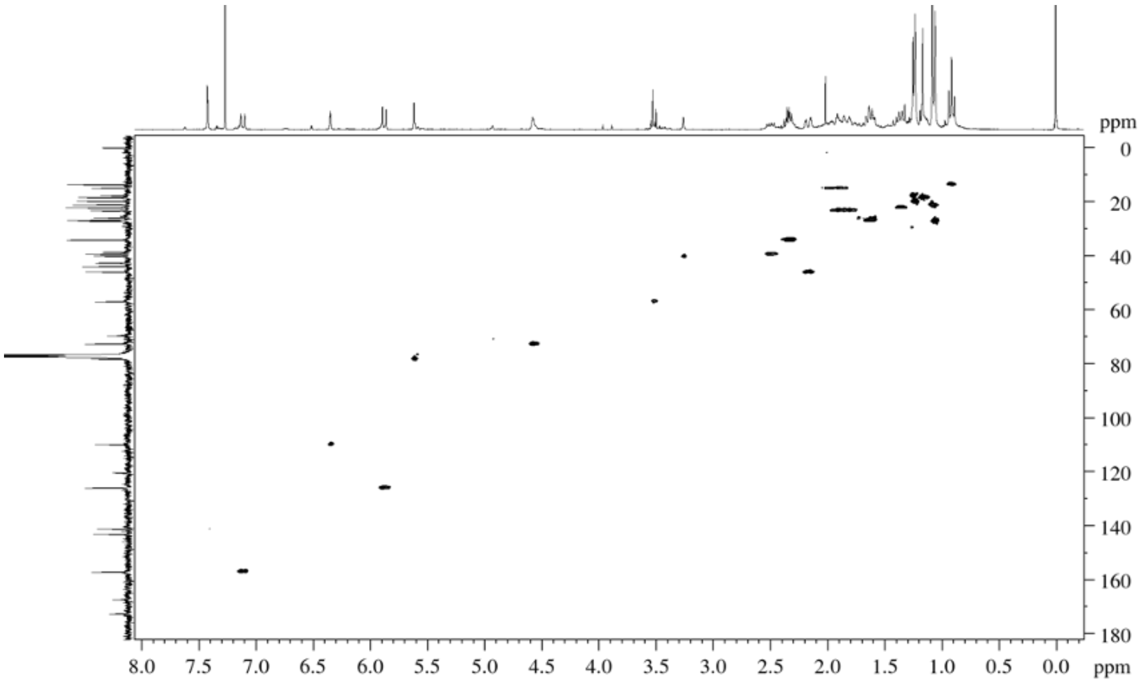

Figure S56: HSQC ($CDCl_3$, 300; 75 MHz) spectrum of new 7α-pentanoyloxygedunin **8**.

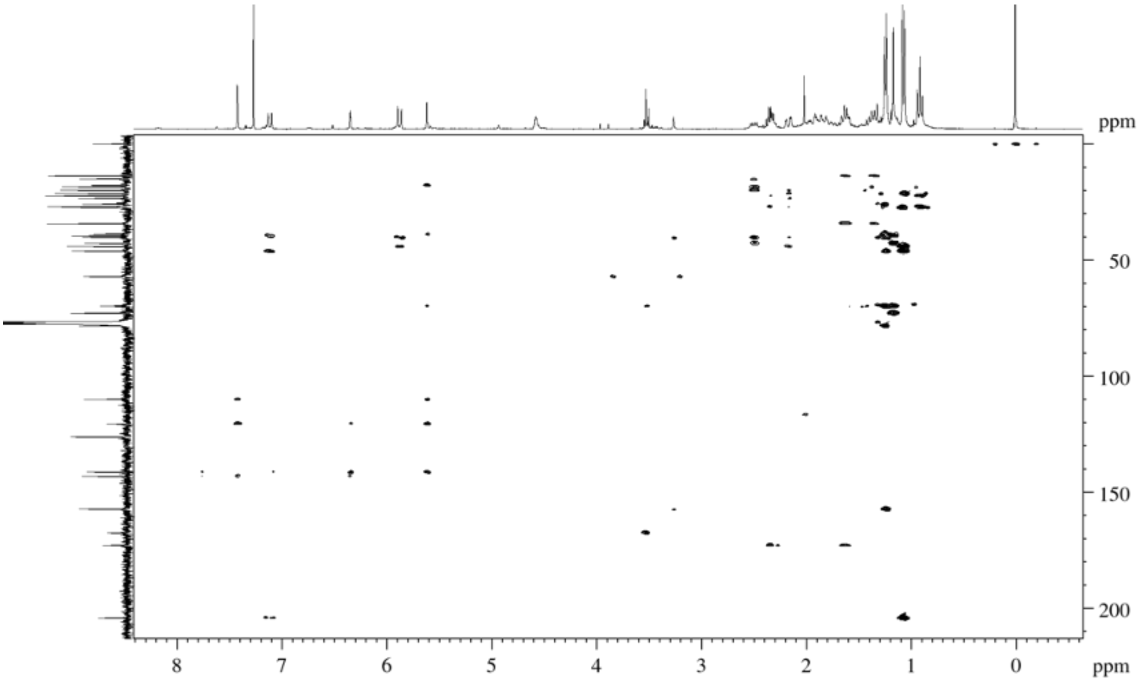

Figure S57: HMBC ($CDCl_3$, 300; 75 MHz) spectrum of new 7α-pentanoyloxygedunin **8**.

7-Deacetyl-7β-pentanoyloxygedunin (9):

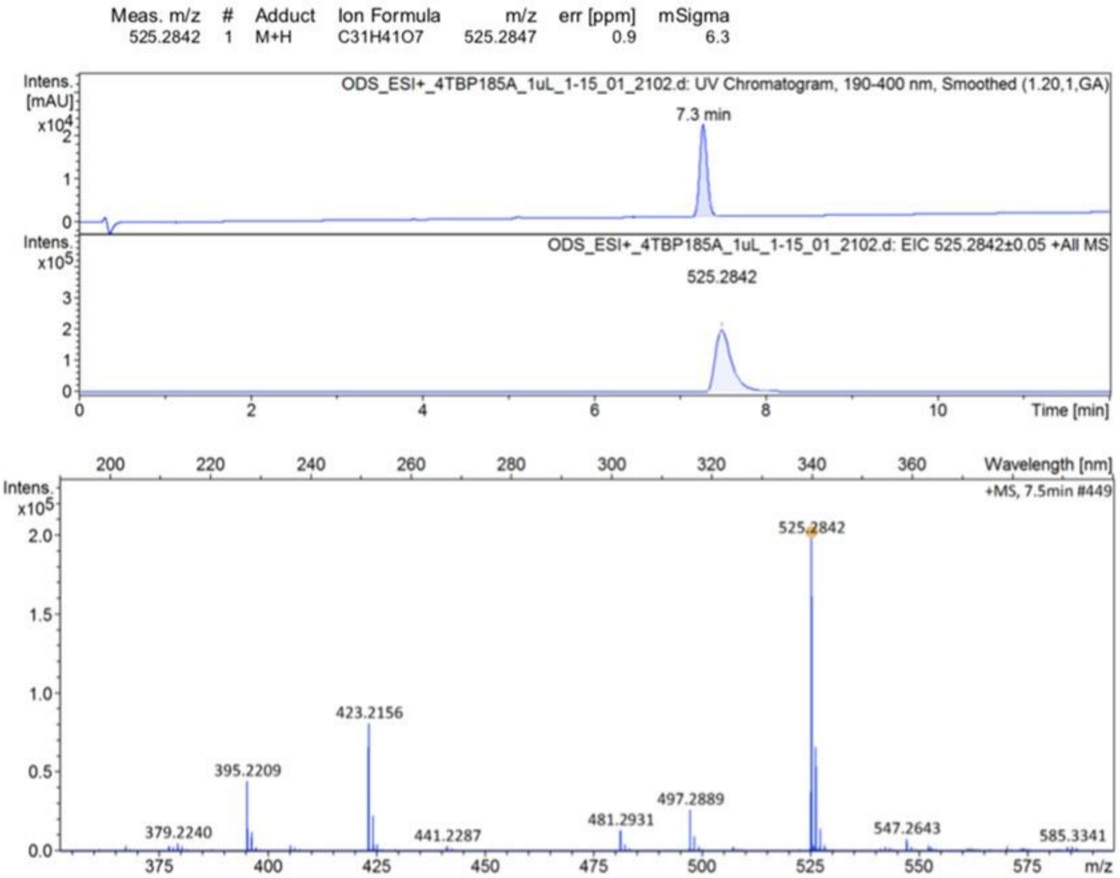

Figure S58: UFLC-PDA-ESI-(+)-HRMS data for new 7β-pentanoyloxygedunin **9**.

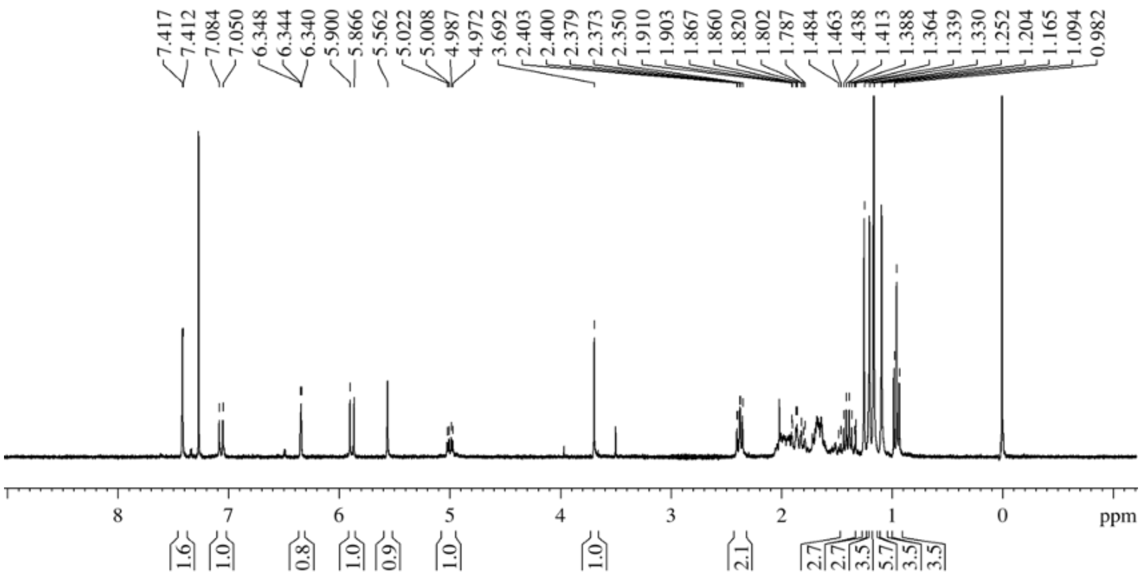

Figure S59: ^{1}H NMR ($CDCl_3$, 300 MHz) spectrum of new 7β-pentanoyloxygedunin **9**.

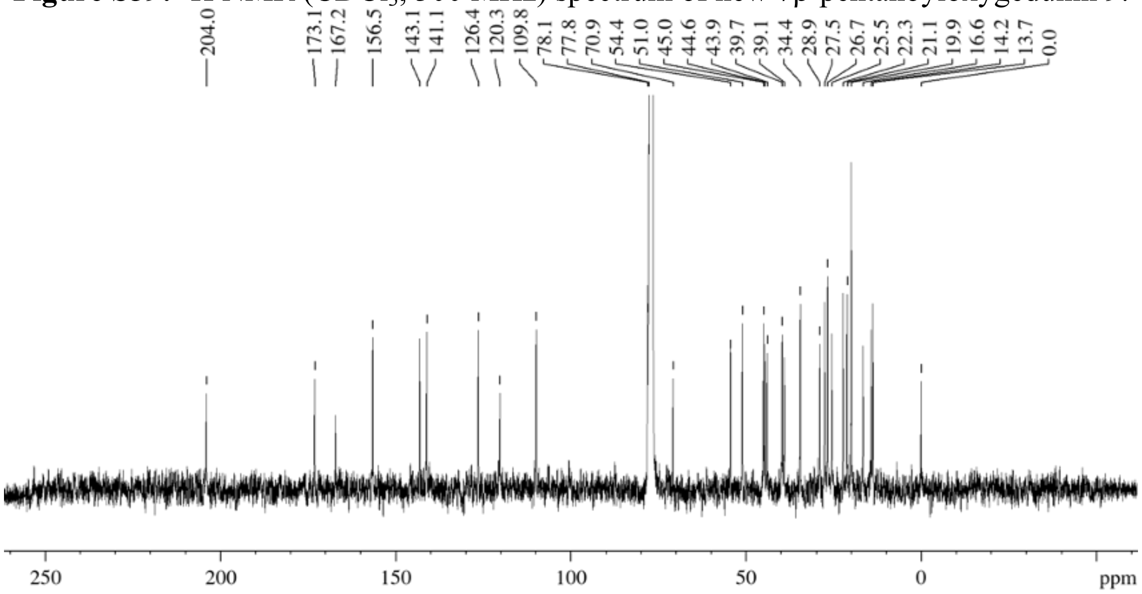

Figure S60: ^{13}C NMR ($CDCl_3$, 75 MHz) spectrum of new 7β-pentanoyloxygedunin **9**.

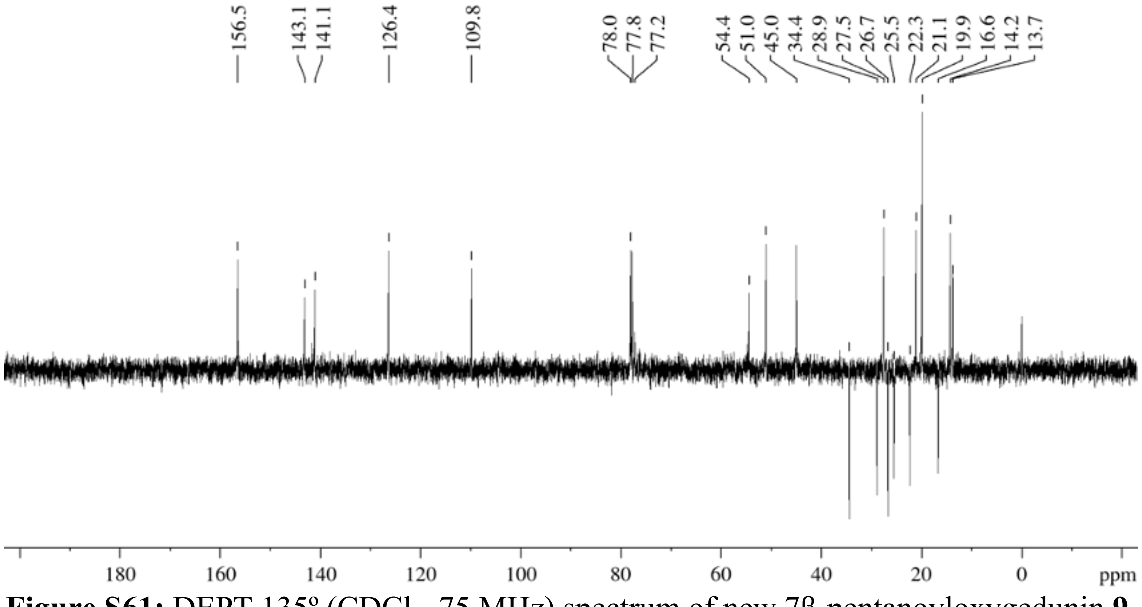

Figure S61: DEPT 135º ($CDCl_3$, 75 MHz) spectrum of new 7β-pentanoyloxygedunin **9**.

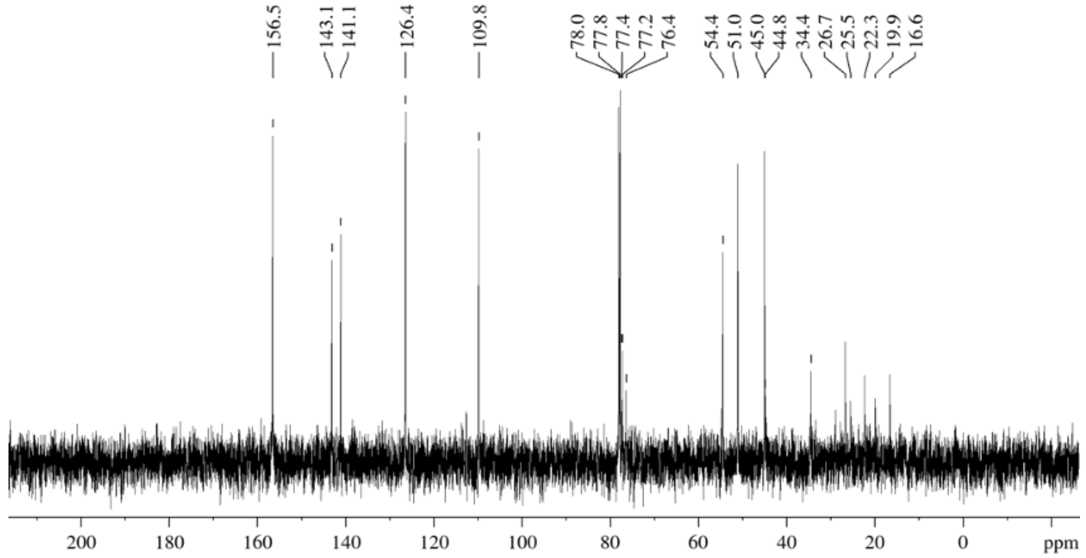

Figure S62: DEPT 90º ($CDCl_3$, 75 MHz) spectrum of new 7β-pentanoyloxygedunin **9**.

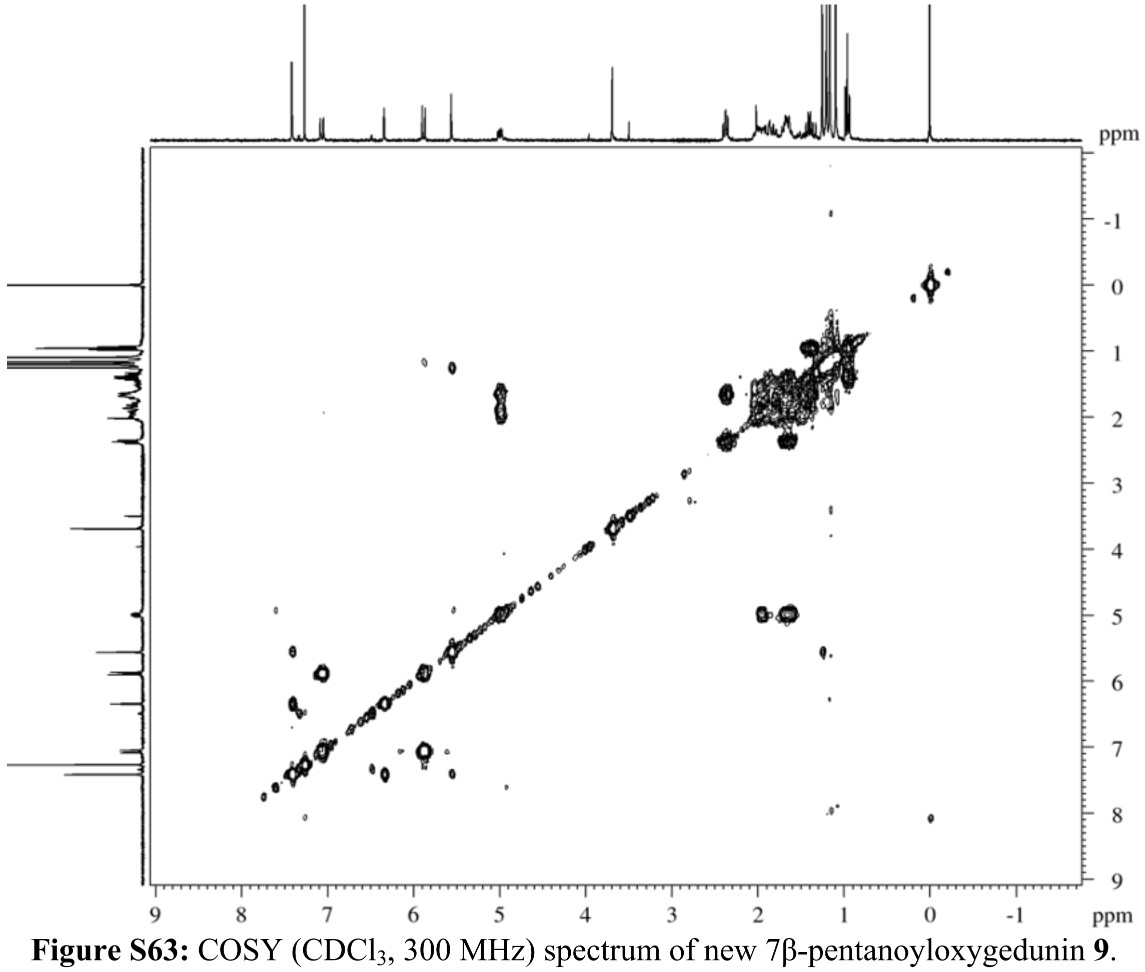

Figure S63: COSY ($CDCl_3$, 300 MHz) spectrum of new 7β-pentanoyloxygedunin **9**.

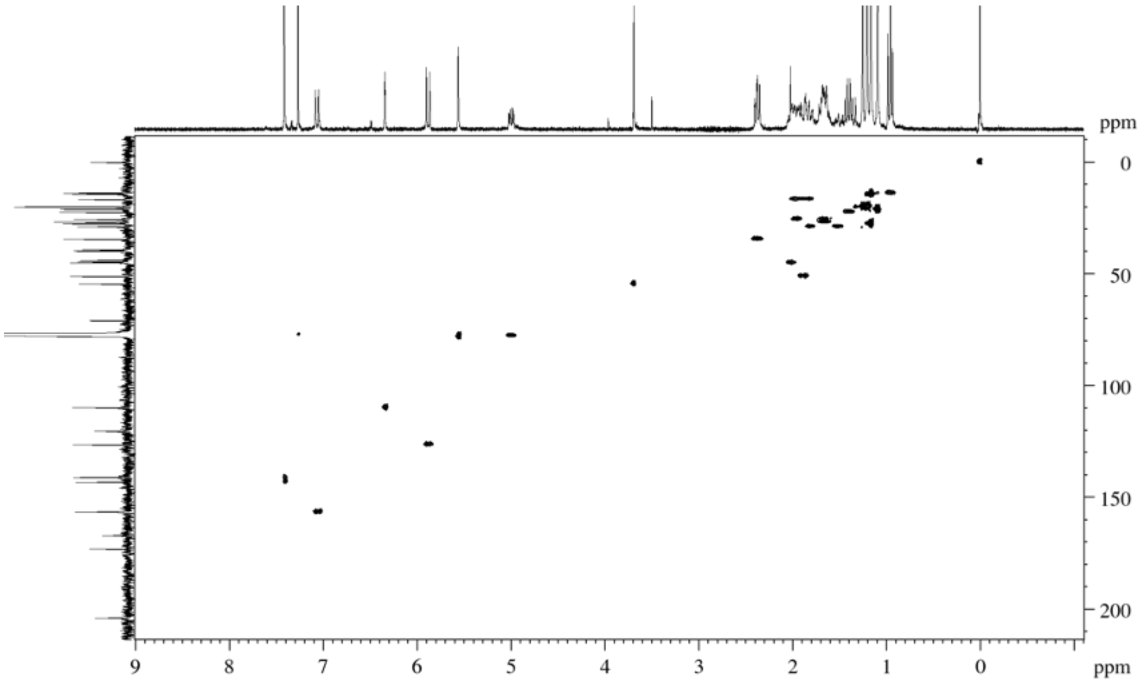

Figure S64: HSQC ($CDCl_3$, 300; 75 MHz) spectrum of new 7β-pentanoyloxygedunin **9**.

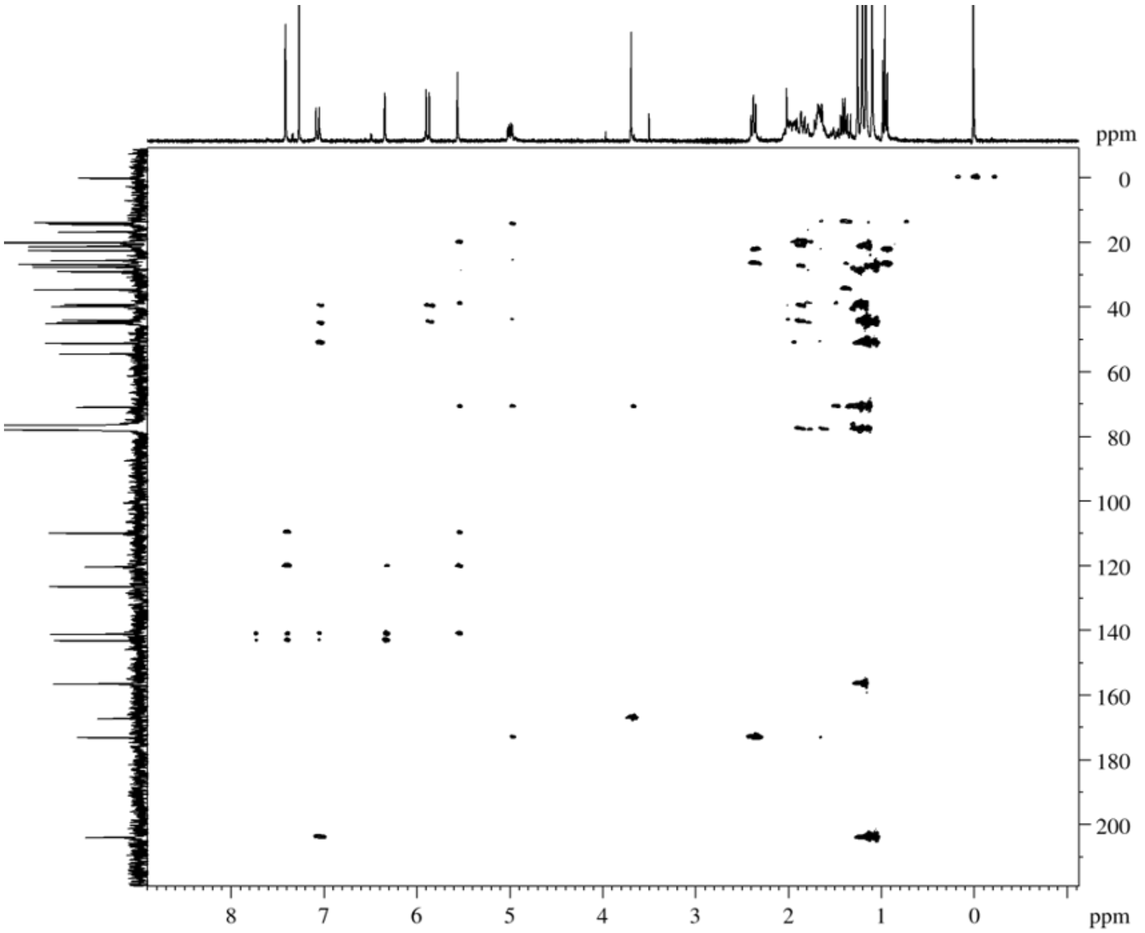

Figure S65: HMBC ($CDCl_3$, 300; 75 MHz) spectrum of new 7β-pentanoyloxygedunin **9**.

6α-Hydroxygedunin (11):

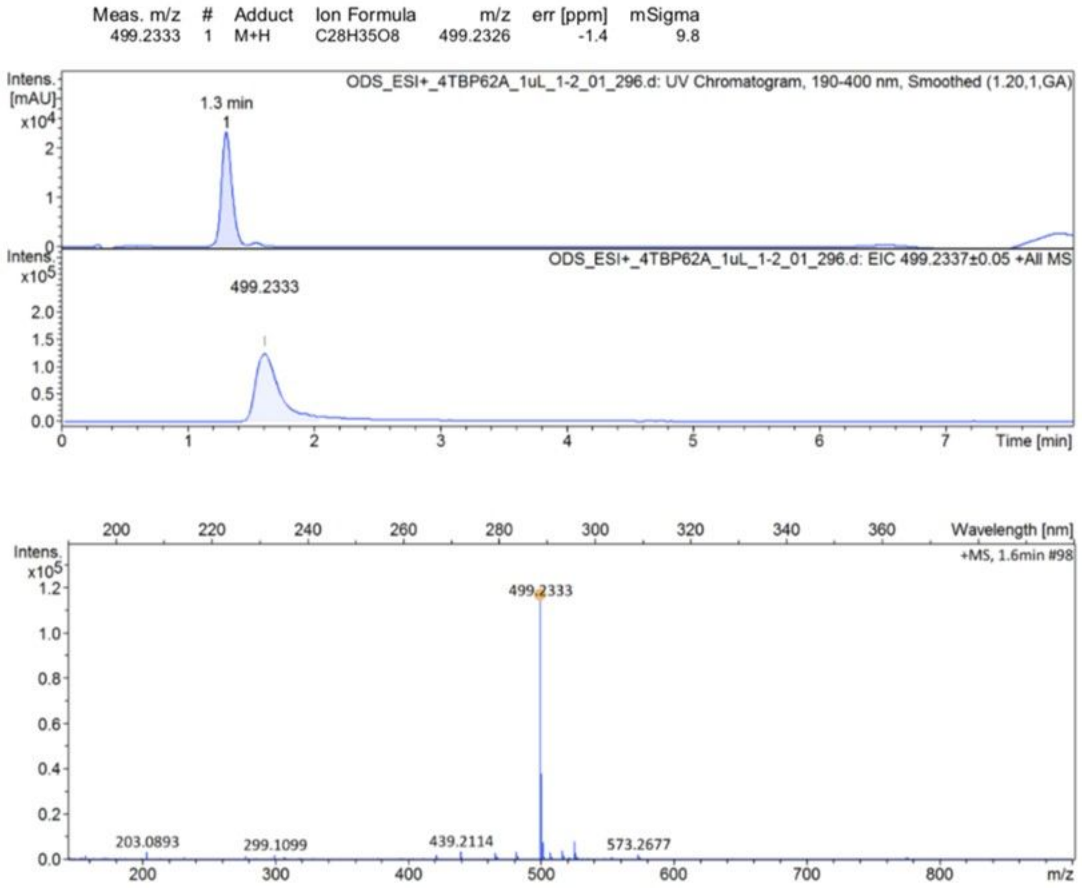

Figure S66: UFLC-PDA-ESI-(+)-HRMS data for 6α-hydroxygedunin (**11**).

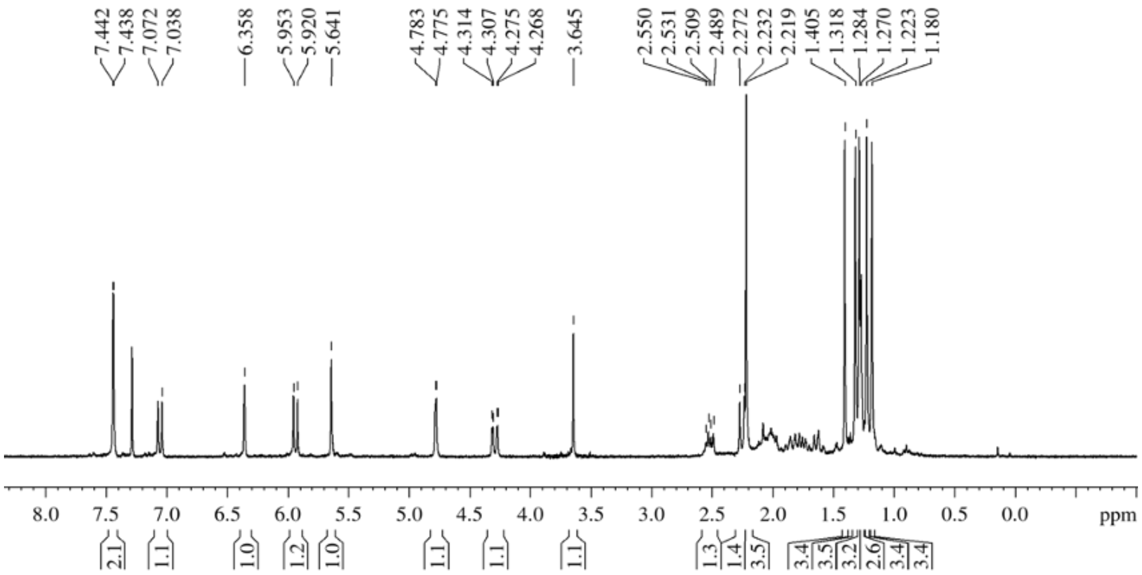

Figure S67: ^{1}H NMR ($CDCl_3$, 300 MHz) spectrum of 6α-hydroxygedunin (**11**).

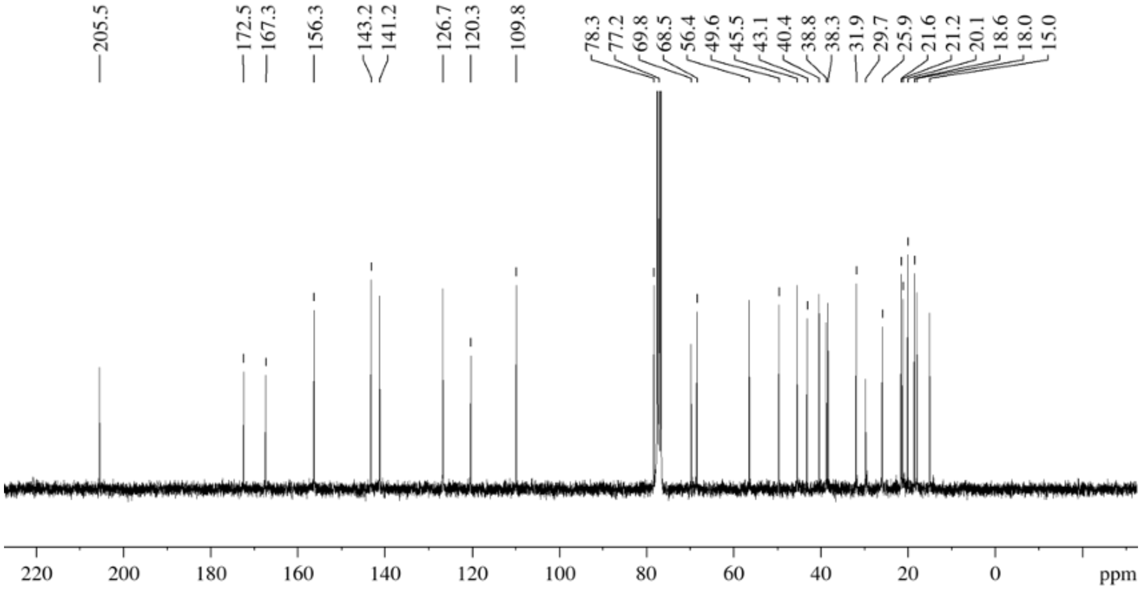

Figure S68: ^{13}C NMR ($CDCl_3$, 75 MHz) spectrum of 6α-hydroxygedunin (**11**).

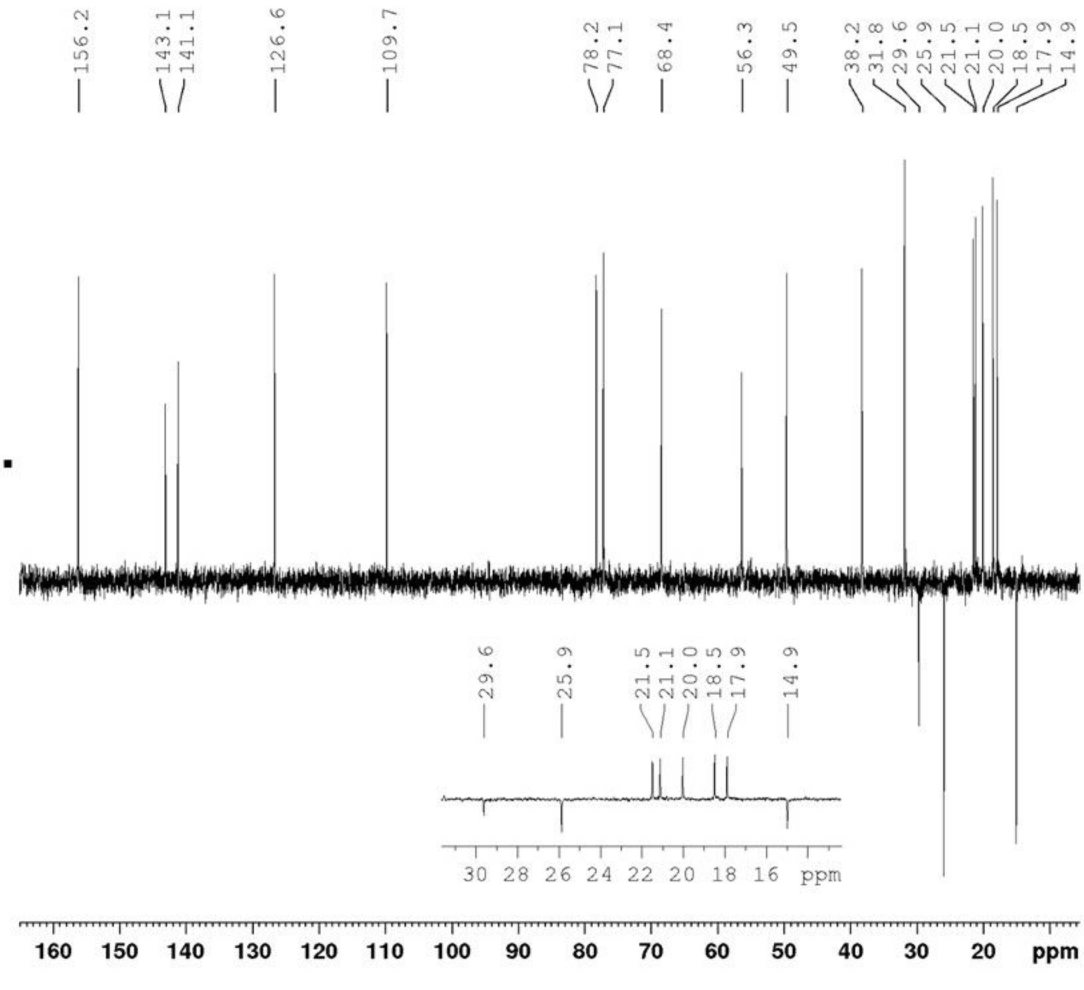

Figure S69: DEPT 135º ($CDCl_3$, 75 MHz) spectrum of 6α-hydroxygedunin (**11**).

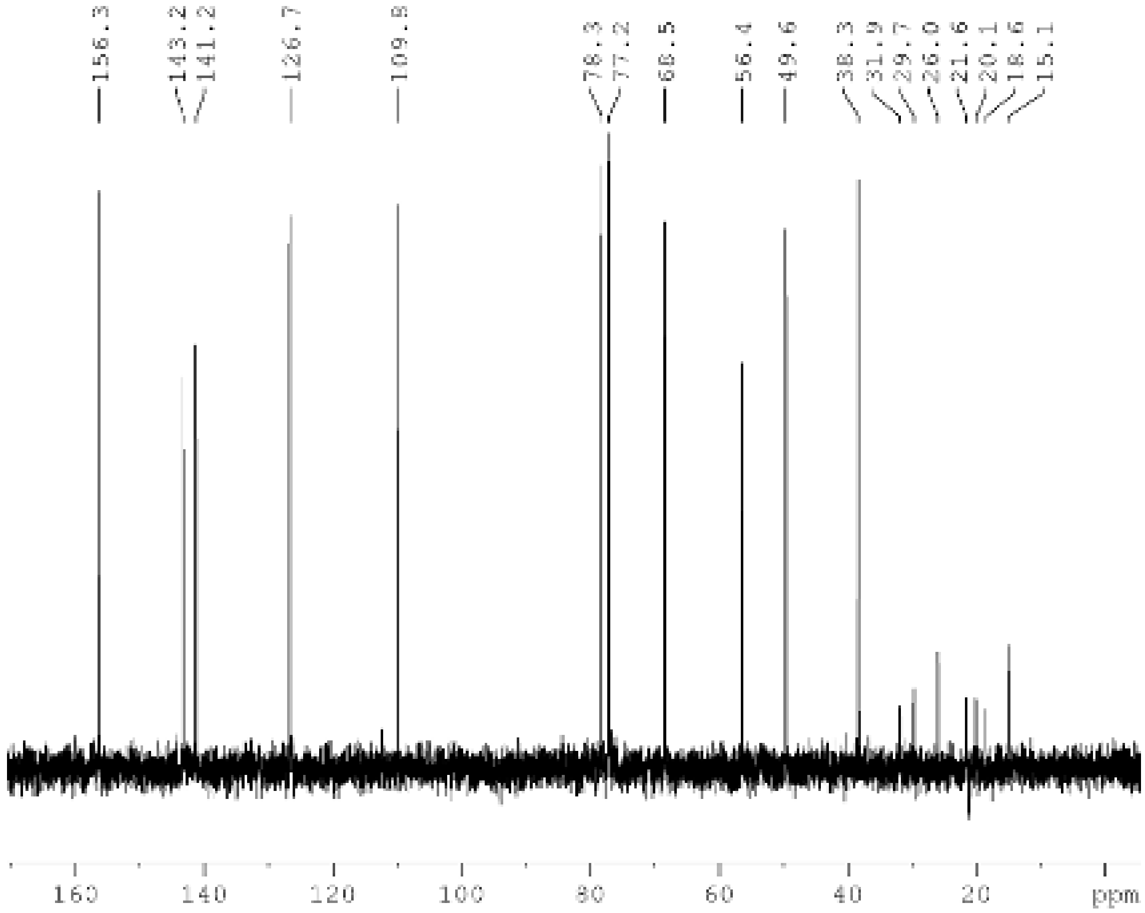

Figure S70: DEPT 90º ($CDCl_3$, 75 MHz) spectrum of 6α-hydroxygedunin (**11**).

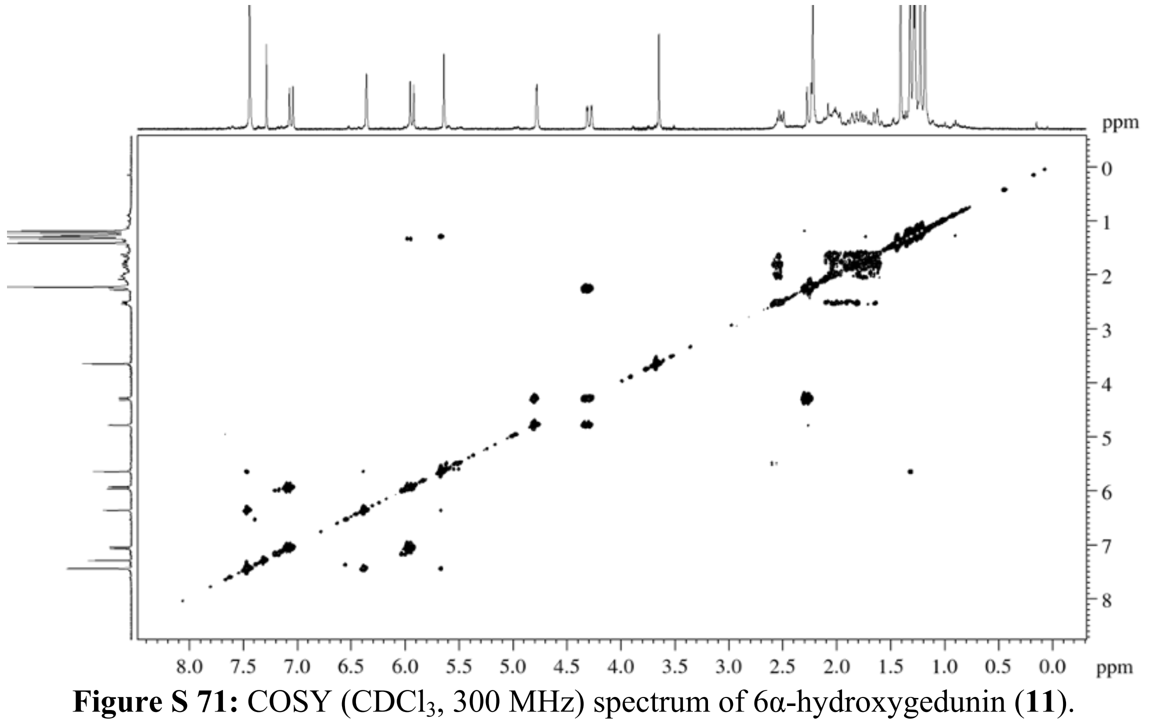

Figure S 71: COSY ($CDCl_3$, 300 MHz) spectrum of 6α-hydroxygedunin (**11**).

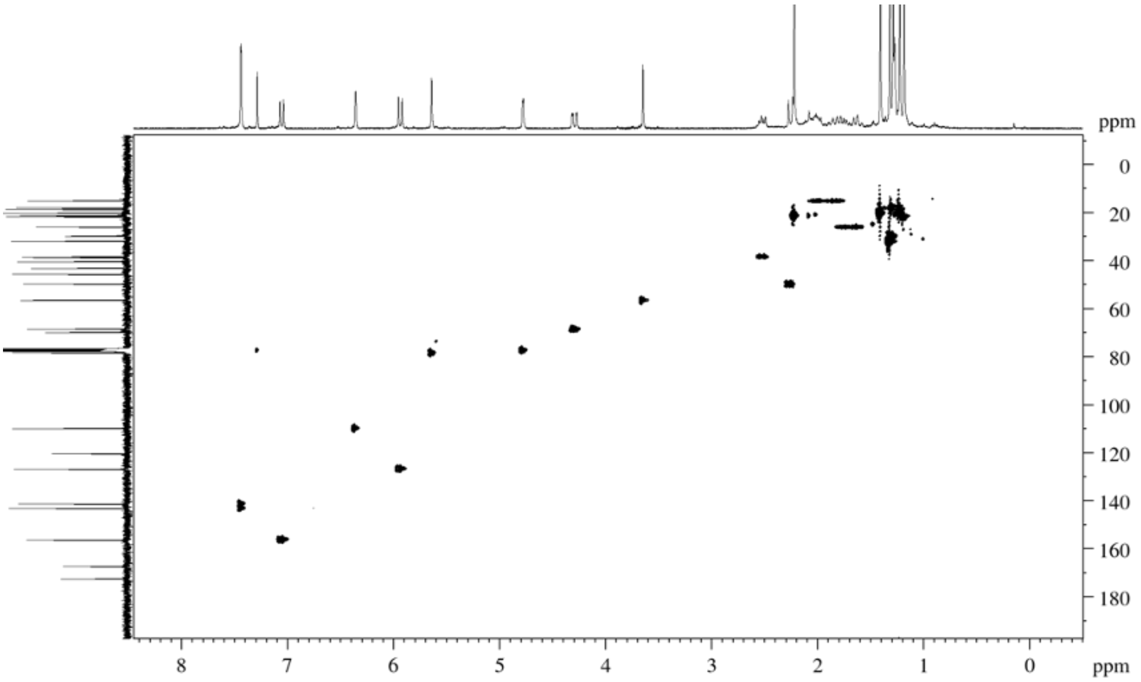

Figure S 72: HSQC ($CDCl_3$, 300; 75 MHz) spectrum of 6α-hydroxygedunin (**11**).

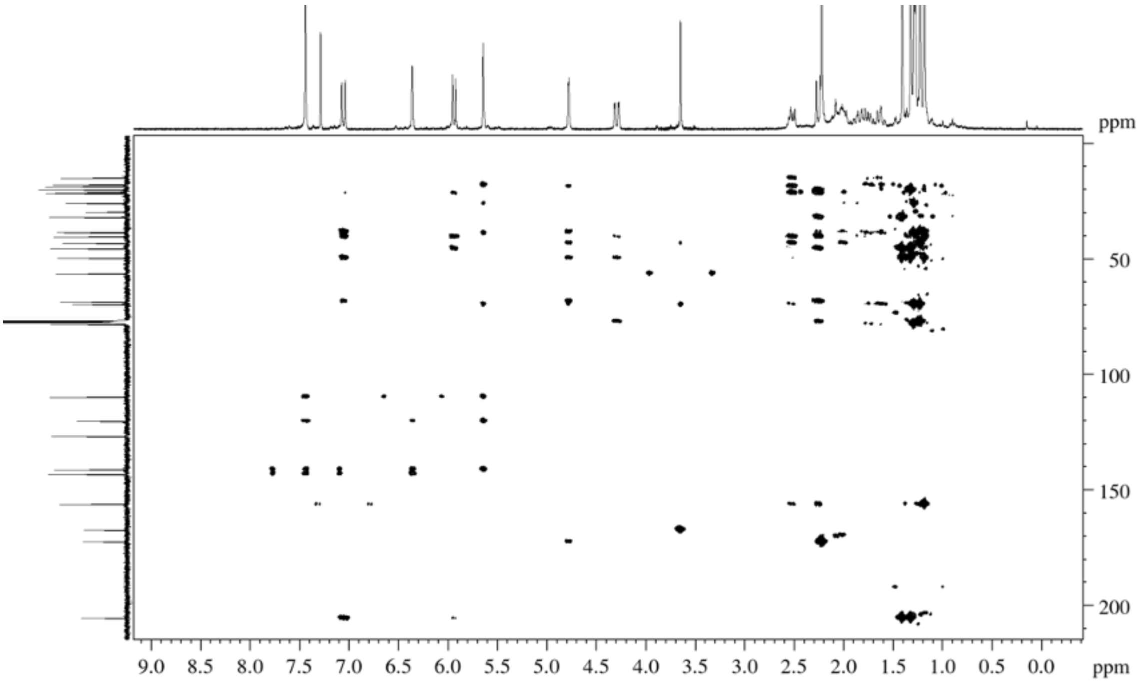

Figure S73: HMBC ($CDCl_3$, 300; 75 MHz) spectrum of 6α-hydroxygedunin (**11**).

7-deacetyl-6α-butanoyloxygedunin (13):

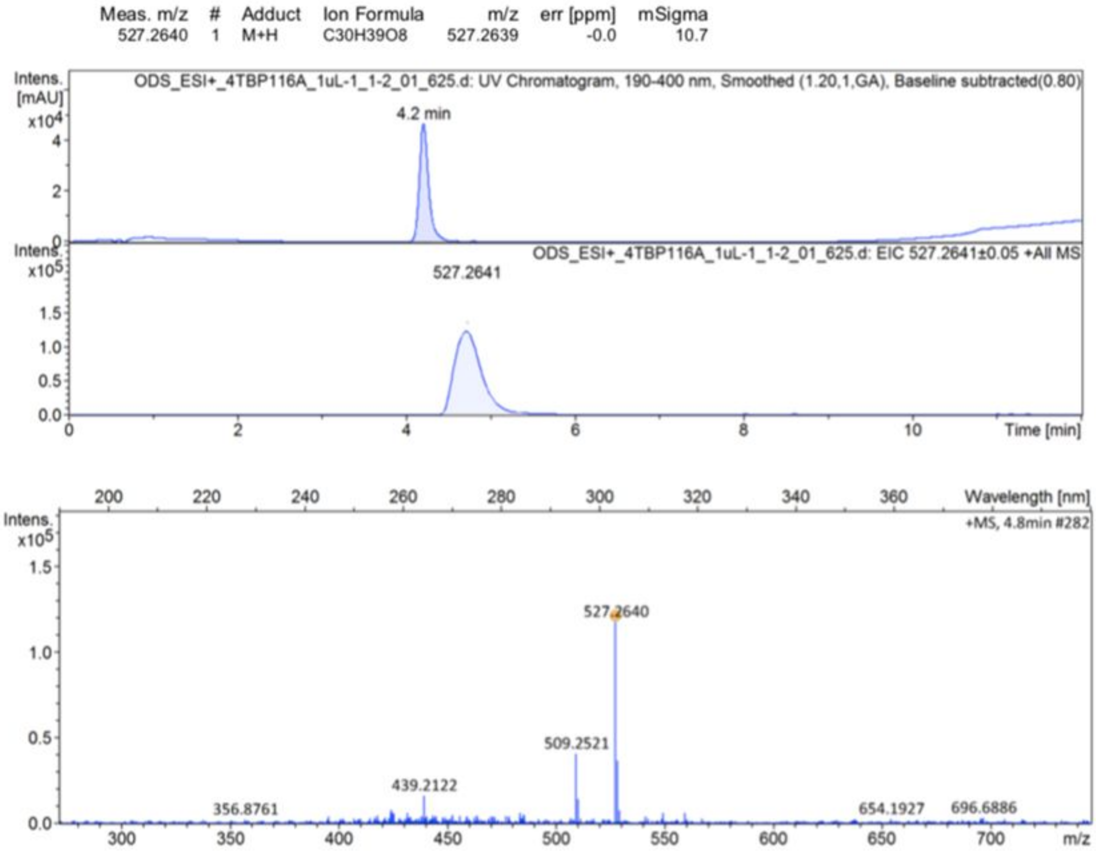

Figure S74: UFLC-ESI-(+)-HRMS data for 7-deacetyl-6α-butanoyloxygedunin (**13**).

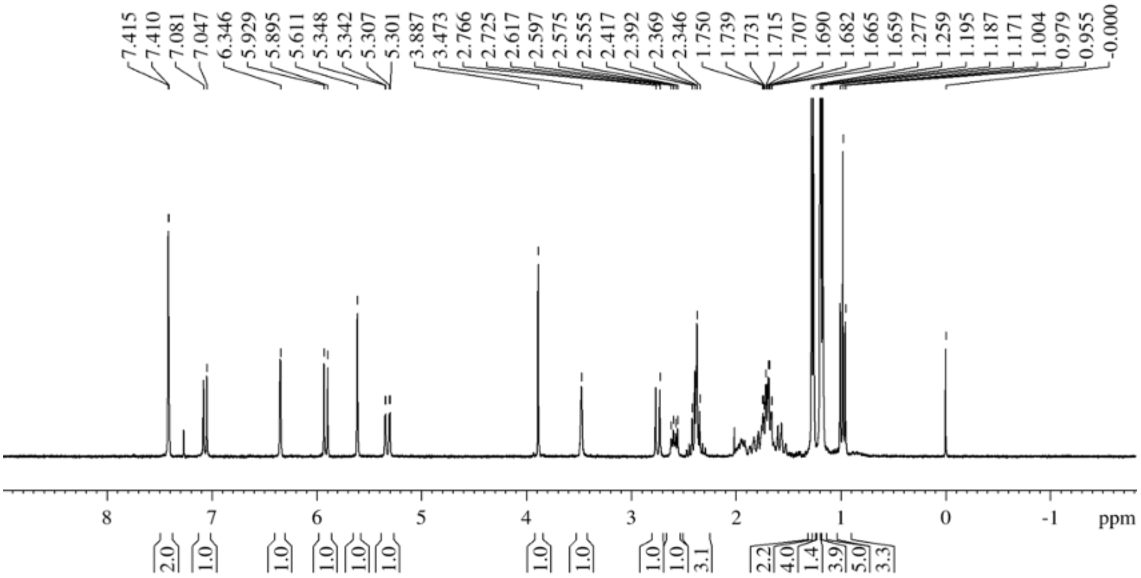

Figure S75: ^{1}H NMR ($CDCl_3$, 300 MHz) spectrum of 6α-butanoyloxygedunin **13**.

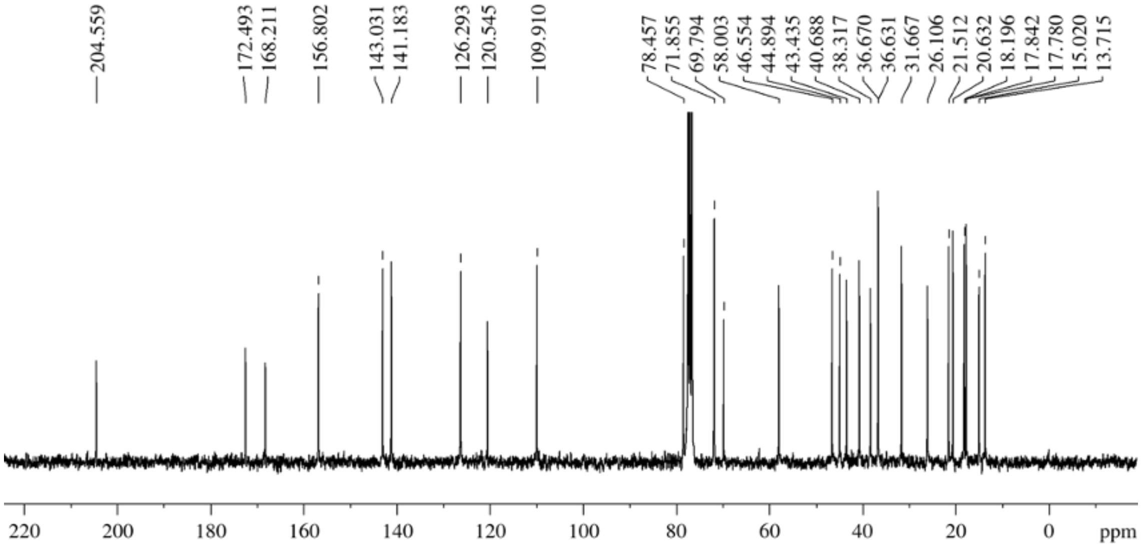

Figure S76: ^{13}C NMR ($CDCl_3$, 75 MHz) spectrum of 6α-butanoyloxygedunin **13**.

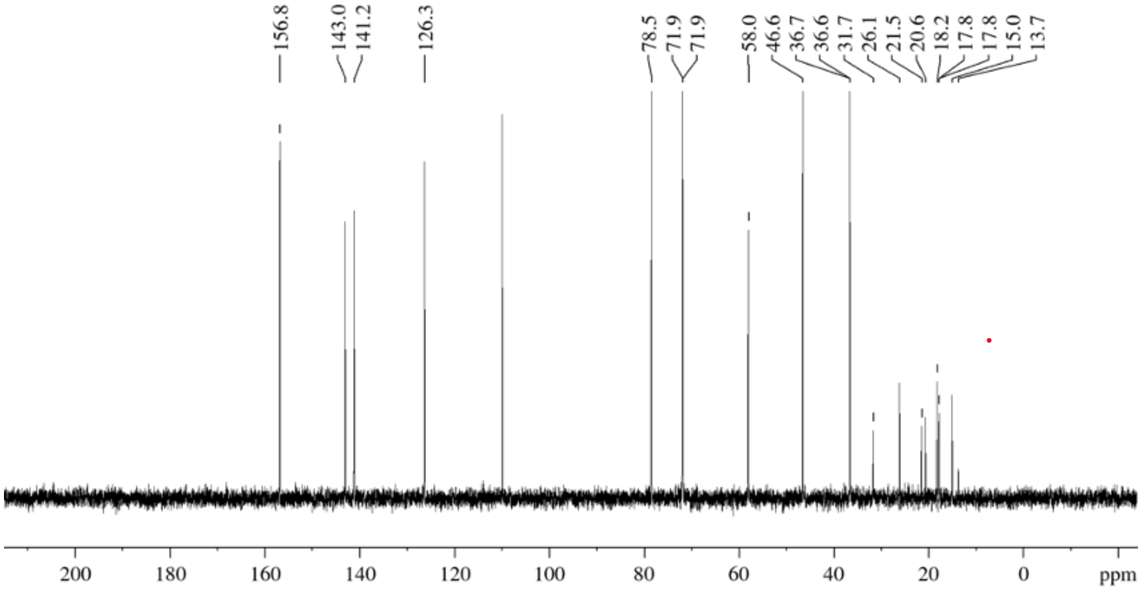

Figure S77: DEPT 90º ($CDCl_3$, 75 MHz) spectrum of 6α-butanoyloxygedunin **13**.

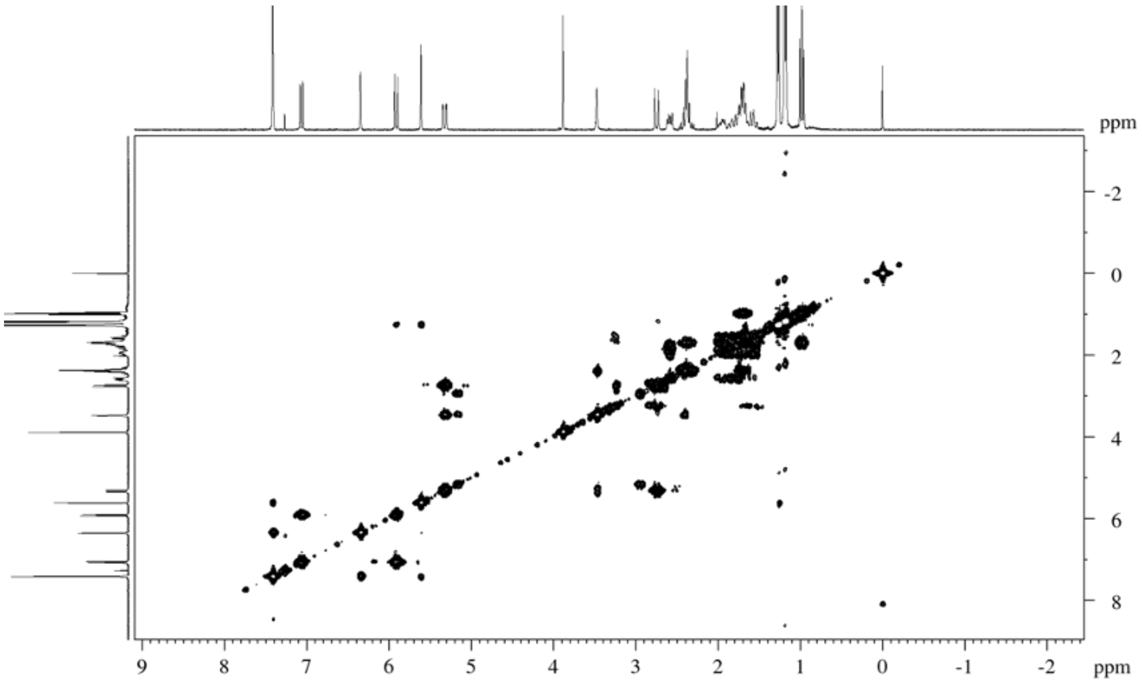

Figure S78: COSY ($CDCl_3$, 300 MHz) spectrum of 6α-butanoyloxygedunin **13**.

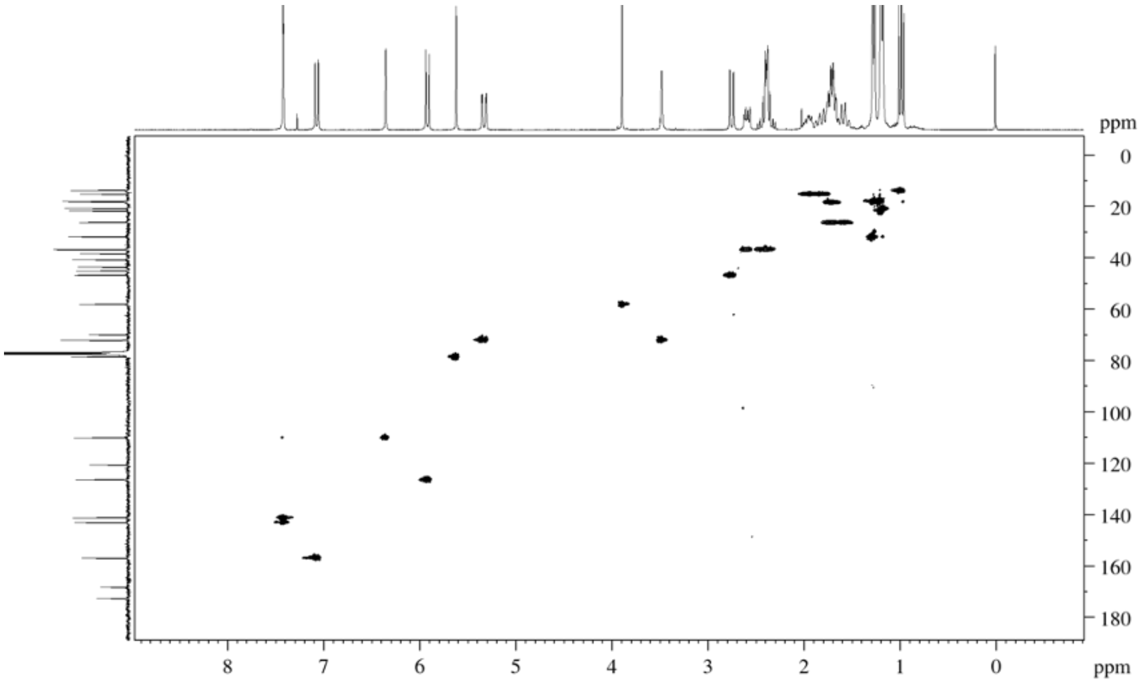

Figure S79: HSQC ($CDCl_3$, 300; 75 MHz) spectrum of 6α-butanoyloxygedunin **13**.

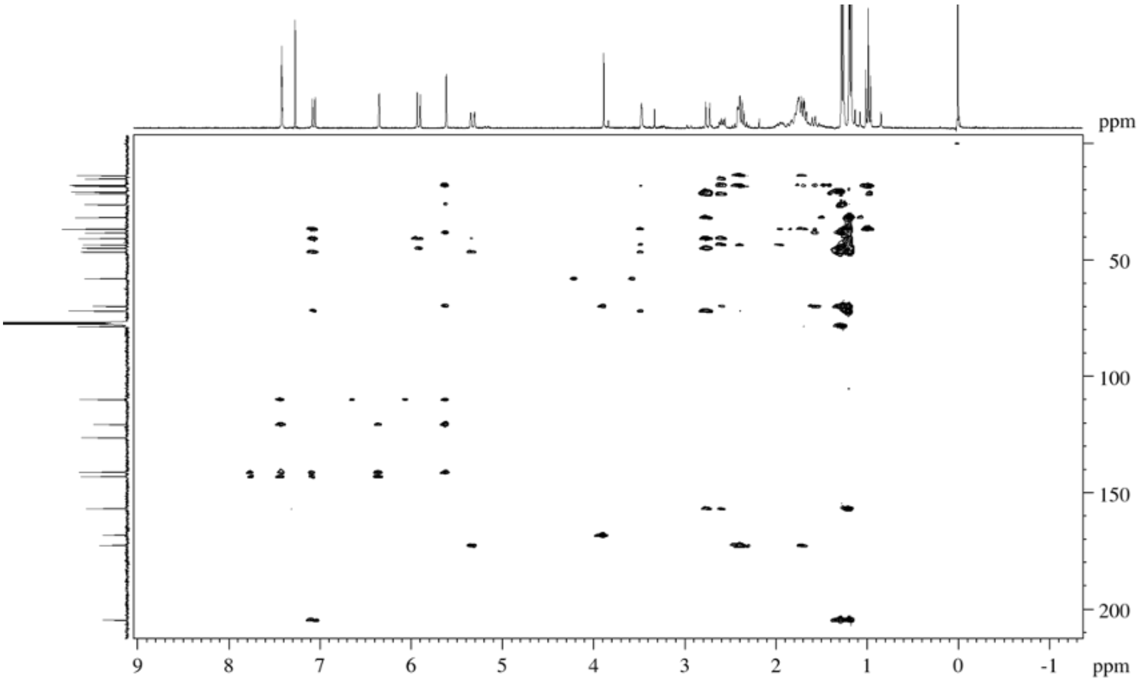

Figure S80: HMBC ($CDCl_3$, 300; 75 MHz) spectrum of 6α-butanoyloxygedunin **13**.

7-Deacetyl-6α-benzoxygedunin (14):

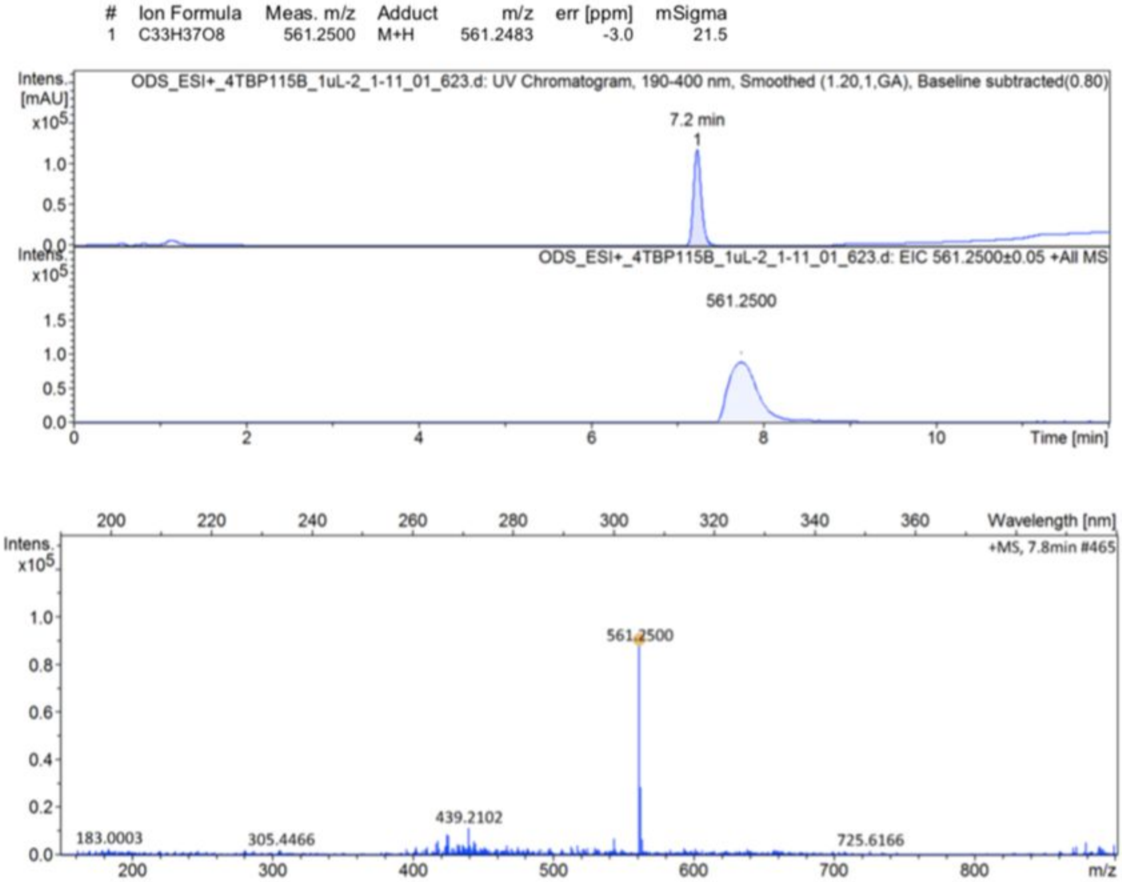

Figure S81: UFLC-PDA-ESI-(+)-HRMS data for new 6α-benzoxygedunin **14**.

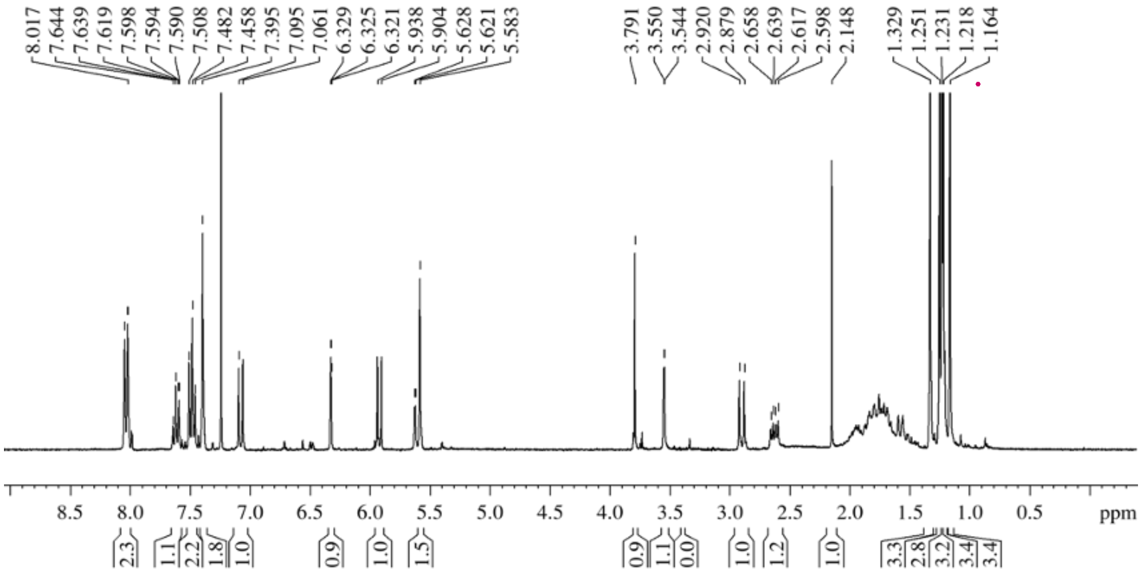

Figure S82: ^{1}H NMR ($CDCl_3$, 300 MHz) spectrum of new 6α-benzoxygedunin **14**.

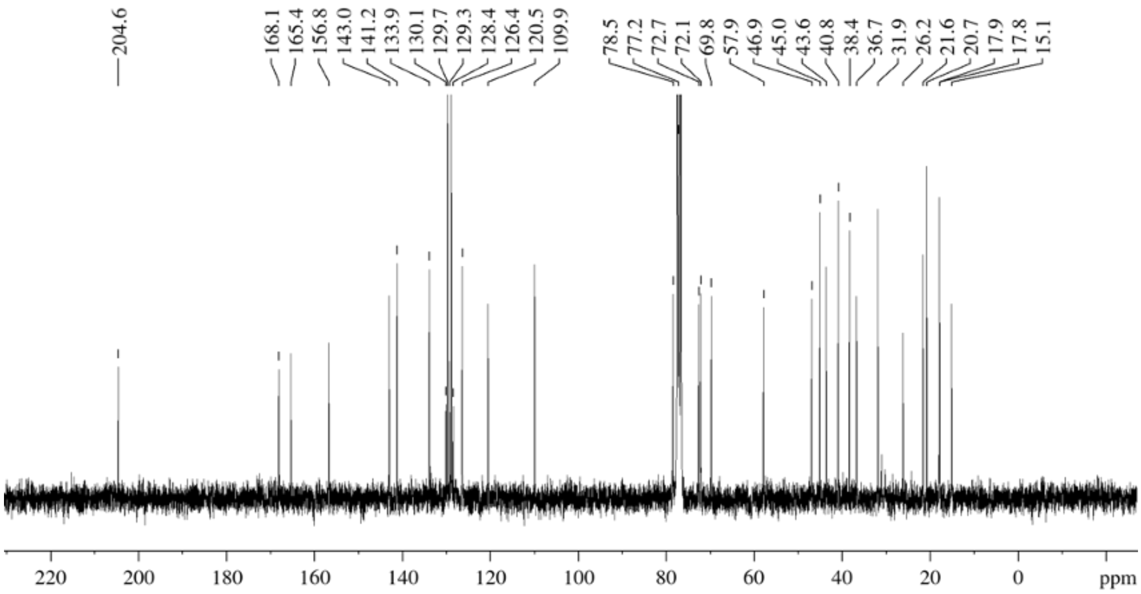

Figure S83: ^{13}C NMR ($CDCl_3$, 75 MHz) spectrum of new 6α-benzoxygedunin **14**.

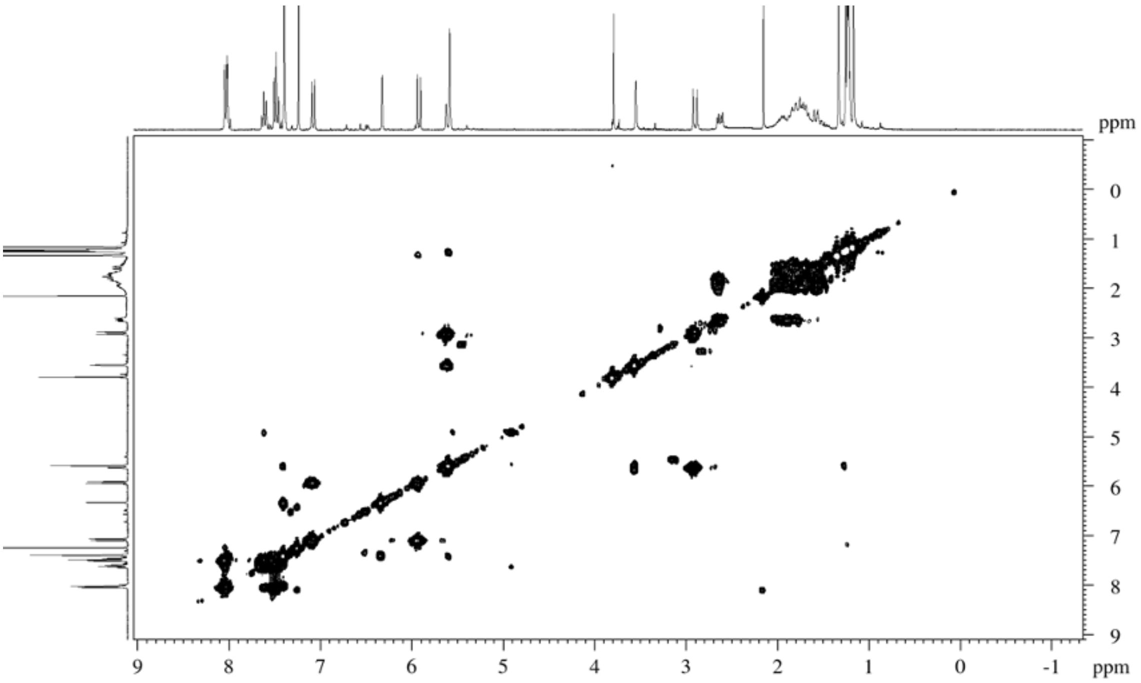

Figure S84: COSY ($CDCl_3$, 300 MHz) spectrum of new 6α-benzoxygedunin **14**.

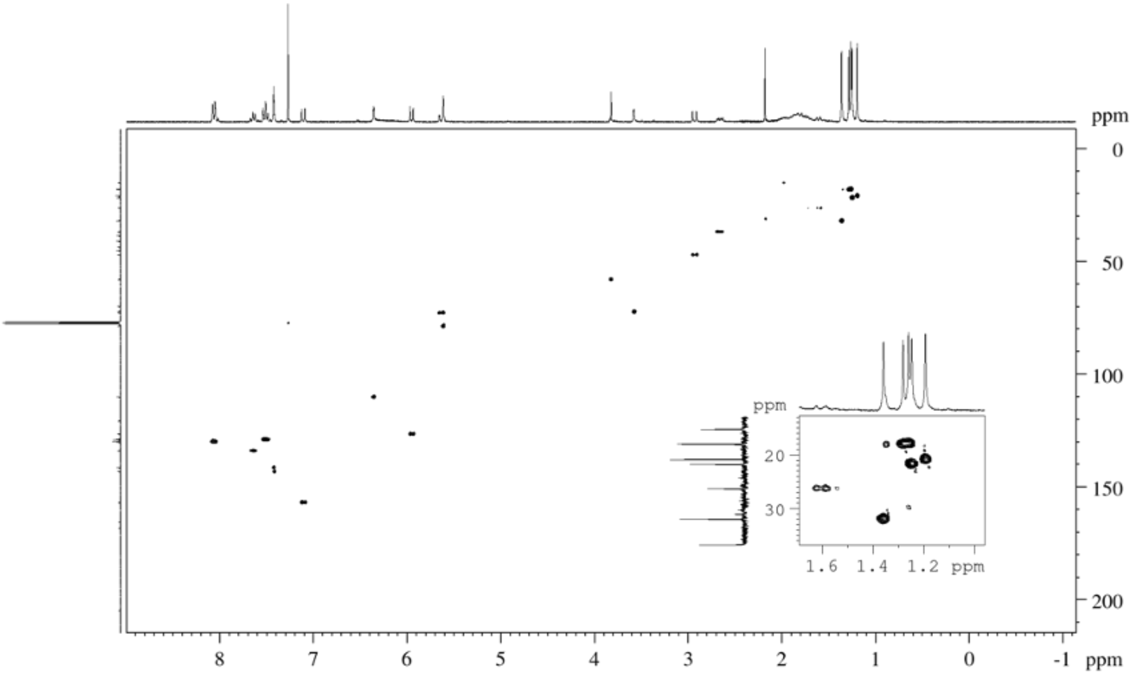

Figure S85: HSQC ($CDCl_3$, 300; 75 MHz) spectrum of new 6α-benzoxygedunin **14**.

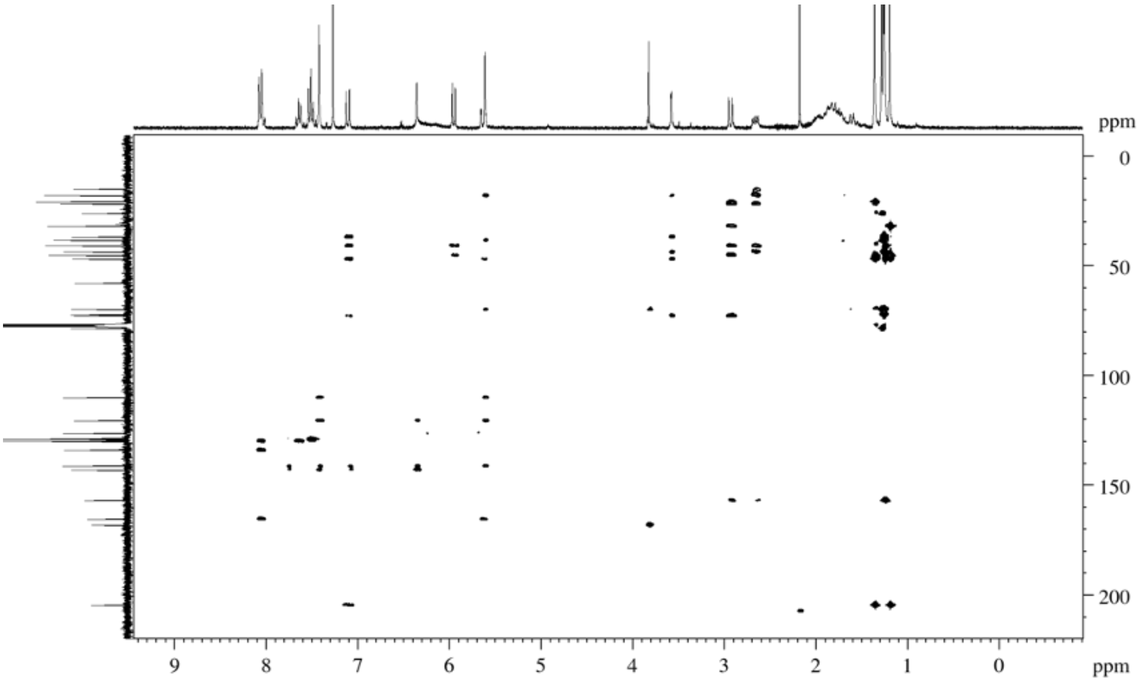

Figure S86: HMBC ($CDCl_3$, 300; 75 MHz) spectrum of new 6α-benzoxygedunin **14**.

7-Deacetyl-6α-heptanoyloxygedunin (15):

Meas. m/z	#	Adduct	Ion Formula	m/z	err [ppm]	mSigma
525.3209	1	M+H	C32H45O6	525.3211	0.4	14.3
551.3012	1	M+H	C33H43O7	551.3003	-1.6	1.3
569.3122	1	M+H	C33H45O8	569.3109	-2.3	4.0

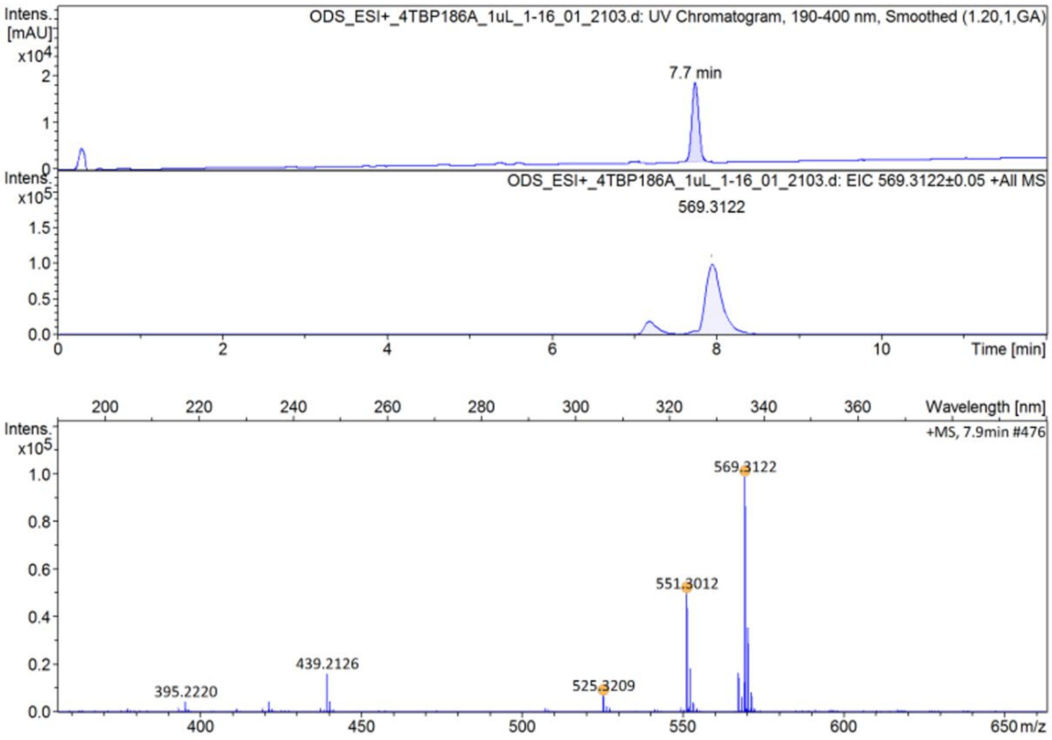

Figure S87: UFLC-PDA-ESI-(+)-HRMS data for new 6α-heptanoyloxygedunin **15**.

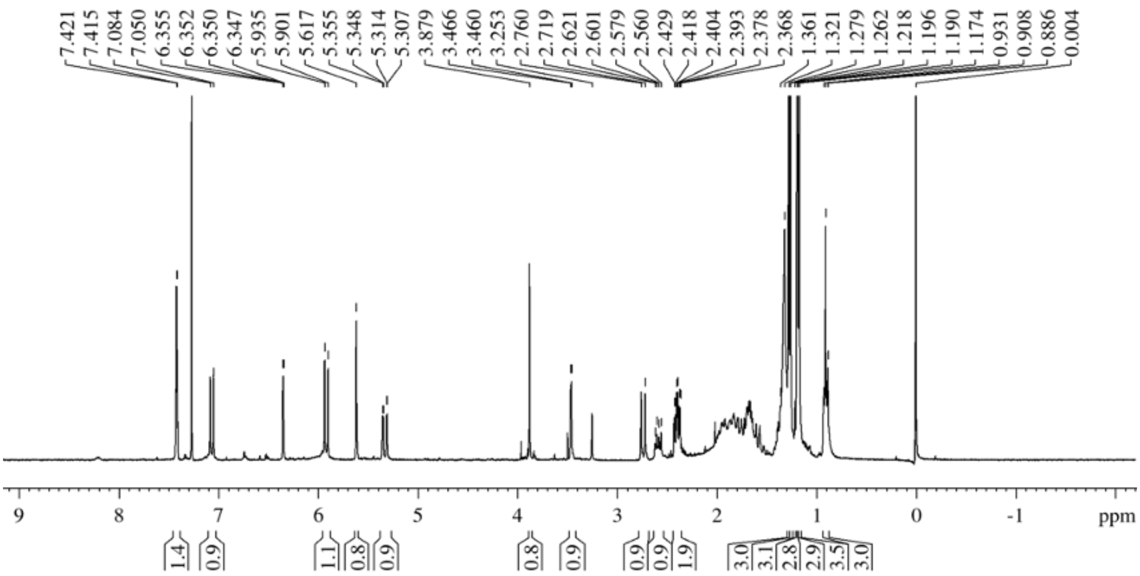

Figure S88: ^{1}H NMR ($CDCl_3$, 300 MHz) spectrum of new 6α-heptanoyloxygedunin **15**.

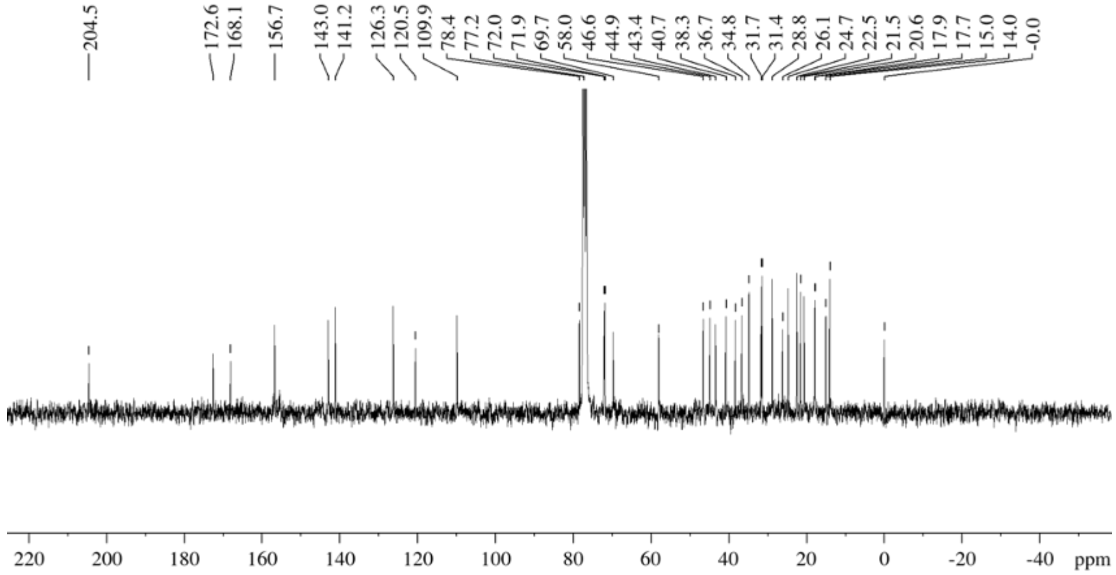

Figure S89: ^{13}C NMR ($CDCl_3$, 75 MHz) spectrum of new 6α-heptanoyloxygedunin **15**.

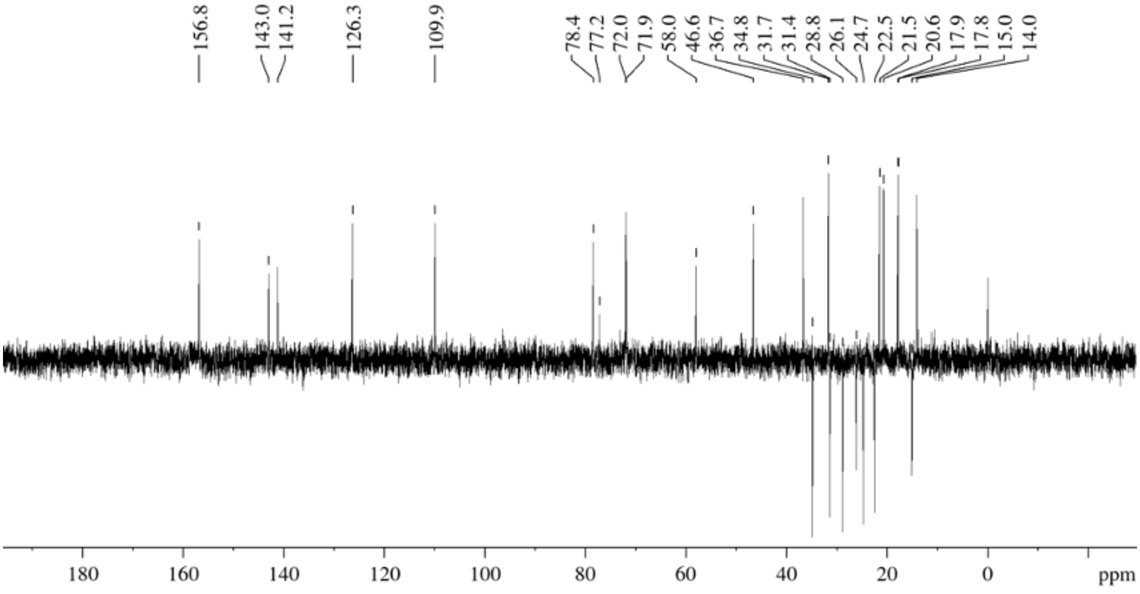

Figure S90: DEPT 135º ($CDCl_3$, 75 MHz) spectrum of new 6α-heptanoyloxygedunin **15**.

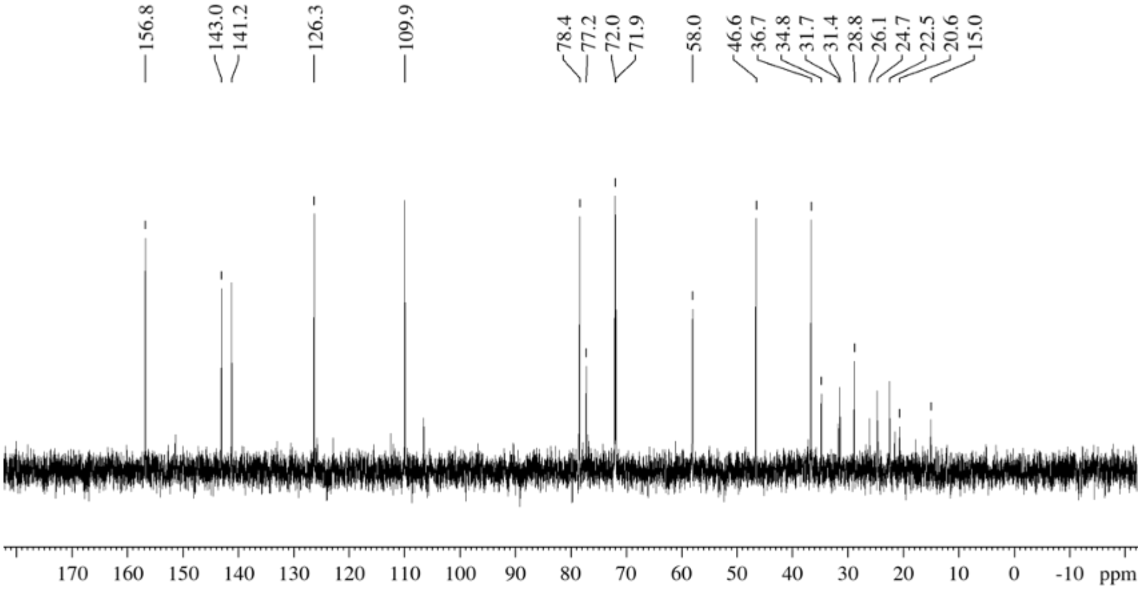

Figure S91: DEPT 90º ($CDCl_3$, 75 MHz) spectrum of new 6α-heptanoyloxygedunin **15**.

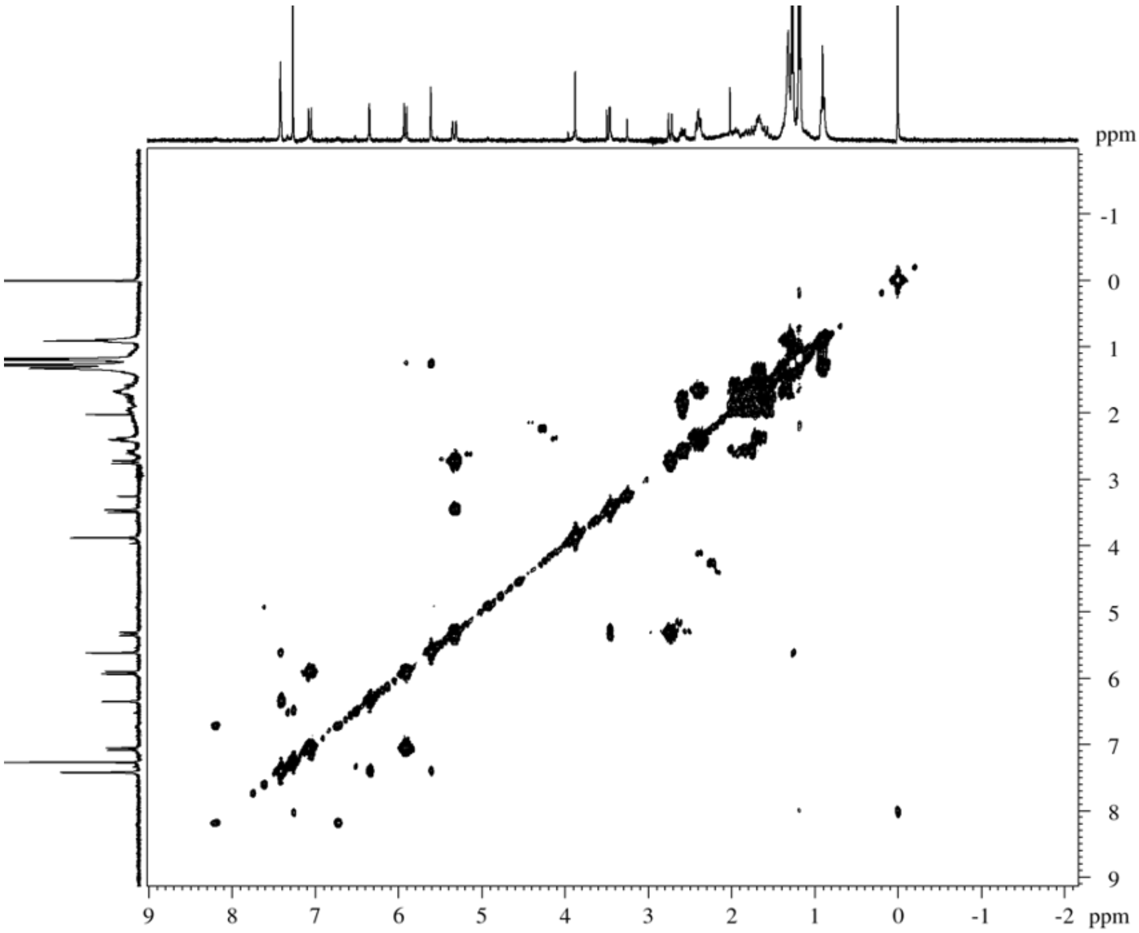

Figure S92: COSY ($CDCl_3$, 300 MHz) spectrum of new 6α-heptanoyloxygedunin **15**.

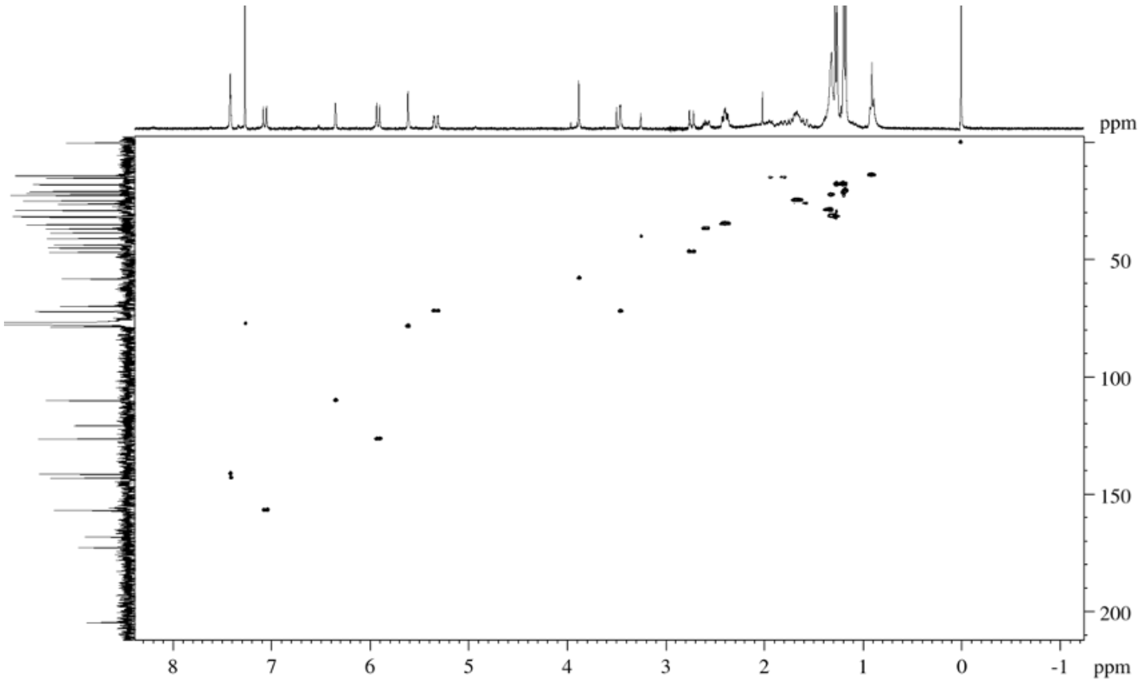

Figure S93: HSQC ($CDCl_3$, 300; 75 MHz) spectrum of new compound **15**.

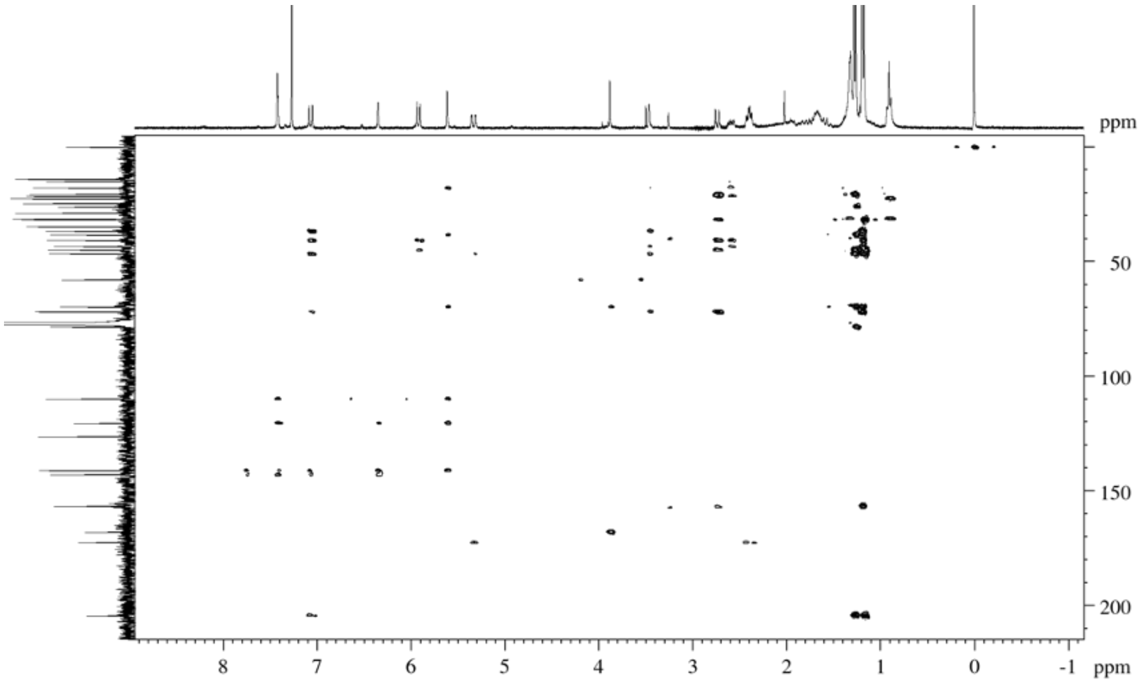

Figure S94: HMBC ($CDCl_3$, 300; 75 MHz) spectrum of new compound **15**.

3-Deoxo-1,2-dihydro-7-deacetyl-3α-hydroxy-7-*epi*-gedunin (16):

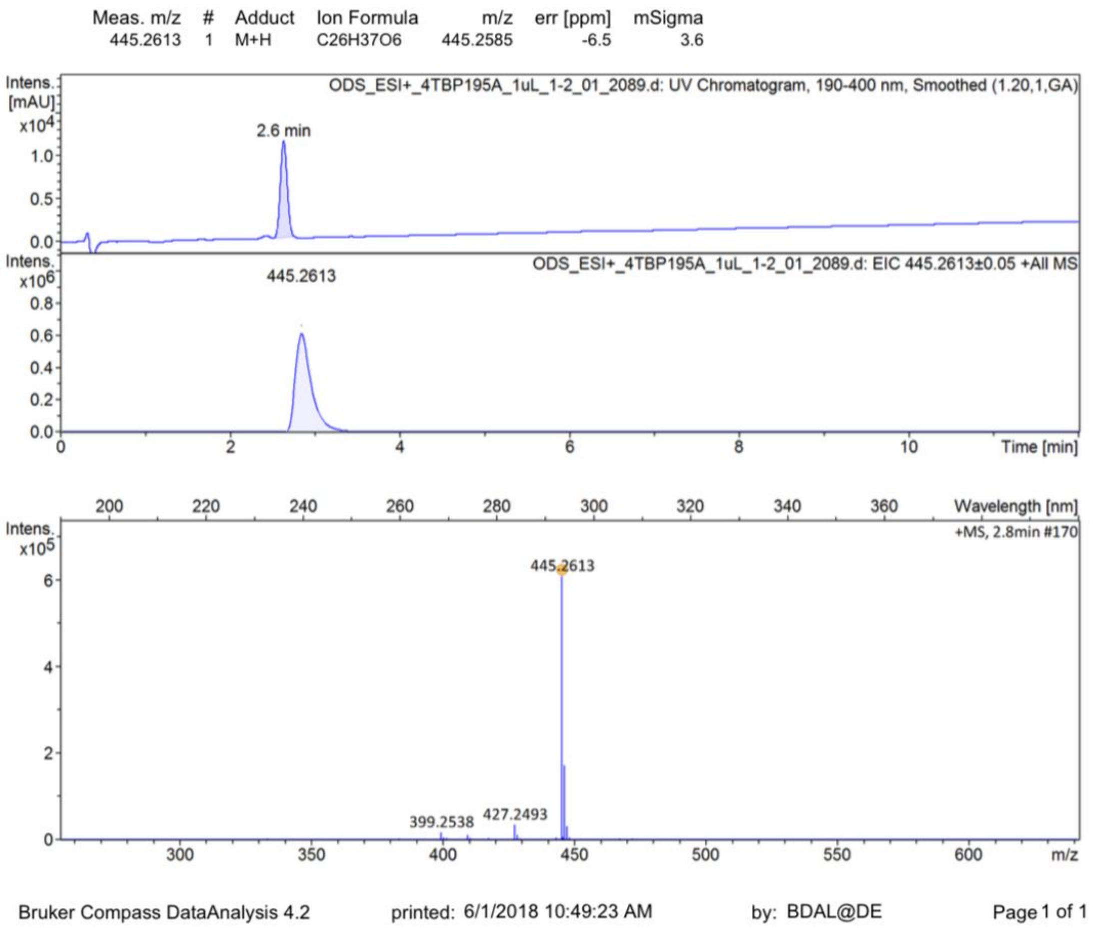

Figure S95: UFLC-PDA-ESI-(+)-HRMS data for new compound **16**.

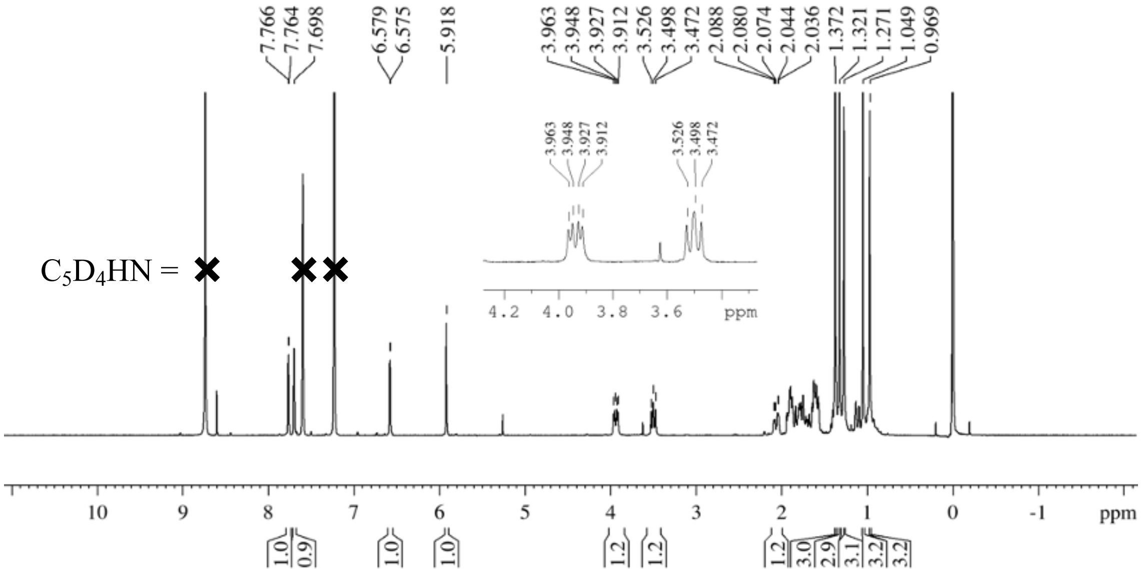

Figure S96: ^{1}H NMR (C_5D_5N, 300 MHz) spectrum of new compound **16**.

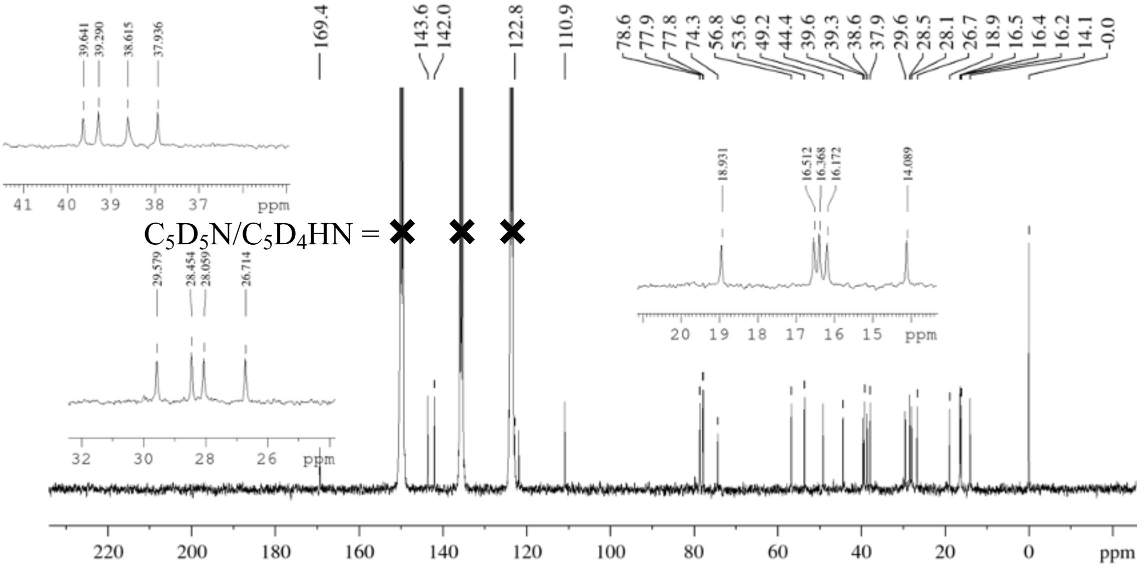

Figure S97: ^{13}C NMR (C_5D_5N, 75 MHz) spectrum of new compound **16**.

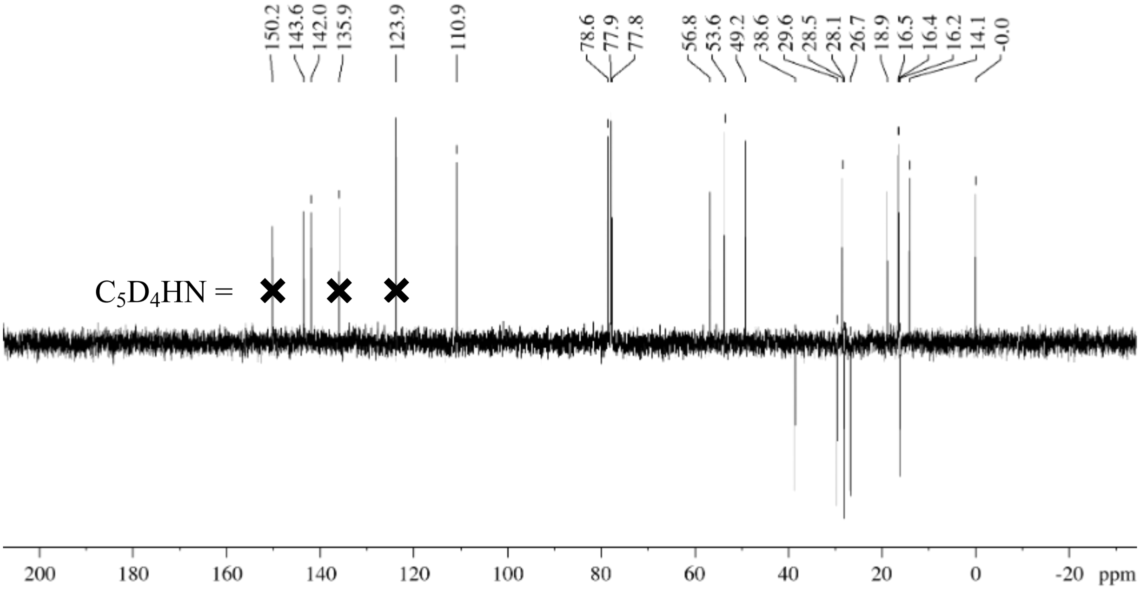

Figure S98: DEPT 135º (C_5D_5N, 75 MHz) spectrum of new compound **16**.

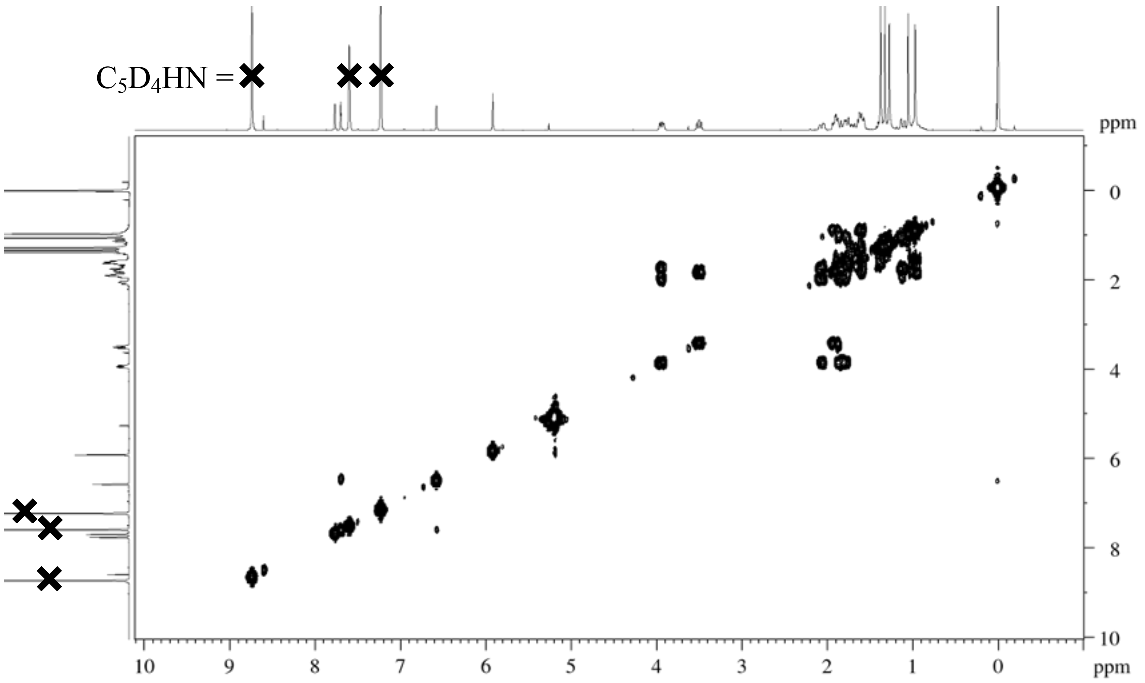

Figure S99: COSY (C_5D_5N, 300 MHz) spectrum of new compound **16**.

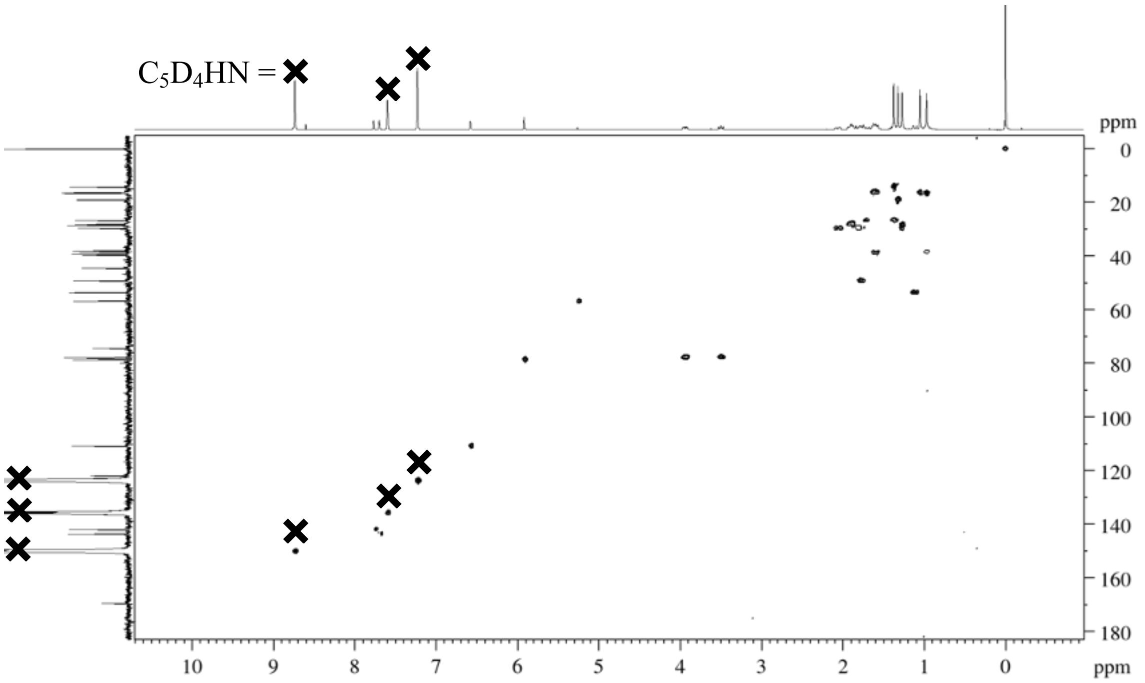

Figure S100: HSQC (C_5D_5N, 300; 75 MHz) spectrum of new compound **16**.

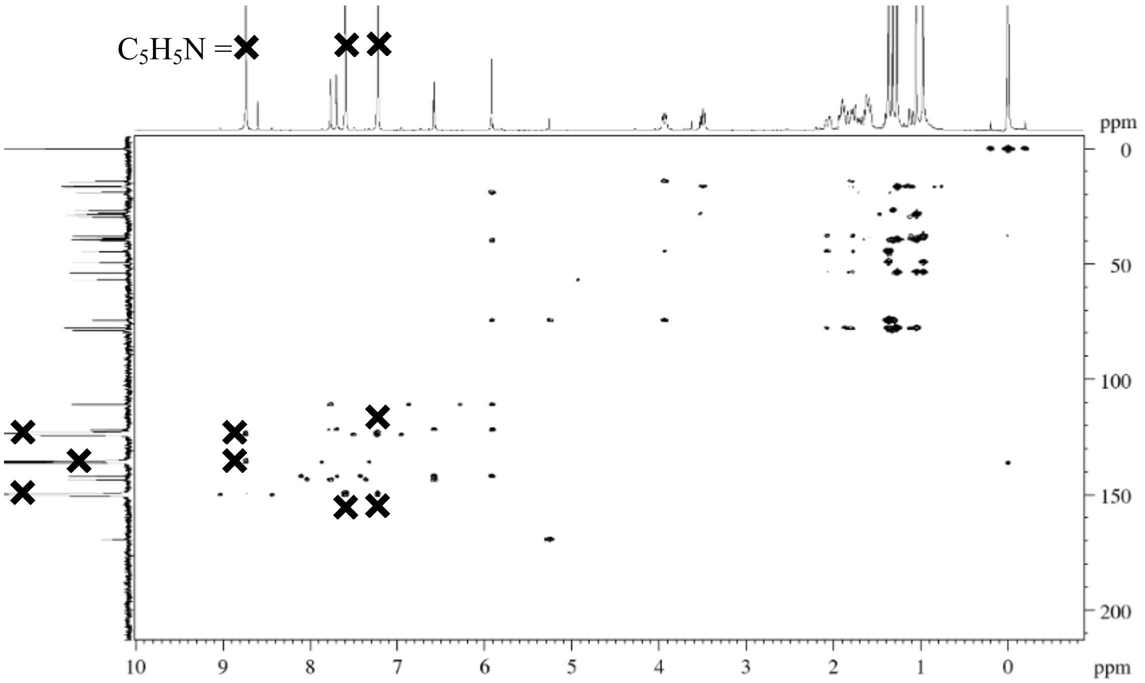

Figure S101: HMBC (C_5D_5N, 300; 75 MHz) spectrum of new compound **16**.

Reference

(1) Pereira, T. B.; Rocha e Silva, L. F.; Amorim, R. C.; Melo, M. R.; Zacardi de Souza, R. C.; Eberlin, M. N.; Lima, E. S.; Vasconcellos, M. C.; Pohlit, A. M. *In vitro* and *in vivo* anti-malarial activity of limonoids isolated from the residual seed biomass from *Carapa guianensis* (andiroba) oil production. *Malar. J.* **2014**, *13*, 317. https://doi.org/10.1186/1475-2875-13-317.
